# Identification of 3-(piperazinylmethyl)benzofuran derivatives as novel type II CDK2 inhibitors: design, synthesis, biological evaluation, and *in silico* insights

**DOI:** 10.1080/14756366.2022.2062337

**Published:** 2022-04-26

**Authors:** Wagdy M. Eldehna, Raed M. Maklad, Hadia Almahli, Tarfah Al-Warhi, Eslam B. Elkaeed, Mohammed A. S. Abourehab, Hatem A. Abdel-Aziz, Ahmed M. El Kerdawy

**Affiliations:** a School of Biotechnology, Badr University in Cairo, Badr City, Egypt; b Department of Pharmaceutical Chemistry, Faculty of Pharmacy, Kafrelsheikh University, Kafrelsheikh, Egypt; c Institute of Drug Discovery and Development, Kafrelsheikh University, Kafrelsheikh, Egypt; d Department of Chemistry, University of Cambridge, Cambridge, UK; e Department of Chemistry, College of Science, Princess Nourah bint Abdulrahman University, Riyadh, Saudi Arabia; f Department of Pharmaceutical Sciences, College of Pharmacy, AlMaarefa University, Riyadh, Saudi Arabia; g Department of Pharmaceutics, Faculty of Pharmacy, Umm Al-Qura University, Makkah, Saudi Arabia; h Department of Applied Organic Chemistry, National Research Center, Dokki, Egypt; i Department of Pharmaceutical Chemistry, Faculty of Pharmacy, Cairo University, Cairo, Egypt; j Department of Pharmaceutical Chemistry, School of Pharmacy, NewGiza University (NGU), Cairo, Egypt

**Keywords:** Kinase inhibitors, pancreatic cancer, molecular docking, benzofuran synthesis, apoptotic agents

## Abstract

In the current work, a hybridisation strategy was adopted between the privileged building blocks, benzofuran and piperazine, with the aim of designing novel CDK2 type II inhibitors. The hybrid structures were linked to different aromatic semicarbazide, thiosemicarbazide, or acylhydrazone tails to anchor the designed inhibitors onto the CDK2 kinase domain. The designed compounds showed promising CDK2 inhibitory activity. Compounds **9h**, **11d**, **11e** and **13c** showed potent inhibitory activity (IC_50_ of 40.91, 41.70, 46.88, and 52.63 nM, respectively) compared to staurosporine (IC_50_ of 56.76 nM). Moreover, benzofurans **9e**, **9h**, **11d**, and **13b** showed promising antiproliferative activities towards different cancer cell lines, and non-significant cytotoxicity on normal lung fibroblasts MRC-5 cell line. Furthermore, a cell cycle analysis as well as Annexin V-FITC apoptosis assay on Panc-1 cell line were performed. Molecular docking simulations were performed to explore the ability of target benzofurans to adopt the common binding pattern of CDK2 type II inhibitors.

## 1. Introduction

Cancer is a group of diseases that is featured by the uncontrolled growth, proliferation and spreading (metastasis) of atypical malignant cells, it is the second cause of death in the US following heart diseases and according to the 2020 ACS cancer facts and statistics report, it is assessed that the new cancer cases have been diagnosed in 2020 in the US are more than 1.8 million cases^1^.

Protein kinases (PKs) represent an important protein family in human genome (518 proteins). It regulates key cellular functions like cell growth, proliferation, and apoptosis through the coordination of cellular functions and signals^2–6^. PKs catalyse the transport of the terminal phosphate motif in an ATP molecule onto a hydroxyl functionality in a substrate protein (phosphorylation) exerting their cellular signalling modulation^2^. PKs are classified according to the phosphorylated amino acid in the substrate protein into tyrosine and serine/threonine kinases^7^^,^^8^. PKs are tightly regulated, and their dysregulation results in several diseases such as cancer, neurodegenerative disorders like Alzheimer’s disease, and autoimmune disorders like rheumatoid arthritis. Thus PK inhibitors are a promising tool in curbing the PKs dysregulation in such disorders^9–12^. In oncotherapy, protein kinases inhibitors constitute a main continuously growing category of targeted chemotherapies that is free of the traditional cancer chemotherapy common side actions as they target the signalling pathways and microenvironment of the cancer cells with minor adverse actions on normal mammalian cells^13–16^.

Cyclin dependent kinases (CDKs) represent a conserved group of serine/threonine PKs that are involved in several key cellular processes. CDK1, CDK2, CDK4 and CDK6 subtypes are accountable for driving orderly the cell cycle in the different phases, cell differentiation and apoptosis. Furthermore, they have a crucial role in evoking cancer cells’ uncontrolled proliferation^17–19^. Pan-CDK inhibitors showed weaknesses as anticancer agents with disappointing results in clinical trials suggesting that improving selectivity towards a certain CDK is a promising strategy for developing CDK inhibitors as potential cancer chemotherapy^20^.

**Figure 1.**
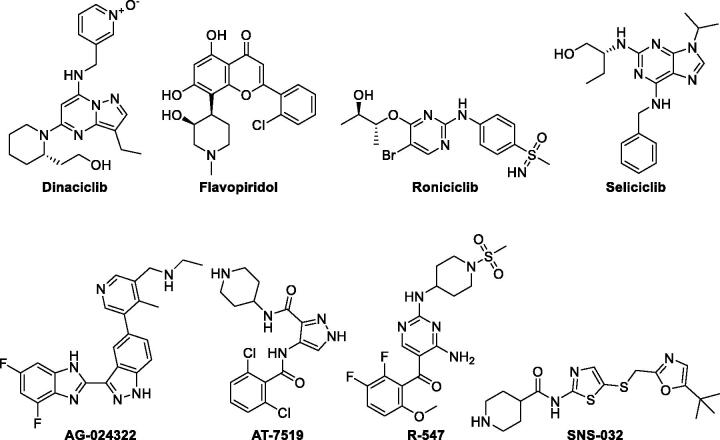
CDK2 inhibitors in clinical trials.

Special attention has been given to CDK2 subtype as a potential target for the management and treatment of different tumours based on its key role in many cellular processes upon binding to its cyclin A or E partners; such that its complex with cyclin E regulates S phase entry and progression, whereas its binding with cyclin A warrants continuous DNA replication as well as G2/M phase transition^20^^,^^21^. Moreover, CDK2 has a significantly broad substrate profile being responsible for the phosphorylation of many proteins which are involved in cell cycle progression, DNA replication, histone synthesis, and centrosome duplication^20^. Furthermore, various cancer types showed dysregulations in CDK2 and/or its cyclin cognates such as breast cancer, endometrial, ovarian, thyroid, lung, hepatocellular carcinomas, melanoma, lymphoma, osteosarcoma, prostate, colorectal, pancreatic cancers, neuroblastoma, and BRCA deficient cancers^20–31^. Several CDK2 inhibitors showed a promising anticancer activity, therefore, some CDK2 inhibitors were advanced through clinical trials (Figure 1)^21^^,^^27^^,^^28^^,^^32–34^. All these facts confirm the significance of CDK2 kinase as an efficient therapeutic target for cancer management.

As a protein kinase, CDK2 can swing between active and inactive conformations due to the movement of the conserved tripeptide motif Asp-Phe-Gly (DFG) and the outward flip of the DFG loop results in the DFG-out inactive conformation^4^^,^^35^. Accordingly, the majority of PK inhibitors can be broadly classified into type I inhibitors interacting with the DFG-in active conformation and type II inhibitors interacting with the DFG-out inactive conformation. In the general definition for the type II inhibitor pharmacophore, type II inhibitors are anchored into DFG-out conformation through hydrogen bonding in the gate area with the conserved N-lobe αC helix glutamate sidechain carboxylate and the DFG aspartate backbone NH. Moreover, type II inhibitors extend into the hydrophobic allosteric back pocket, resulted from the DFG-out flip, interacting through hydrophobic interactions with the hydrophobic side chains of the lining residues of the back pocket^4^^,^^36–38^.

Compound **I** is a model CDK2 type II inhibitor interacting with the DFG-out inactive conformation. It interacts in the gate area *via* hydrogen bond interactions by its diaryl urea moiety with the αC helix Glu51 sidechain carboxylate and the DFG Asp145 backbone NH. This orientation directs the aminopyrimidine moiety from one side towards the hinge region interacting *via* H-bonding with Leu83 and from the other side, it directs the trifluoromethyl phenyl moiety into the hydrophobic allosteric back pocket interacting through hydrophobic interactions with lining amino acids. Moreover, it extends the piperazinyl moiety towards the bulk solvent interacting through hydrogen bonding with Leu124 and His125^35^.


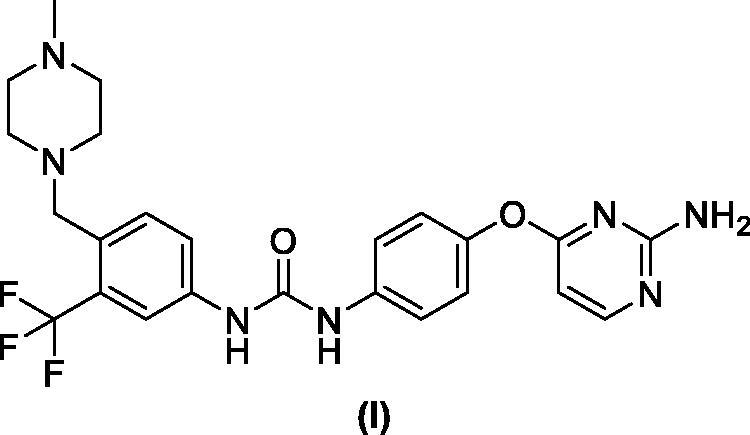


Using type II inhibitors for CDK2 inhibition has several advantages over type I inhibitors, such that their extension towards the less conservative hydrophobic allosteric back pocket that enhances their affinity and selectivity^10^^,^^37^^,^^39^. Moreover, they not only inhibit CDK2 kinase activity through ATP competition but also, they lock it in an inactive conformation which is not competent for cyclin binding, preventing the activation of CDK2^35^. Furthermore, they possess slow off-rates (∼10 fold slower) and prolonged target engagement and so longer residence time and duration of suppression^4^^,^^35^^,^^40^.

Benzofuran is a privileged scaffold exhibiting numerous biological activities amongst them are analgesic, antifungal, antibacterial, anti-hyperlipidemic, antihyperglycemic, anti-inflammatory, antioxidant, antipyretic, antiviral, as well as antitumor actions^41–44^. Benzofuran-based derivatives can exert the antitumor actions *via* several mechanisms such as the inhibition of farnesyltransferase, oestrogen receptor, human peptide deformylase, tubulin polymerisation, angiogenesis, or carbonic anhydrases^41^^,^^45^. In addition, many benzofuran-based small molecules mediate their anticancer actions through protein kinases inhibition such as GSK-3β^44^^,^^46^, mTOR signalling^47^^,^^48^, Pim-1^49^, Src kinase^50^, as well as CDK2^44^. Compounds **II** showed a potent CDK2 inhibitory activity with IC_50_ of 52.75 nM inducing cell cycle arrest of MCF-7 breast cancer cells wihin the G2/M phase causing cell apoptosis^44^.


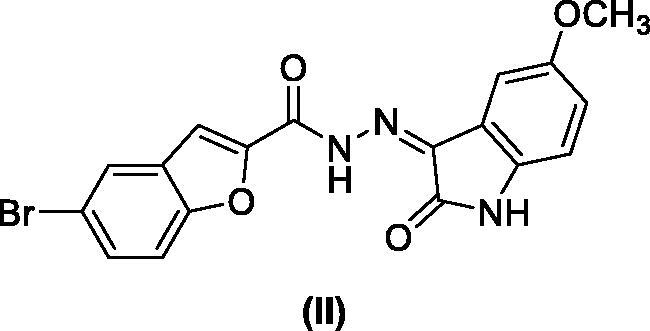


**Figure 2.**
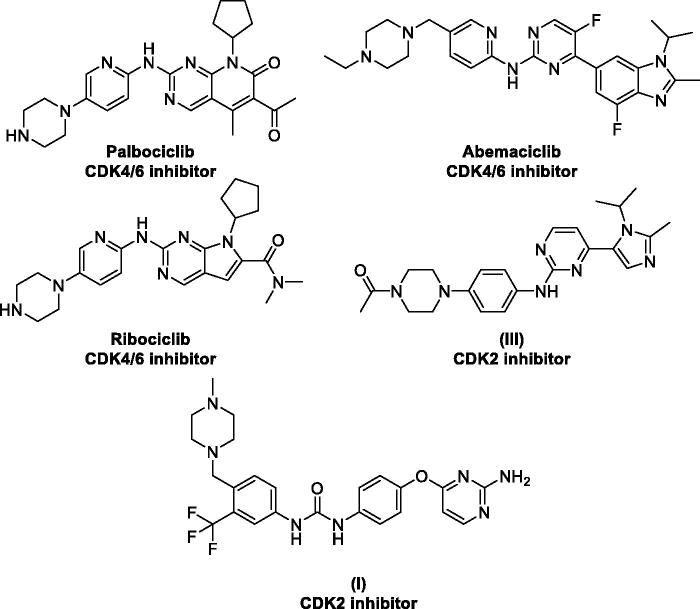
Representative piperazine derivatives with CDKs inhibitory activity.

Piperazine is another privilege heterocycle that represents a key moiety in several bioactive small molecules that show a broad spectrum of biological activities^51–56^. Piperazine derivatives show antibacterial, anticonvulsant, antidepressant, antifungal, antimalarial, antimycobacterial, anthelmintic, antiviral, cardio protecting, as well as anticancer activities^55^^,^^56^. They were reported to exert their anticancer activities through diverse mechanisms such as production of reactive oxygen species (ROS) and decreasing of c-FLIP^57^, Lysine-specific histone demethylase 1 A (LSD1) inhibition^58^, ROS-mediated RhoB expression through the upregulation of c-Abl, as well as the activation of p38 MAPK/ATF-2^59^, dual inhibition of REV-ERB*β* and autophagy^60^, ribonucleotide reductase (RR) and HDAC inhibition^61^, depolarisation of mitochondrial membrane potential^62^, chemokine receptor (CCR5) antagonism^63^, DNA intercalation^64^, smoothened antagonism^65^^,^^66^, stearoyl-CoA desaturase inhibition^67^, Bcl-2/Bcl-xl inhibition^68^, and PARP1 inhibition^69^. In addition, one of the main mechanisms for piperazine derivatives anticancer activity is kinase inhibition such as anaplastic lymphoma kinase (ALK) ^70^, BCR–Abl^71^, RET kinase^72^, epidermal growth factor receptor (EGFR) ^73^, vascular epithelial growth factor receptor 2 (VEGFR-2) ^74^, focal adhesion kinase (FAK) ^75^, as well as CDKs family, CDK2^35^^,^^51^^,^^76^, CDK4/6^77^, and CDK9^76^ (Figure 2).

**Figure 3.**
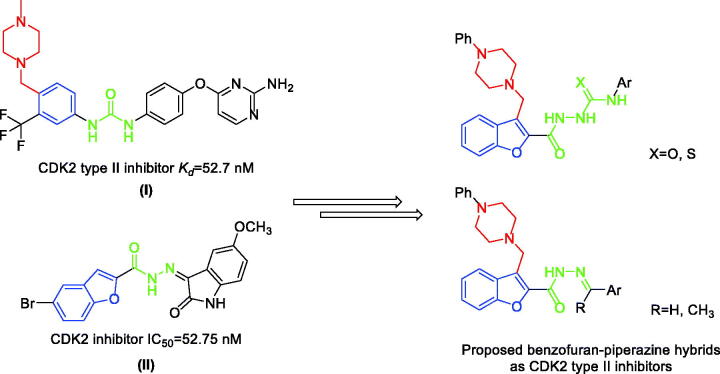
The design strategy of the novel benzofuran-piperazine hybrids as CDK2 type II inhibitors.

Despite of the forementioned type II advantages, most of the CDK2 inhibitors discovery campaigns focus on designing type I inhibitors. In the current work, a hybridisation strategy was adopted between the privileged building blocks, benzofuran and piperazine, with the aim of designing novel CDK2 type II inhibitors. The hybrid structures were linked to different aromatic thiosemicarbazide, semicarbazide, or acylhydrazone tails to anchor the designed inhibitors onto the CDK2 DFG-out conformation in the gate area through hydrogen bonding to the conserved αC glutamate (αC-Glu51) and the aspartate of the DFG motif (DFG-Asp154) (Figure 3).

Different aromatic moieties were used in the tail part to study their effect on the binding affinities and the kinase inhibitory activities. The newly synthesised hybrids were then tested for their CDK2 inhibitory activity and cytotoxic activity using pancreatic cancer (Panc-1), breast cancer (MCF-7), and lung carcinoma (A549) cell lines. Moreover, their safety was assessed using human lung fibroblast normal cell line (MRC-5). To confirm their mode of action, the effect of representative compounds **9h** and **11d** on cell cycle progression were also investigated. Finally, molecular docking simulations were carried out to confirm their binding mode and to rationalise their structure activity relationships (SARs).

## 2. Results and discussion

### 2.1. Chemistry

**Scheme 1.**
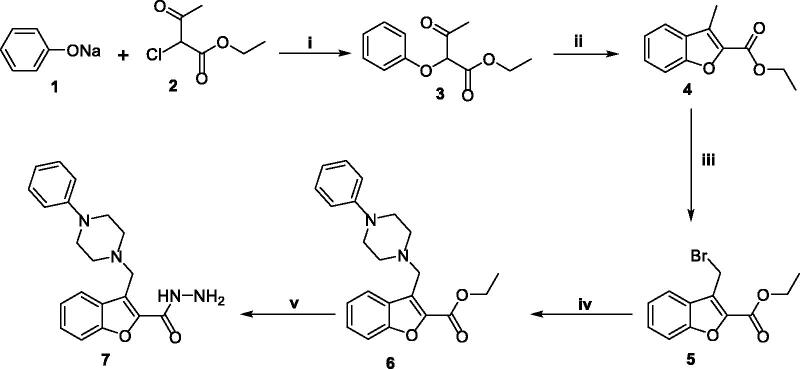
*Reagent and conditions:* (**i**) Toluene, reflux 4 h; (**ii**) Sulphuric acid, stirring 2 h (0–5 °C); (**iii**) NBS, CCl_4_, reflux 3 h; (**iv**) *N*-phenylpiperazine, acetone, K_2_CO_3_, KI, reflux 4 h; (**v**) 99% NH_2_NH_2_·H_2_O, ethyl alcohol, reflux 4 h.

The retrosynthetic analyses for all target 3-(piperazinylmethyl)benzofurans (**9**, **11**, **13**, **15** and **17**) have led to a consensus of utilising the hydrazide **7** as a building block. An appropriate synthesis of the hydrazide **7** including a benzofuran cyclisation step is outlined in Scheme 1. Firstly, an O-C2 bond was formed in the newly emerging furan ring *via* utilising an S_N_2 reaction of ethyl *α*-aceto-*α*-chloroacetate **2** with sodium phenoxide nucleophile. Then, a C3-C4 bond was formed to furnish the desired benzo[*b*]furan derivative **4** *via* a dehydration step carried out on the α-phenoxy-β-ketoester **3** using sulphuric acid at mild conditions. After that, the 3-methyl group of compound **4** was activated for further nucleophilic substitution reaction by introducing bromine atom using *N*-bromosuccinimide reagent to afford the 3-(bromomethyl)benzofuran derivative **5**. Compound **5** was then subjected to an S_N_2 reaction with *N*-phenylpiperazine in refluxing acetone using anhydrous potassium carbonate as a base to afford the *N*-phenylpiperazinyl substituted benzofuranyl ester **6**. It’s noteworthy mentioning the catalytic role of potassium iodide, acting as a nucleophilic assistant in the S_N_2 mechanism which afforded a noticeable increase in the experimental reaction yield. Finally, the target hydrazide intermediate **7** was obtained *via* hydrazinolysis of the corresponding ester precursor **6** in refluxing ethanol.

**Scheme 2.**
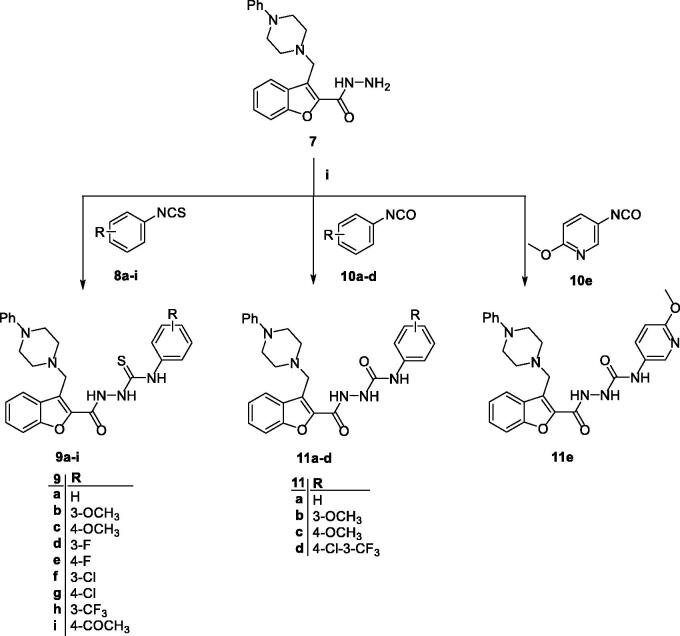
*Reagent and conditions:* (**i**) Toluene, reflux 7 h.

**Scheme 3.**
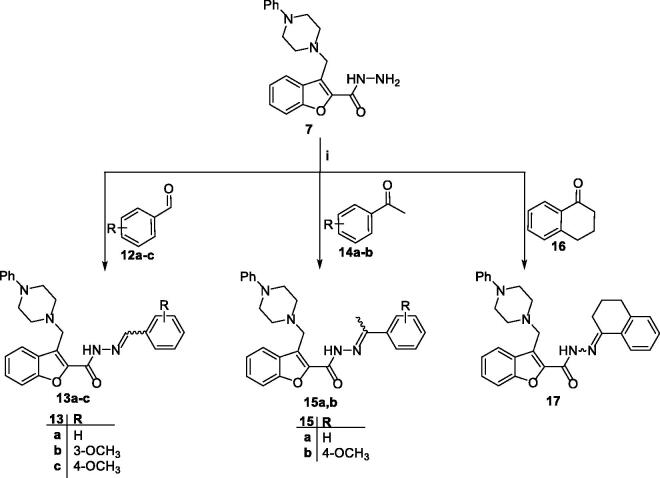
*Reagent and conditions:* (**i**) Absolute ethyl alcohol, acetic acid (cat.), reflux 11 h.

Regarding the synthesis of target benzofurans **9**, **11**, **12**, **14** and **16**, the hydrazide **7** was coupled with isothiocyanates **8a-i** and isocyanates **10a-e** *via* nucleophilic addition reaction in refluxing toluene to afford the desired thiosemicarbazides **9a-i** and semicarbazides **11a-e**, respectively (Scheme 2). Analogously, the aldohydrazones **13a-c** as well as the ketohydrazones **15a,b** & **17** were prepared by a simple condensation reaction of the hydrazide **7** with the aldehydes **12a-c** and ketones **14a,b** & **16** in refluxing ethanol using weak acid catalysis (Scheme 3).

The structure of intermediates **6**, **7** as well as those of target compounds **9**, **11**, **13**, **15** and **17** were confirmed by ^1^H as well as ^13 ^C NMR spectral analysis (see the NMR charts in the supplementary material). All of the relevant spectral data of these compounds shared some common NMR signals. These included the following ^1^H NMR signals: Ar˗H of C4, C7, C6 and C5 of benzofuran nucleus at *δ*_ppm_ around 8 (d), 7.7 (d), 7.5 (t) and 7.4 (t), respectively; Ar˗H of the phenyl ring at *N*4 piperazine at *δ*_ppm_ around 7.2, 6.9 and 6.8; two sets of aliphatic Hs of piperazine ring at δ_ppm_ around 3.1 (4H) and 2.6 (4H); methylene group exocyclic to C3 benzofuran at δ_ppm_ around 4.1 (2H, s). Moreover, compound **7** was confirmed by NH hydrazide signal at δ_ppm_ 10.68; compound series **9a-i** and **11a-e** by the three NH (thio)semicarbazide singlets at δ_ppm_ around 12.2, 9.9 and 9.8; aldohydfrazone series **13a-c** by the characteristic H-C=N hydrazone signal at *δ*_ppm_ around 8.5; compounds **15a,b** by the characteristic CH_3_-C=N at *δ*_ppm_ around 2.5 (3H, s); and finally compound **17** by the characteristic aliphatic H signals of 3,4-dihydronaphthalen-1(*2H*)-ylidene moiety at *δ*_ppm_ 1.99–1.80 (m, 2 H), 2.92–2.78 (m, 4 H) in addition to the relevant aromatic ones at δ_ppm_ 8.04 (d, *J =* 7.8 Hz, 1H, Ar˗H at C8), 7.40–7.28 (m, overlapped signals including Ar˗H at C6,7) and 7.28–7.18 (m, overlapped signals including Ar˗H at C5). Finally, the Ar˗H signals corresponding to the additional (substituted)phenyl ring hydrogens at N4 (thio)semicarbazide in benzofurans **9a-i**, **11a-e** as well as those Ar˗Hs of the benzylidene moiety at N1 hydrazide in benzofurans **13a-c**, **15a,b** were characteristic for structure confirmation of these compounds (See the Experimental section for details).

Regarding ^13 ^C NMR spectral analysis, structures of all studied compounds were in agreement with the expected ones as confirmed by the number of ^13 ^C NMR signals corresponding to aliphatic, aromatic, carbonyl and/or imine carbons (see the experimental section and the supplementary material).

### 2.2. Biological evaluation

To validate the design strategy, assessments of the CDK2 inhibitory activity as well as the anticancer activities for the newly synthesised piperazine tethered benzofuran derivatives were carried out. The cytotoxic activity was evaluated against pancreatic cancer (Panc-1), breast cancer (MCF-7), and lung carcinoma (A549) cell lines. Moreover, the effect of the most promising compounds on the cell cycle progression was also investigated in order to confirm their mode of action.

#### 2.2.1. Cdk2 inhibitory activity

To validate the design strategy in this work which aim to the design of potent CDK2 inhibitors, the CDK2 inhibitory activity of all the newly synthesised piperazine tethered benzofurans **9a-i**, **11a-e**, **13a-c**, **15a,b** and **17** was evaluated against stuarosporine as a reference standard and IC_50_ were calculated in nM.

The thiosemicarbazide derivatives **9a-i** showed CDK2 inhibitory concentration (IC_50_) range of 40.91–322.1 nM. The *m*-trifluoromethyl derivative **9h** showed potent inhibitory activity relative to that of staurosporine (IC_50_ = 40.91 vs 56.76 nM, respectively). Compounds **9b**, **9d**, **9e**, **9 g** and **9i** showed comparable CDK2 inhibitory activity to that of staurosporine (the used reference standard) with IC_50_ less than 100 nM. Compounds **9a**, **9c** and **9f** showed weak inhibitory activity compared to that of staurosporine (IC_50_ = 322.10, 214.00 and 119.00 nM, respectively, vs 56.76 nM) (Table 1).

**Table 1.** Inhibitory activities of the newly synthesised benzofuran derivatives **9a-i**, **11a-e**, **13a-c**, **15a-b** and **17** against CDK2

Comp.	**IC_50_ (nM)** ^a^
	CDK2
**9a**	322.10 **±** 16
**9b**	84.92 **±** 4.30
**9c**	214.00 **±** 11
**9d**	77.67 **±** 41
**9e**	64.97 **±** 3.30
**9f**	119.00 **±** 6.10
**9g**	69.80 **±** 3.60
**9h**	**40.91 ± 2.10**
**9i**	89.44 **±** 4.62
**11a**	137.20 **±** 7.00
**11b**	129.30 **±** 6.60
**11c**	182.50 **±** 9.30
**11d**	**41.70 ± 2.10**
**11e**	**46.88 ± 2.40**
**13a**	151.70 **±** 7.70
**13b**	65.63 **±** 3.31
**13c**	**52.63 ± 2.70**
**15a**	333.10 ± 17
**15b**	127.10 ± 6.50
**17**	65.58 ± 3.30
**Staurosporine**	56.76 ± 2.90

**^a^**IC_50_ values are the mean ± SD of three separate experiments.

Bold values indicate the best results.

The semicarbazide derivative **11a-e** showed CDK2 inhibitory concentration (IC_50_) range of 41.70–182.5 nM. The 4-Cl-3-CF_3_-phenyl derivative **11d** and the pyridyl derivative **11e** showed potent inhibitory activity relative to that of staurosporine (IC_50_ = 41.7 and 46.88 nM, respectively, vs 56.76 nM). Whereas compounds **11a-c** showed weaker CDK2 inhibitory activity relative to that of staurosporine (IC_50_ of 137.20, 129.30 and 182.50 nM, respectively, vs 56.76 nM) (Table 1).

The acylhydrazone counterparts **13a-c**, **15a,b** and **17** showed CDK2 inhibitory concentration (IC_50_) range of 52.63–333.17 nM. The *p*-methoxyphenyl derivative **13c** showed slightly potent inhibitory activity relative to that of staurosporine (IC_50_ = 52.63 vs 56.76 nM, respectively). Compounds **13b** and **17** showed comparable CDK2 inhibitory impact to that of staurosporine with IC_50_ of 65.63 nM and 65.58 nM, respectively. The unsubstituted phenyl derivative **13a** and the methyl acylhydrazone derivatives **15a** and **15b** showed weak inhibitory activity compared to that of staurosporine (IC_50_ = 151.70 nM, 333.10 nM and 127.10 nM, respectively, vs 56.76 nM) (Table 1).

These results highlighted that, as planned, the designed compounds showed promising CDK2 inhibitory activity with compounds **9h**, **11d**, **11e** and **13c** showing potent inhibitory activity compared to staurosporine (the used reference standard), Table 1.

#### 2.2.2. *In vitro* anti-proliferative activity

To study the antiproliferative activity of the newly synthesised piperazine tethered benzofuran derivatives **9a-i**, **11a-e**, **13a-c**, **15a,b** and **17**, their cytotoxic activity were evaluated using pancreatic cancer (Panc-1), breast cancer (MCF-7), and lung carcinoma (A549) cell lines which are known to possess CDK2 dysregulation^20–31^.

The thiosemicarbazide derivatives **9a-i** show comparable cytotoxicity on the tested cell lines with IC_50_ ranges of 0.94–125.85 µM, 2.92–127.65 µM and 1.71–126.89 µM against Panc-1, MCF-7, and A549 cell lines, respectively. The *m*-trifluoromethyl derivative **9h** displayed the most potent activity in this series with IC_50_ of 0.94, 2.92 and 1.71 µM, and more potent than that of the used reference standard (cisplatin) which showed IC_50_ of 6.98, 5.45 and 6.72 µM against Panc-1, MCF-7, and A549 cell lines, respectively. Whereas the p-fluoro derivative **9e** was the second most potent in this series with IC_50_ of 3.29, 5.89 and 5.24 µM against Panc-1, MCF-7, and A549 cell lines, respectively, which is also more potent than that of cisplatin (Table 2).

**Table 2.** Anti-proliferative activities of hybrids **9a-i**, **11a-e**, **13a-c**, **15a,b** and **17** against Panc-1, MCF-7 and A-549 cancer cell lines.

Comp.	IC_50_ (µM)^a^		
	Panc-1	MCF-7	A-549
**9a**	86.92 **±** 4.17	118.49 **±** 6.02	85.47 **±** 6.21
**9b**	61.88 **±** 3.48	98.49 **±** 4.47	80.44 **±** 4.87
**9c**	103.13 **±** 6.21	86.63 **±** 5.28	72.19 **±** 5.36
**9d**	27.19 **±** 1.87	53.89 **±** 3.57	40.39 **±** 2.74
**9e**	3.29 **±** 0.20	5.89 **±** 0.43	5.24 **±** 0.40
**9f**	125.85 **±** 7.06	127.41 **±** 7.56	126.89 **±** 7.61
**9g**	121.69 **±** 6.34	120.65 **±** 8.39	110.77 **±** 6.38
**9h**	0.94 **±** 0.10	2.92 **±** 0.17	1.71 **±** 0.12
**9i**	19.15 **±** 1.27	27.33 **±** 2.05	29.23 **±** 2.06
**11a**	56.82 **±** 2.94	55.88 **±** 3.18	49.30 **±** 3.27
**11b**	10.19 **±** 0.86	15.19 **±** 1.34	13.84 **±** 1.04
**11c**	61.45 **±** 4.52	76.93 **±** 5.21	56.95 **±** 2.33
**11d**	2.22 **±** 0.13	5.57 **±** 0.47	2.99 **±** 0.21
**11e**	54.56 **±** 4.22	48.70 **±** 3.54	44.75 **±** 4.62
**13a**	169.27 **±** 9.41	118.40 **±** 8.39	108.76 **±** 7.59
**13b**	1.04 **±** 0.08	2.98 **±** 0.18	1.71 **±** 0.10
**13c**	4.47 **±** 0.29	13.64 **±** 0.94	14.38 **±** 1.02
**15a**	120.12 **±** 5.77	122.98 **±** 6.82	98.38 **±** 5.61
**15b**	25.38 **±** 1.70	44.35 **±** 2.96	28.47 **±** 1.92
**17**	80.40 **±** 4.83	82.80 **±** 5.43	58.87 **±** 1.88
Cisplatin	6.98 **±** 0.35	5.45 **±** 0.40	6.72 **±** 0.37

**^a^**IC_50_ values are the mean of 3 separate experiments.

Likewise, the semicarbazide derivatives **11a-e** show comparable cytotoxicity on the tested cell lines with IC_50_ ranges of 2.22–61.45 µM, 5.57–76.93 µM and 2.99–56.95 µM against Panc-1, MCF-7, and A549 cells, respectively. The 4-Cl-3-CF_3_-phenyl derivative **11d** showed the most potent cytotoxic activity in this series with IC_50_ of 2.22, 5.57 and 2.99 µM against Panc-1, MCF-7, and A549 cell lines, respectively, that is also more potent than that of cisplatin (IC_50_ of 6.98, 5.45 and 6.72 µM, respectively) (Table 2).

The acylhydrazone derivatives **13a-c**, **15a,b** and **17** exerted comparable cytotoxicity towards the tested cell lines displaying IC_50_ ranges of 1.04–169.27 µM, 2.98–122.98 µM and 1.71–108.76 µM against Panc-1, MCF-7, and A549 cells. The *m*-methoxyphenyl derivative **13b** showed the most potent cytotoxic activity in this series with IC_50_ of 1.04, 2.98 and 1.71 µM against Panc-1, MCF-7, and A549 cells, respectively, that is also more potent than that of cisplatin (IC_50_ of 6.98, 5.45 and 6.72 µM, respectively) (Table 2). The *p*-methoxyphenyl derivative **13c** showed more potent cytotoxic activity against Panc-1 cell line than that of cisplatin (IC_50_ of 4.47 vs 6.98 µM, respectively) (Table 2).

These results show that in the different series, each compound has comparable cytotoxic activities on the tested cancer cell lines. These results show also that compounds **9e**, **9h**, **11d**, and **13b** are the most potent antiproliferative compounds in agreement with their potent CDK2 inhibitory activity (Tables 1 and 2). Despite of its potent CDK2 inhibitory activity, compound **11e** showed moderate antiproliferative activity which could be rationalised to its poor cellular intake.

#### 2.2.3. *In vitro* normal cell cytotoxicity

To test the safety of the newly synthesised compounds and their selectivity towards cancer cells, their cytotoxic activity was tested on non-tumorigenic human lung fibroblast MRC-5 cell line. All the tested compounds showed a high IC_50_ on normal human lung fibroblast relative to their IC_50_ on cancerous lung cell line A-549 with selectivity index (SI) higher than 3 folds.

The most potent compounds **9e**, **9h**, **11d**, and **13b** showed IC_50_ on MRC-5 of 52.00, 27.70, 74.00 and 18.10 µM, respectively, which represent SI of 9.92, 16.20, 24.75 and 10.58, respectively (Table 3). These results indicate that these benzofuran derivatives are not only with promising CDK2 inhibitory effect and cytotoxicity but also with selectivity towards cancerous cells and so tolerable and safe effect on normal cells.

**Table 3.** Inhibitory activities against non-tumorigenic human lung fibroblast MRC-5 cell line

Comp.	IC_50_ (μM)^a^	SI^b^
	MRC-5	
**9a**	406 **±** 21	4.75
**9b**	289 **±** 19	3.59
**9c**	261 **±** 20	3.62
**9d**	166 **±** 14	4.11
**9e**	52 **±** 3	9.92
**9f**	391 **±** 25	3.08
**9g**	342 **±** 30	3.09
**9h**	27.7 **±** 1.8	16.20
**9i**	142 **±** 11	4.86
**11a**	178 **±** 13	3.61
**11b**	121 **±** 10	8.74
**11c**	246 **±** 13	4.32
**11d**	74 **±** 5	24.75
**11e**	244 **±** 18	5.45
**13a**	>500	>4.60
**13 b**	18.1 **±** 1.3	10.58
**13c**	121 **±** 9	8.41
**15a**	452 **±** 28	4.59
**15b**	160 **±** 10	5.62
**17**	284 **±** 15	4.82

**^a^**IC_50_ values are the mean of three separate experiments.

^b^SI safety index calculated as IC_50_ in normal lung cell/IC_50_ in cancerous lung cells A-549.

#### 2.2.4. Cell cycle analysis

As previously mentioned, CDK2/cyclin E complexation regulates S phase entry and progression, whereas CDK2/cyclin A complexation permits continuous DNA replication and G2/M phase transition and progression^24^^,^^30^^,^^78^^,^^79^. Moreover, a significant CDK2-deficient cells percentage could be arrested in the G2/M phase, in addition, cancerous cells treated with CDK2 inhibitors display an arrest within the G2/M phase^22^^,^^80^.

**Figure 4.**
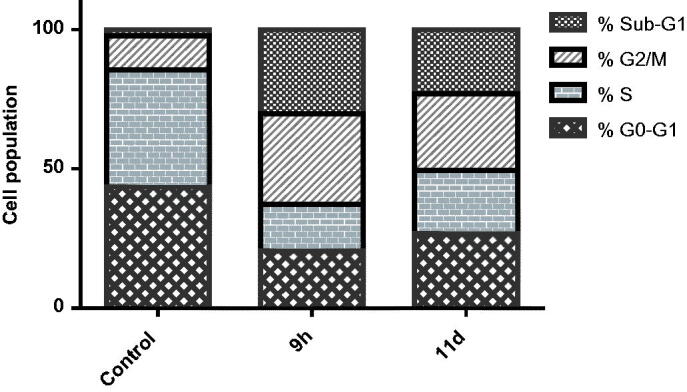
Impact of benzofurans **9 h** and **11d** towards the cell cycle phases of Panc-1 cancer cells.

In this research work, the effect of the two most efficient cell growth inhibitors **9h** and **11d** towards the progression of the cell cycle was investigated aiming to explore the cell cycle phase which could be arrested to confirm the mode of action of the target benzofurans. Panc-1 cancerous cells were incubated with the IC_50_ dose of both benzofurans **9h** and **11d**, and the impact on the cells populations was determined within the different cell phases. Treatment of Panc-1 cells with **9h** and **11d** led to a significant decline in the cells population regarding the G0/G1 and S phases from 43.57% and 41.90%, respectively, in the control to 20.56% and 16.66%, respectively, with compound **9h** and 26.83% and 22.61%, respectively, with compound **11d** (Figure 4). This is accompanied by a significant elevation in the proportion of cells in G2/M phase with compounds **9h** and **11d (**32.46% and 27.52%, respectively) relative to the control (12.17%) with simultaneous increase in the proportion of cells in the sub-G1 phase (30.32% and 23.04%, respectively) in comparison to the control (2.36%). These results indicate that the newly synthesised compounds cause cell cycle arrest in the G2/M phase which is the main criterion of CDK2 inhibitors confirming the mode of action under investigation.

#### 2.2.5. Annexin V-FITC (Anx V) apoptosis assay

Anx V flowcytometry assay is featured as a helpful tool to determine whether cells death is due to the physiological apoptosis or to the non-specific necrosis. In this work, the impact of the most promising benzofurans **9h** and **11d** towards Panc-1 cell apoptosis was investigated using Anx V-propidium iodide dual staining analysis in accordance with the standard protocol^81^.

**Figure 5.**
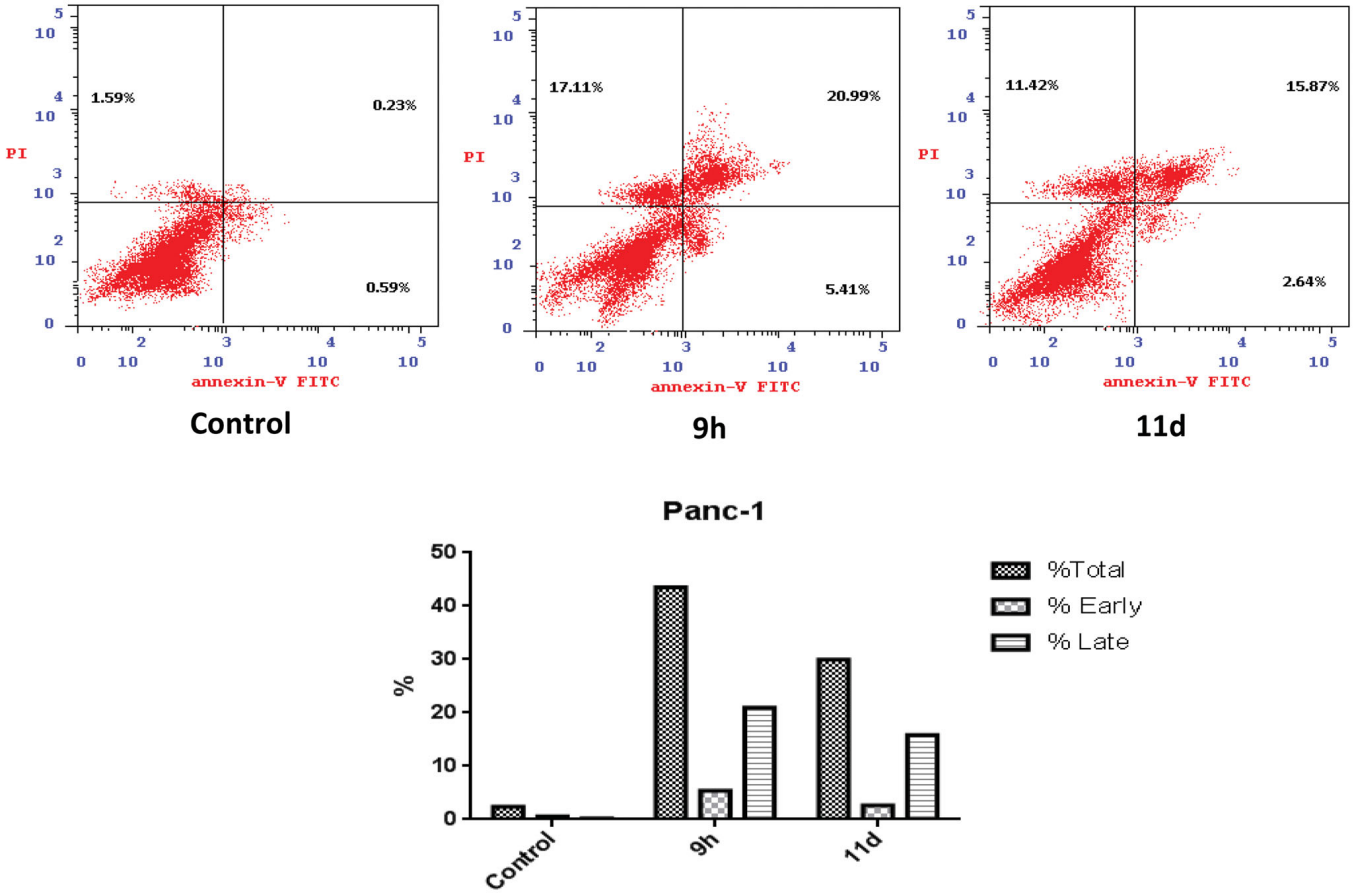
Influence of **9h** and **11d** on the percentage of annexin V-FITC-positive staining in Panc-1 cells.

The Anx V-based flowcytometry assay results (Figure 5) revealed that the total apoptotic cells percentage in Panc-1 cancerous cells increased upon treatment with benzofurans **9h** and **11d** from 2.41% (for the control cells) to 43.51 and 29.93%, respectively, which significantly suggest the apoptotic impact of the target 3-(piperazinylmethyl) benzofuran derivatives. In details, benzofurans **9h** and **11d** were able to elevate the percentages of the early apoptotic phases from 0.59% (for the control cells) to 5.41% and 2.64%, respectively, in addition they increased the percentages of the late apoptotic phase, from 0.32% in control to 20.99% and 15.87%, respectively. This obviously verifies that the anti-proliferative impact of the designed 3-(piperazinylmethyl) benzofuran derivatives is attributable to the physiological apoptosis and not attributable to the non-specific necrosis.

### 2.3. Molecular docking study and SAR

CDK2 is one of the kinases that have a considerable number of structures in the protein data bank (PDB), with more than 200 available crystal structures in the PDB co-crystalized with different inhibitors^30^^,^^35^^,^^82^. Nearly all available crystal structures are for CDK2 protein adopting DFG-in conformation co-crystalized with type I inhibitors accommodated in the hinge region^35^. In this molecular modelling study, one of the unique CDK2 crystal structures adopting DFG-out conformation co-crystalized with type II inhibitor (compound **I**) has been utilised (PDB ID: 5A14)^35^.

**Figure 6.**
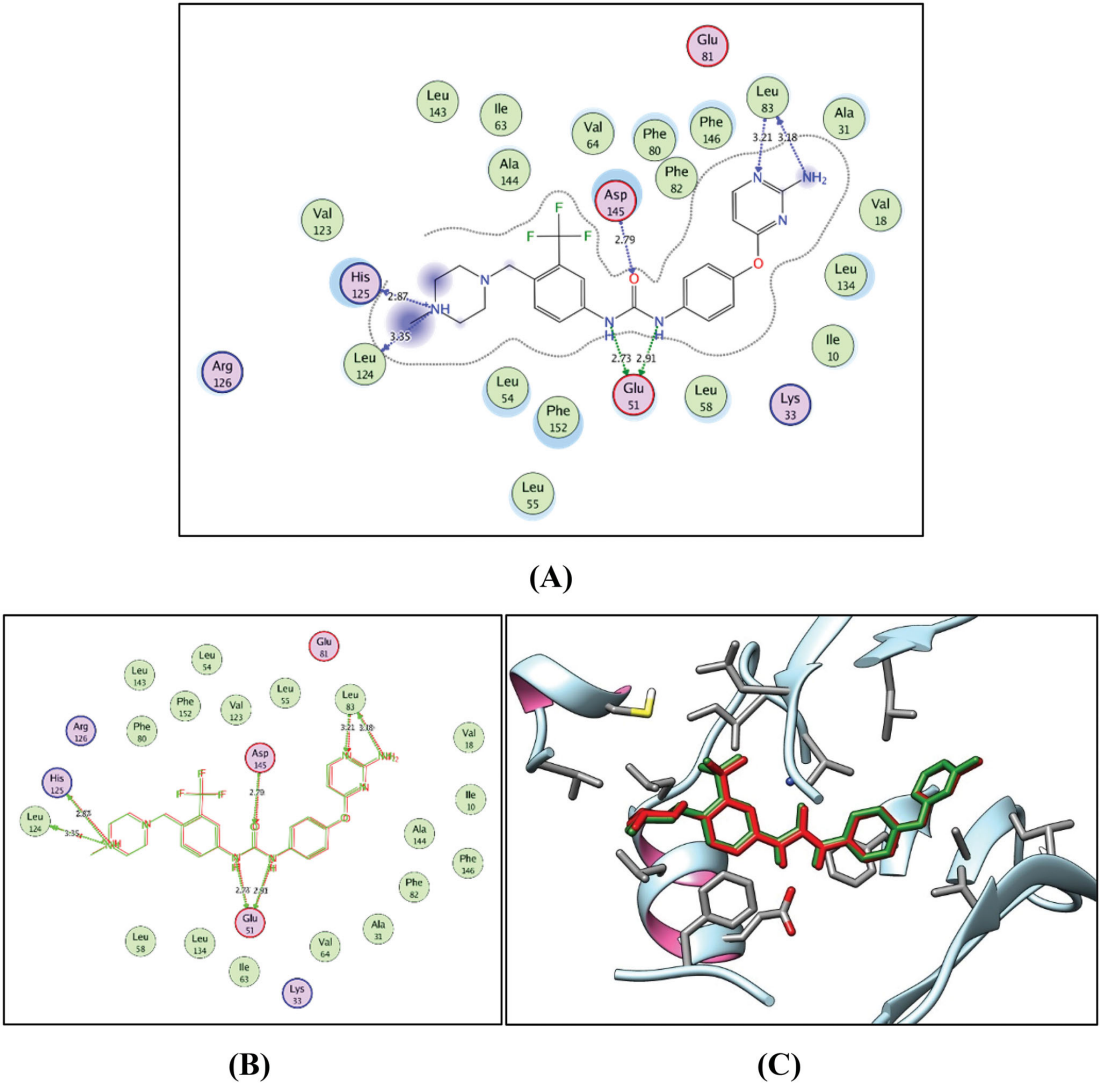
(**A**) 2 D interactions of compound **I** within the CDK2 active site. (**B**) and (**C**) 2 D and 3 D diagrams for the superimposition of the docking pose (green) and the co-crystallised pose (red) for compound **I** within the CDK2 binding site (RMSD = 0.245 Å).

Firstly, self-docking for the co-crystallised ligand (compound **I**) within the CDK2 binding site was carried out to prove the molecular docking setup to be used. Self-docking validation precisely reproduced the binding mode of the co-crystallised ligand highlighting the aptness of the adopted modelling protocol for the intended molecular docking simulations. In addition, it was confirmed through the small RMSD between the docked and the co-crystallised ligand poses of 0.245 Å (*S* = −16.61 kcal/mol), and also by the capability of the accomplished docking pose to regenerate the main binding interactions achieved by the co-crystallised ligand with the key amino acids within the CDK2 active site Glu51, Leu83, Leu124, His125 and Asp145 (Figure 6).

**Figure 7.**
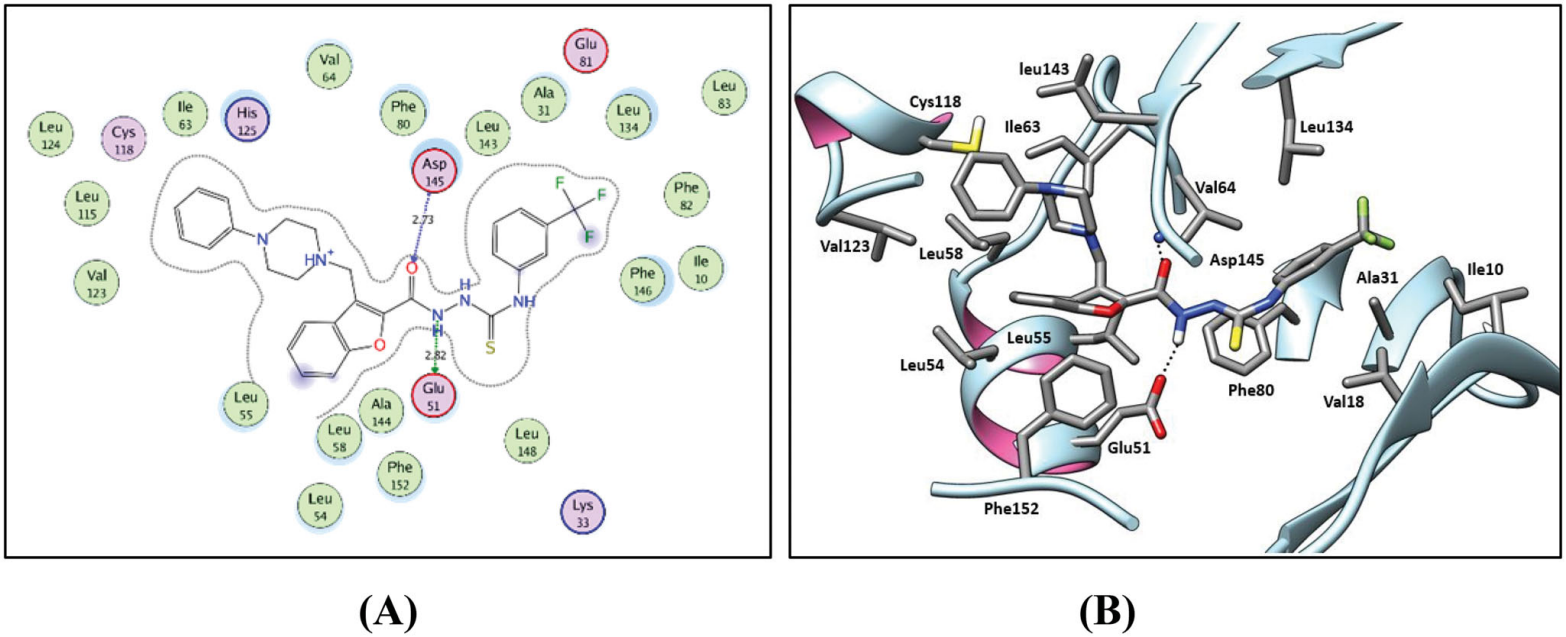
2D diagram (A) and 3 D representation (B) for the thiosemicarbazide derivative **9 h** displaying its interaction within the CDK2 binding site.

**Figure 8.**
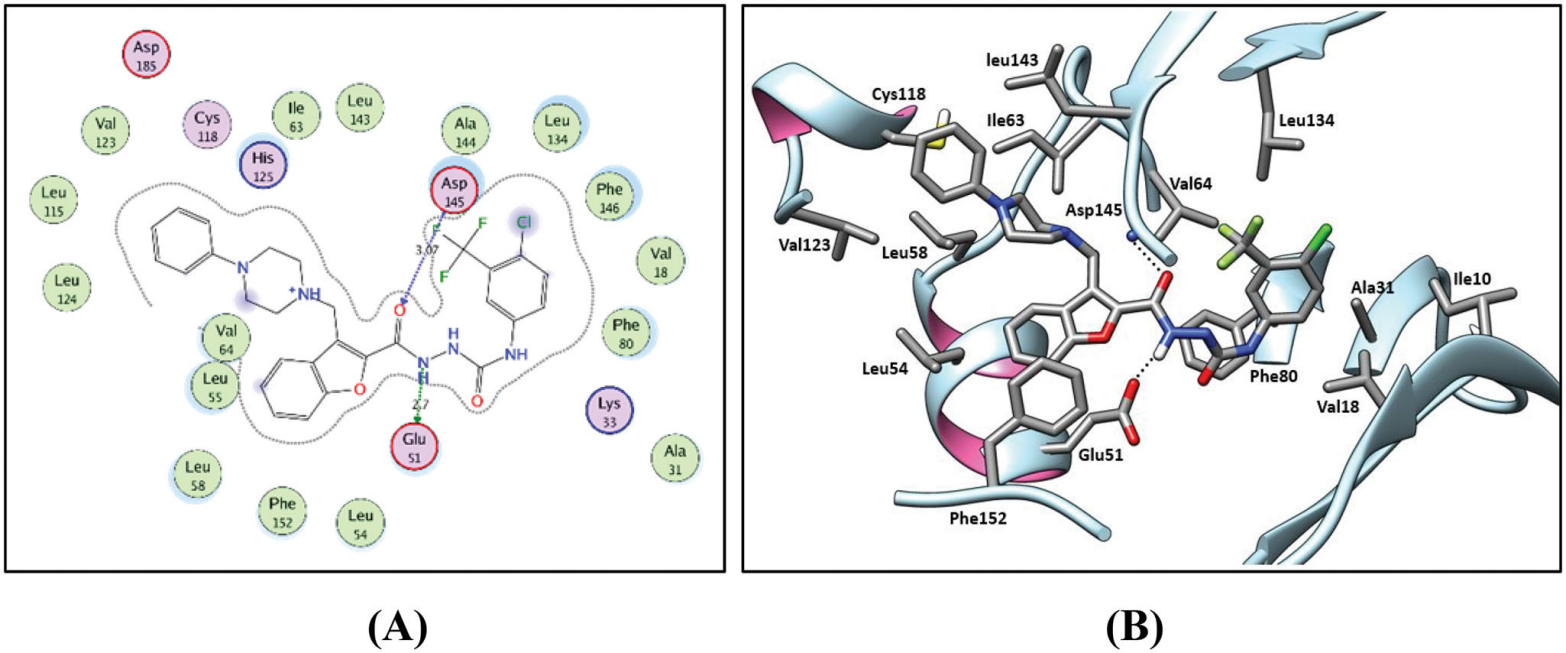
2 D diagram (A) and 3 D representation (B) for the semicarbazide derivative **11d** displaying its interaction within the CDK2 binding site.

**Figure 9.**
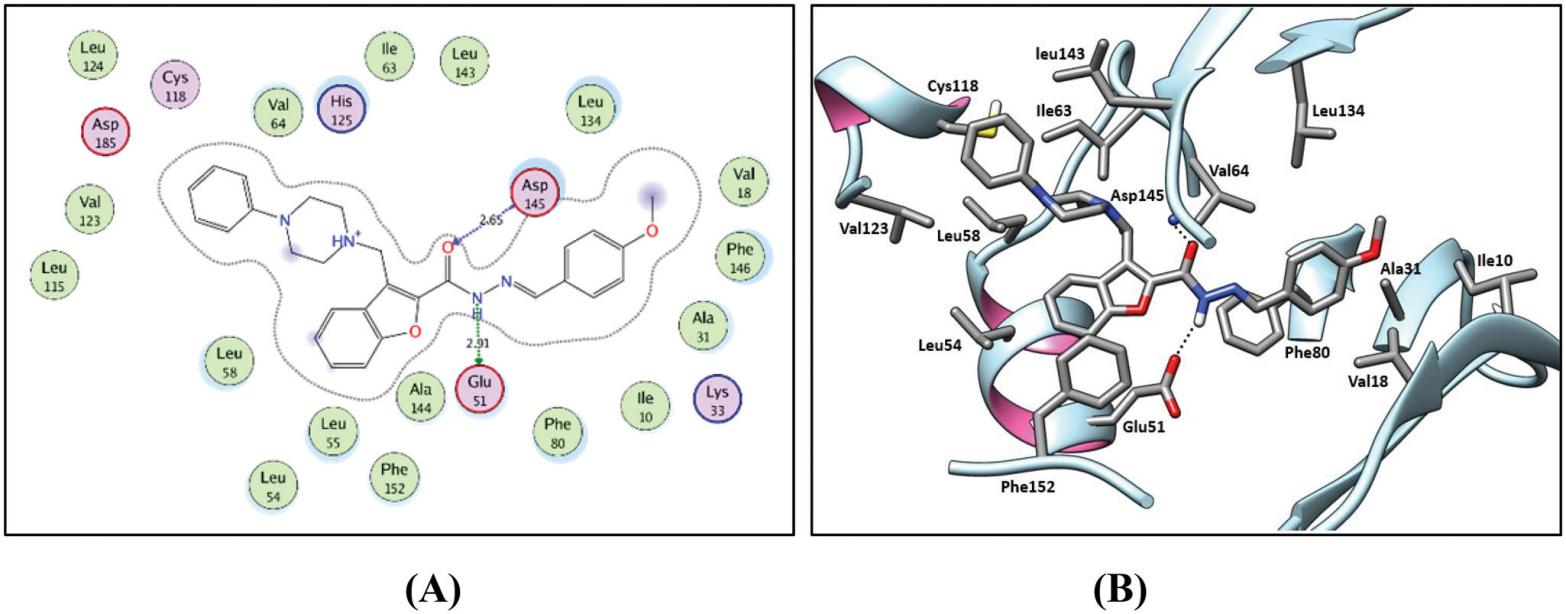
2 D diagram (A) and 3 D representation (B) for the acylhydrazone derivative **13c** displaying its interaction with the CDK2 binding site.

As planned, the designed 3-(piperazinylmethyl)benzofuran derivatives adopted the common binding pattern of type II inhibitors. The thiosemicarbazide, semicarbazide, and acylhyrazone linker in benzofurans **9a-i**, **11a-e**, and **13a-c**, **15a**,**b** and **17**, respectively, are accommodated in the interface between the gate area and the allosteric back pocket in the kinase binding site interacting through hydrogen bonding with N-lobe αC helix Glu51 sidechain carboxylate and the DFG Asp145 backbone NH (Figures 7–9). From one side, this binding mode directs the peripheral (un)substituted phenyl moiety towards the hydrophobic region between the gate area and the hinge region interacting through hydrophobic interactions with the hydrophobic side chains of the surrounding Ala31, Val18, Val64, Phe80, Leu83, Leu134, Phe146 amino acids.

On the other side, the benzofuran ring is directed towards the hydrophobic allosteric back pocket interacting through hydrophobic interactions with the hydrophobic side chains of the amino acids lining the back pocket; Ala144, Phe152, and Leu55, besides, its π-π stacking interaction with Phe152 side chain. In addition, the phenylpiperazine moiety at 3-position of the benzofuran ring is extended towards a hydrophobic region surrounded by the hydrophobic side chains of the amino acids Leu54, Leu58, Ile63, Cys118, Val123, and Leu143.

## 3. Conclusions

In this study, different series of novel CDK2 type II inhibitors were designed *via* the hybridisation strategy between the privileged building blocks, benzofuran and piperazine, that were linked to different aromatic semicarbazide, thiosemicarbazide, or acylhydrazone tails to anchor the designed inhibitors onto the CDK2 kinase domain. The designed compounds showed promising CDK2 inhibitory activity with compounds **9h**, **11d**, **11e** and **13c** showing potent inhibitory activity (IC_50_ of 40.91, 41.70, 46.88, and 52.63 nM, respectively) compared to the used reference standard, staurosporine (IC_50_ of 56.76 nM). Moreover, the designed benzofurans showed promising cytotoxic activities with counterparts **9e**, **9h**, **11d**, and **13b** showing potent antiproliferative activity in agreement with their potent CDK2 inhibitory activity. Compound **9h** showed the most potent cytotoxic activity with IC_50_ of 0.94, 2.92, and 1.71 µM against Panc-1, MCF-7, and A-549 cancer cell lines, respectively, in comparison to the used reference standard, cisplatin, (IC_50_ of 6.98, 5.45, and 6.72 µM, respectively). On the other hand, the most potent compounds **9e**, **9h**, **11d**, and **13b** showed IC_50_ of 52.00, 27.70, 74.00 and 18.10 µM, respectively, on normal lung fibroblasts (MRC-5 cell line) which represent SI of 9.92, 16.20, 24.75 and 10.58, respectively. These results indicate that these compounds are not only with promising CDK2 inhibitory activity and cytotoxicity but also with selectivity towards cancerous cells and so with tolerable and safe effect on normal cells. Cell cycle progression analysis of Panc-1 cell line using the most potent compounds **9h** and **11d** indicated that the newly synthesised compounds cause cell cycle arrest in the G2/M phase which is the main criterion of CDK2 inhibitors confirming the mode of action under investigation. Annexin V-FITC apoptosis assay on Panc-1 cell line using the most potent benzofurans **9h** and **11d** showed that the cell death resulted from the antiproliferative impact of the designed compounds is due to the physiological apoptosis and not due to a non-specific necrosis. Molecular docking simulations showed that, as planned, the designed compounds adopted the common binding pattern of type II inhibitors. The thiosemicarbazide, semicarbazide, and acylhyrazone linkers are accommodated in the interface between the gate area and the allosteric back pocket in the kinase binding site interacting through hydrogen bonding with N-lobe αC helix Glu51 side chain carboxylate and the DFG Asp145 backbone NH. From one side, this binding mode directs the peripheral (un)substituted phenyl moiety towards the hydrophobic region between the gate area and the hinge region, and on the other side, the benzofuran ring is directed towards the hydrophobic allosteric back pocket interacting through hydrophobic interactions with the hydrophobic side chains of the amino acids lining the back pocket. In addition, the phenylpiperazine moiety at 3-position of the benzofuran ring is extended towards a hydrophobic region surrounded by the hydrophobic side chains of the amino acids Leu54, Leu58, Ile63, Cys118, Val123, and Leu143.

## 4. Experimental

### 5.1. Materials and methods

Solvents, reagents and fine chemicals were purchased from Alfa Aesar and Sigma-Aldrich, and were used any without further purification. The NMR spectra were determined by Bruker 400 MHz spectrometer, and ^13 ^C NMR spectra were run at 100 MHz in DMSO-d_6_. FLASH 2000 CHNS/O analyser, Thermo Scientific, was used for elemental analyses.

#### 5.1.2. Preparation of ethyl 3-oxo-2-phenoxybutanoate (3)

Compound **3** was prepared as previously described^83^.

#### 5.1.3. Preparation of ethyl 3-methylbenzofuran-2-carboxylate (4)

The ester **3** (2.5 g, 11.3 mmol) was treated with sulphuric acid in ice-cooled flask 0–5 °C while stirring for 2h. After that, the mixture was added carefully to distilled water while stirring. Then, the benzofuran derivative (**4**) was extracted from the aqueous layer by DCM (3 × 12 ml). The combined organic extracts were washed once with equal volume of 10% aqueous NaHCO_3_ solution then once again with water, and then finally it was dried over anhydrous magnesium sulphate. The residue after solvent evaporation was then recrystallized from methyl alcohol to afford 1.78 gm (77% yield) of white crystals of compound (**4**). The product melts at 49–51 °C, which confirmed its structure by matching with the reported one (48–50 °C)^84^.

#### 5.1.4. Preparation of ethyl 3-(bromomethyl)benzofuran-2-carboxylate (5)

Compound **4** (1.6 g, 8 mmol) was stirred with *N*-bromosuccinimide (NBS) (1.42 g, 8 mmol) in refluxing carbon tetrachloride for 3h. The whole apparatus for this experiment was placed in a well-ventilated fume hood. At the end, the solvent was distilled of and the residue was re-dissolved in hot methyl alcohol to make a saturated solution. Then, the bromo derivative (**5**) was left to crystallise to afford 1.9 g (85% yield), m.p = 87–89 °C (reported^85^ m.p = 86–87 °C).

#### 5.1.5. Preparation of ethyl 3-((4-phenylpiperazin-1-yl)methyl)benzofuran-2-carboxylate (6)

The ester **5** (1.7 g, 6 mmol) was treated with *N*-phenylpiperazine reagent (1.05 g, 6.5 mmol) in the presence of catalytic amount of potassium iodide and potassium carbonate (1.80 g, 13 mmol) as a base. Acetone was used as solvent and the stirred reaction mixture was refluxed for 4h. After that, acetone was evaporated and the residue was triturated in 20 ml of cold water, stirred for 30 min, and then crystallised from ethanol 95% to produce compound **6** as white crystals, m.p = 140–141 °C, yield = 1.97 g, 90% of theoretical. The structure of compound **6** was further confirmed by ^1^H NMR (400-MHz, DMSO-d_6_): δ 8.07 (d, *J =* 7.9 Hz, 1H, Ar˗H at C4 of benzofuran ring), 7.72 (d, *J =* 8.3 Hz, 1H, Ar˗H at C7 of benzofuran ring), 7.55 (t, *J =* 7.6 Hz, 1H, Ar˗H at C6 of benzofuran ring), 7.39 (t, *J =* 7.4 Hz, 1H, Ar˗H at C5 of benzofuran ring), 7.21 (t, *J =* 7.4 Hz, 2H, two Ar˗H at C3,5 phenyl), 6.92 (d, *J =* 7.9 Hz, 2H, two Ar˗H at C2,6 phenyl), 6.78 (t, *J =* 7.2 Hz, 1H, one Ar˗H at C4 phenyl), 4.40 (q, *J =* 7.0 Hz, 2H, methylene of CH_2_CH_3_), 4.10 (s, 2H, methylene exocyclic to C3 of benzofuran ring), 3.13 (s, 4H, two CH_2_N piprazine moiety), 2.62 (s, 4H, two CH_2_N piprazine moiety), 1.39 (t, *J =* 7.0 Hz, 3H, methyl of CH_2_CH_3_).

#### 5.1.6. Preparation of 3-((4-phenylpiperazin-1-yl)methyl)benzofuran-2-carbohydrazide (7)

The ester **6** (2 g, 5.5 mmol) was dissolved in 20 ml of absolute ethanol then (1.1 ml, 22 mmol) of hydrazine hydrate (99%) was added portionwise while stirring at ambient temperature. Then, the whole reaction mixture was heated at reflux for 4h. After that, ethanols as well as most of hydrazine hydrate were evaporated under vacuum using rotavap. The remaining residue was then washed thoroughly for several times with distilled water while vigorous stirring then decantation after each time. Finally, the residue was crystallised from hot ethanol 80% to afford the hydrazide **7** as white crystals, m.p = 243–244 °C, yield = 1.41 g, 73% of theoretical. The structure of the hydrazide **7** was further confirmed by ^1^H NMR. The product was used then in the next steps without further purification. ^1^H NMR (400-MHz, DMSO-d_6_) δ 10.68 (s, 1H, NH hydrazide), 7.97 (d, *J =* 7.8 Hz, 1H, Ar˗H at C4 of benzofuran ring), 7.66 (d, *J =* 8.3 Hz, 1H, Ar˗H at C7 of benzofuran ring), 7.49 (t, *J =* 7.6 Hz, 1H, Ar˗H at C6 of benzofuran ring), 7.37 (t, *J =* 7.4 Hz, 1H, Ar˗H at C5 of benzofuran ring), 7.23 (t, *J =* 7.5 Hz, 2H, two Ar˗H at C3,5 phenyl), 6.94 (d, *J =* 7.9 Hz, 2H, two Ar˗H at C2,6 phenyl), 6.80 (t, *J =* 7.1 Hz, 1H, one Ar˗H at C4 phenyl), 4.66 (s, 2H, NH_2_ hydrazide), 4.06 (s, 2H, methylene at C3 of benzofuran ring), 3.17 (s, 4H, two CH_2_N piprazine moiety), 2.65 (s, 4H, two CH_2_N piprazine moiety).

#### 5.1.7. General method for the synthesis of thiosemicarbazides 9a-i and semicarbazides 11a-e

The hydrazide **7** (0.12 g, 0.34 mmol) and the appropriate derivative of isothiocyanates (**8a-i**, 0.4 mmol) or isocyanate (**10a-e**, 0.4 mmol) (0.4 mmol) were dissolved in hot stirred dry toluene (5 ml). After complete dissolution, the mixture was heated at reflux for 7h. Then, the produced precipitate was filtered by suction, air dried and recrystallized from hot dioxane-toluene mixture (1:5, respectively) to afford the final thiosemicarbazides **9a-i** or semicarbazides **11a-e**, respectively (Full characterisation data are provided in the Supporting Information).

#### 5.1.8. General procedure for the synthesis of hydrazone derivatives 13a-c, 15a,b and 17:

The hydrazide **7** (0.12 g, 0.34 mmol) was dissolved in 10 ml of absolute ethanol. Then, the appropriate derivative of the aldehydes **12a-c** or the ketones **14a**, **b** or **16** (0.38 mmol) was added while stirring till clear solution was obtained. A few drops of acetic acid were added as a catalyst, then the whole reaction mixture was heated at reflux for 11h. After that, the reaction mixture was allowed to cool down to ambient temperature before being poured into a beaker containing 000 gm of crushed ice while stirring. The formed precipitate was allowed to settle down, and then filtered by suction, washed with water and air dried. Recrystallization from ethanol 80% afforded the final hydrazone derivatives **13a-c, 15a,b** and **17** (Full characterisation data are provided in the Supporting Information).

### 5.2. Biological assays

The experimental details for the performed biological assays; Sulforhodamine B (SRB) cytotoxicity, CDK2 kinase, Annexin V-FITC apoptosis, and cell cycle assays^86–89^ were mentioned in the Supporting Information.

### 5.3. Molecular docking study

The experimental details of the adopted protocol, as well as its validation, for the molecular docking simulations were explained in the Supporting Information.

## Supplementary Material

Supplemental Material
