# Supplementary material for: Identification of 3-(piperazinylmethyl)benzofuran derivatives as novel type II CDK2 inhibitors: design, synthesis, biological evaluation, and in silico insights
Source: J Enzyme Inhib Med Chem. 2022 Apr 26;37(1):1227–40. doi: 10.1080/14756366.2022.2062337 (PMC9126595; doi:10.1080/14756366.2022.2062337)

Supporting Information

Identification of 3-(Piperazinylmethyl)benzofuran Derivatives as Novel Type II CDK2 Inhibitors: Design, Synthesis, Biological Evaluation, and *In Silico* Insights

Wagdy M. Eldehna*, Raed M. Maklad, Hadia Almahli, Tarfah Al-Warhi, Eslam B. Elkaeed, Mohammed A.S. Abourehab, Hatem A. Abdel-Aziz, Ahmed M. El Kerdawy

Tables of Contents

1. Anti-proliferative Activity Assay 2

2. Cell Cycle Analysis 2

3. AnnexinV-FITC/PI Apoptosis Assay 3

4. CDK Kinase Inhibitory Activity 3

5. Molecular Docking Study 3

6. Docking poses of the target compounds in CDK2 binding site 4-10

7. Characterisation of the target compounds 11-21

8. NMR Spectra 22-63

1. Anti-proliferative activity assay

The examined human pancreatic cancer (Panc-1), breast cancer (MCF-7), and lung carcinoma (A549) cell lines have been obtained from American Type Culture Collection (ATCC). The cells were maintained in Dulbecco's modified Eagle's medium (DMEM) supplemented with 10% heat inactivated fetal calf serum (GIBCO), penicillin (100 U/ml) and streptomycin (100 µg/ml) at 37 ^oC in humidified atmosphere containing 5% CO_2. Cells at a concentration of 0.50 x 10^6 were grown in a 25 cm^2 flask in 5 ml of culture medium. The anti-proliferative activity of the tested 3-(piperazinylmethyl)benzofurans (**9**, **11**, **13**, **15** and **17**) was measured *in vitro* using the Sulfo-Rhodamine-B stain (SRB) assay. Briefly, cells were plated out in 96-well microtiter plate (0.5X10^4 cells/well) and incubated for 24 h before treatment with the tested hybrids to allow cells to attach to the bottom of the well of the plate. Tested benzofurans were dissolved in DMSO at 1 mg/ml immediately before use and diluted to the appropriate volume just before addition to the cell culture. Different concentrations of tested benzofurans, and Staurosporine were added to the cells (three wells were prepared for each individual dose). Cells were incubated with the hybrids for 48 h at 37°C and in atmosphere of 5% CO_2. After 48 h cells were fixed, washed, and stained for 30 min with 0.4% (w/v) SRB dissolved in 1% acetic acid. Unbound dye was removed by four washes with 1% acetic acid, and attached stain was recovered with Tris-EDTA buffer. Color intensity was measured in an ELISA reader. The relation between percent of surviving fraction and log drug concentration is plotted to get the survival curve for each cell line. The concentration required for 50% inhibition of cell viability (IC_{50}) was calculated and the results are presented in Table 1.

2. Cell Cycle Analysis

Pancreatic cancer Panc-1 cells were treated with benzofurans **9h** and **11d** for 24 h (at their IC_{50} concentration), and then cells were washed twice with ice-cold phosphate buffered saline (PBS). Subsequently, the treated cells were collected by centrifugation, fixed in ice-cold 70% (*v*/*v*) ethanol, washed with PBS, re-suspended with 100 µg/mL RNase, stained with 40 µg/mL PI, and analyzed by flow cytometry using FACS Calibur (Becton Dickinson, BD, Franklin Lakes, NJ, USA). The cell cycle distributions were calculated using CellQuest software 5.1 (Becton Dickinson).

3. Annexin V-FITC Apoptosis Assay

Phosphatidylserine externalization was assayed using Annexin V-FITC/PI apoptosis detection kit (BD Biosciences, USA) according to the manufacturer's instructions. Pancreatic cancer Panc-1 cells were cultured to a monolayer then treated with benzofurans **9h** and **11d** at their IC_{50} concentration. Briefly, cells were then harvested *via* trypsinization, and rinsed twice in PBS followed by binding buffer. Moreover, cells were re-suspended in 100 μL of binding buffer with the addition of 1 μL of FITC-Annexin V followed by an incubation period of 30 min at 4 °C. Cells were then rinsed in binding buffer and resuspended in 150 μL of binding buffer with the addition of 1 μL of DAPI (1 μg/μL in PBS). Cells were then analyzed using the flow cytometer BD FACS Canto II and the results were interpreted with FlowJo7.6.4 software (Tree Star, Ashland, OR, USA).

4. CDK Kinase Inhibitory Activity

The CDK2 enzyme inhibitory activity was determined for benzofurans **9h** and **11d** using the CDK2 Kinase Enzyme System (catalog No. V2971) (Promega, Milan, Italy), according to the manufacturer's instructions.

5. Molecular docking study

All the molecular docking simulations were performed using Molecular Operating Environment (MOE, 2020.0901) software. All minimizations were carried out with MOE until an RMSD gradient of 0.05 $kcal \cdot mol^{-1} Å^{-1}$ with MMFF94x force field and the partial charges were automatically calculated. The X-ray crystallographic structure of CDK2 in its DFG-out inactive conformation co-crystallized with a type II inhibitor (**I**) (PDB ID: 5A14) was downloaded from the protein data bank. The ligand molecule outside the kinase active site and all water molecules were initially removed. Then, the protein was prepared for the docking study using *LigX* protocol in MOE with default options. The co-crystalized ligand was utilized to define the active site for the docking study. Triangle Matcher placement method and London dG scoring function were used for docking. Validation of the used docking protocol was first carried out by self-docking of the co-crystallized ligand (compound **I**) in the active site of the kinase domain giving a docking pose with an energy score (S) = −16.61 kcal/mol and an RMSD of 0.245Å. The validated docking setup was then used to study the ligand-kinase domain interactions of the newly synthesized compounds to confirm their type II-inhibitors binding mode and to determine their SAR to rationalize their binding affinity.

6. Docking poses of the target compounds in CDK2 binding site

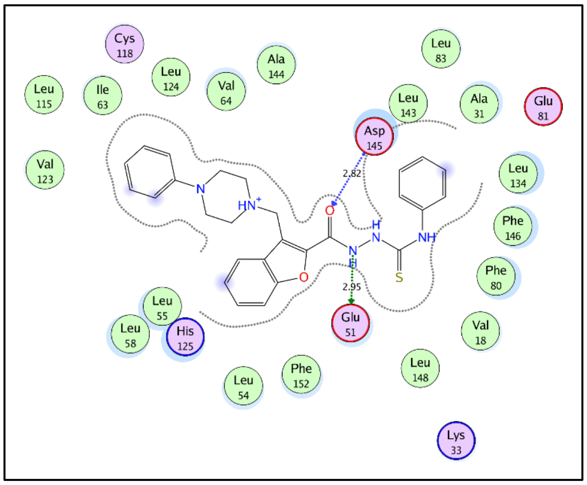

Figure S1. 2D diagram of compound **9a** showing its interaction with the CDK2 binding site.

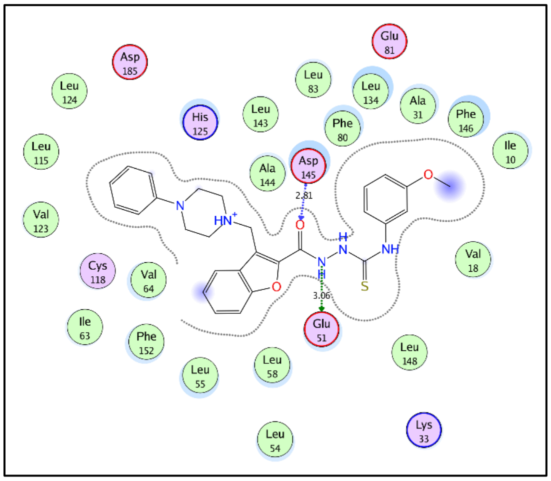

Figure S2. 2D diagram of compound **9b** showing its interaction with the CDK2 binding site.

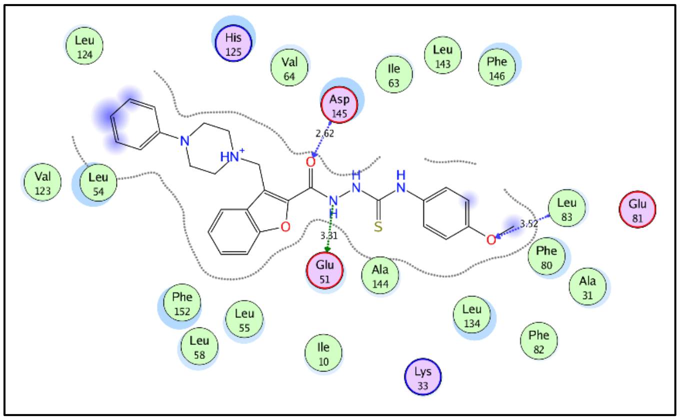

Figure S3. 2D diagram of compound **9c** showing its interaction with the CDK2 binding site.

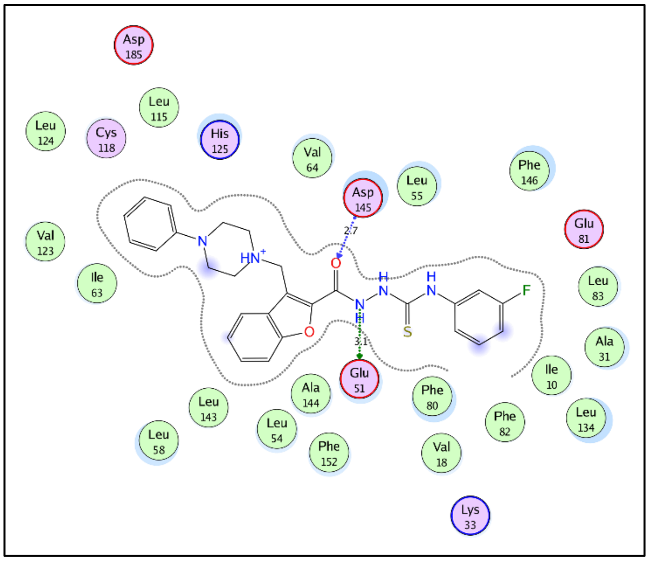

Figure S4. 2D diagram of compound **9d** showing its interaction with the CDK2 binding site.

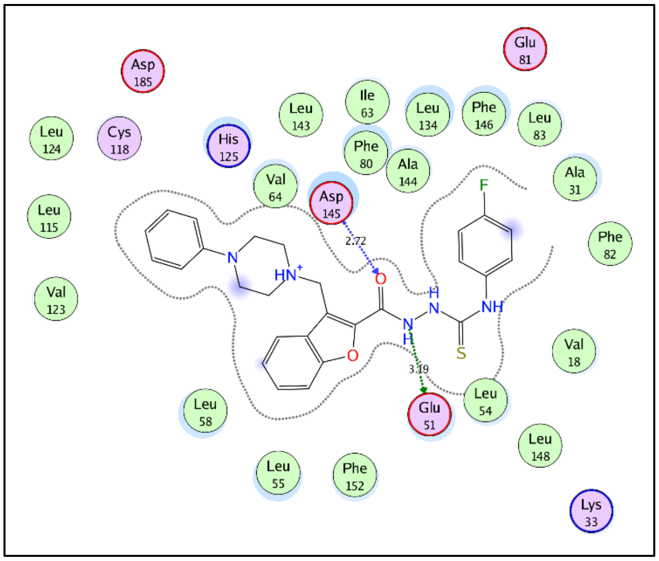

Figure S5. 2D diagram of compound **9e** showing its interaction with the CDK2 binding site.

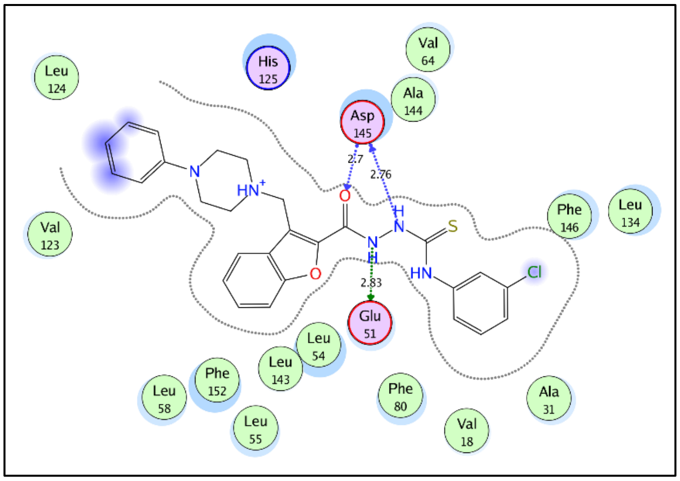

Figure S6. 2D diagram of compound **9f** showing its interaction with the CDK2 binding site.

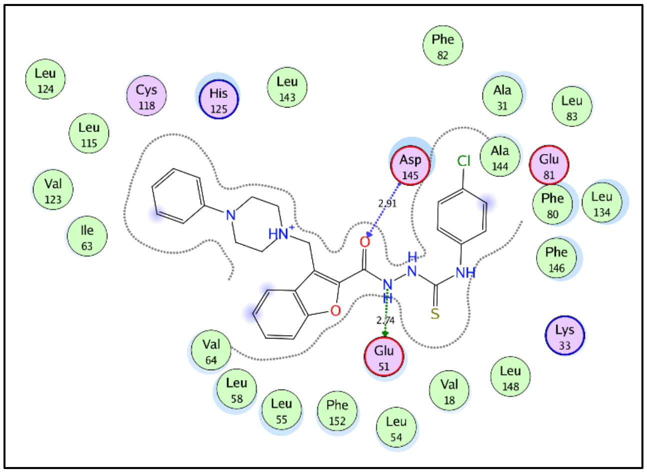

Figure S7. 2D diagram of compound **9g** showing its interaction with the CDK2 binding site.

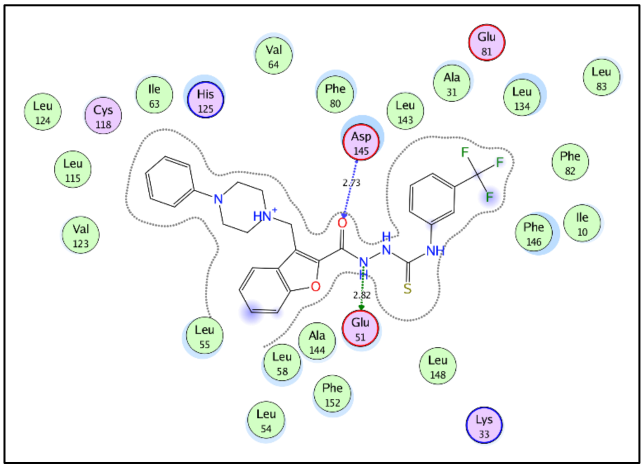

Figure S8. 2D diagram of compound **9h** showing its interaction with the CDK2 binding site.

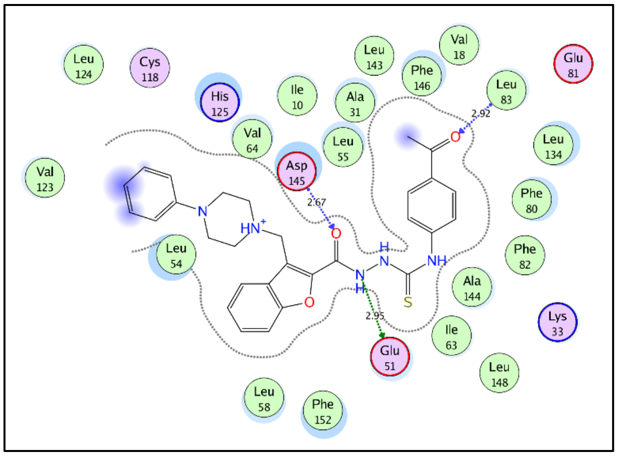

Figure S9. 2D diagram of compound **9i** showing its interaction with the CDK2 binding site.

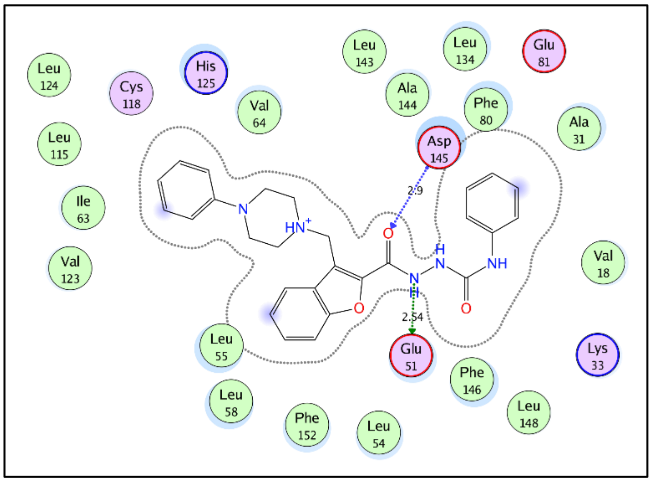

Figure S10. 2D diagram of compound **11a** showing its interaction with the CDK2 binding site.

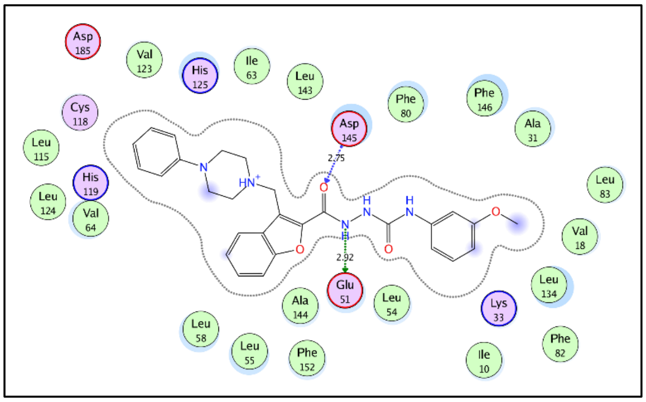

Figure S11. 2D diagram of compound **11b** showing its interaction with the CDK2 binding site.

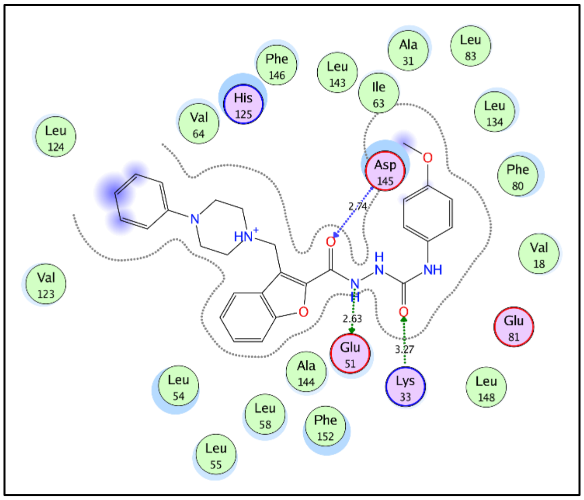

Figure S12. 2D diagram of compound **11c** showing its interaction with the CDK2 binding site.

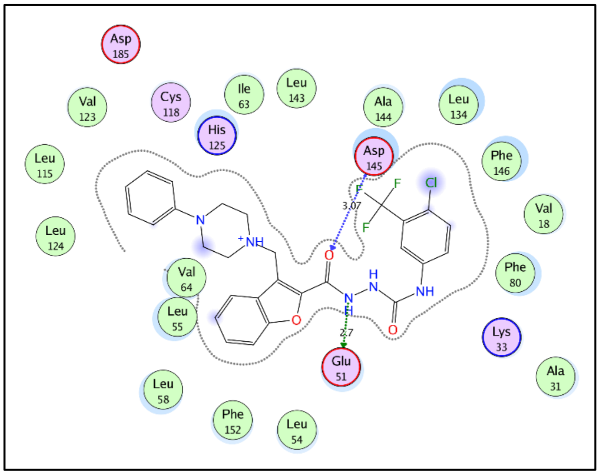

Figure S13. 2D diagram of compound **11d** showing its interaction with the CDK2 binding site.

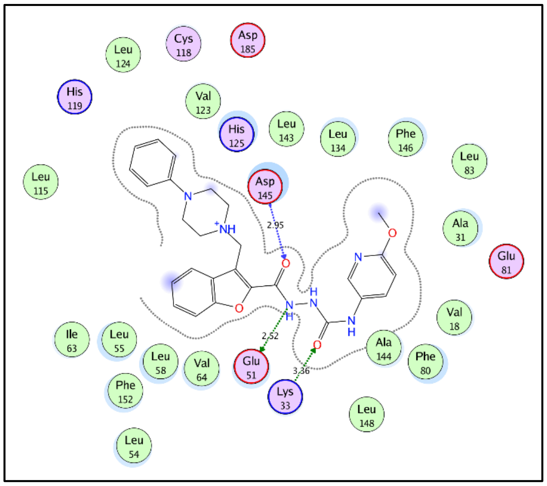

Figure S14. 2D diagram of compound **11e** showing its interaction with the CDK2 binding site.

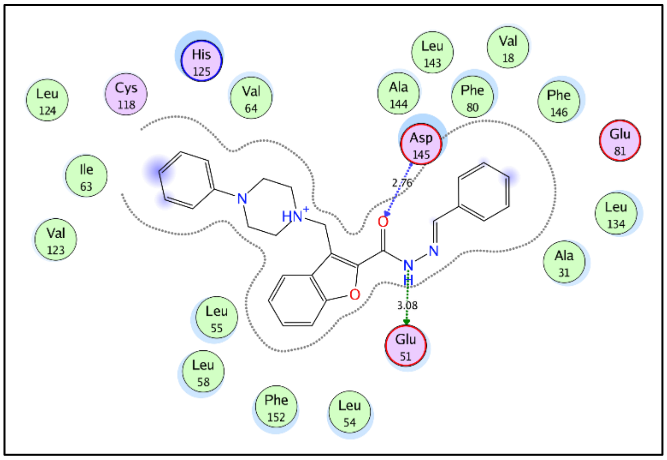

Figure S15. 2D diagram of compound **13a** showing its interaction with the CDK2 binding site.

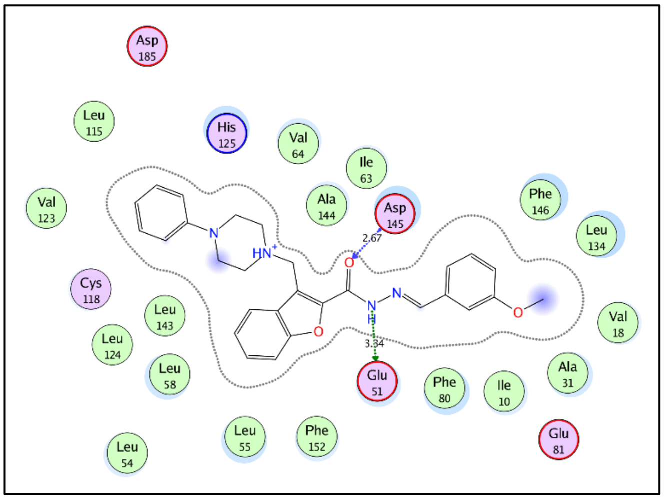

Figure S16. 2D diagram of compound **13b** showing its interaction with the CDK2 binding site.

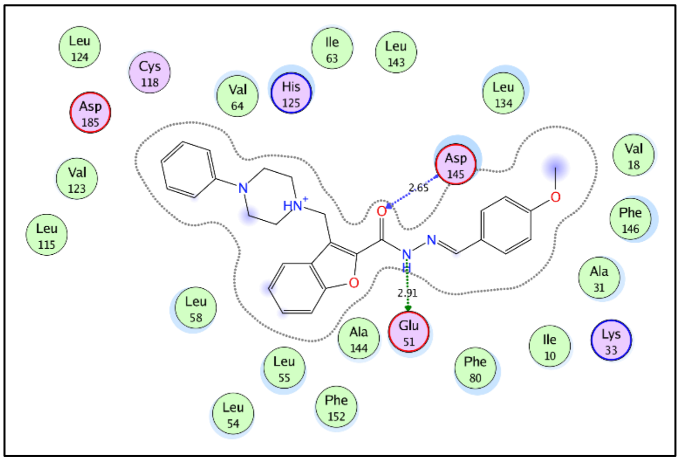

Figure S17. 2D diagram of compound **13c** showing its interaction with the CDK2 binding site.

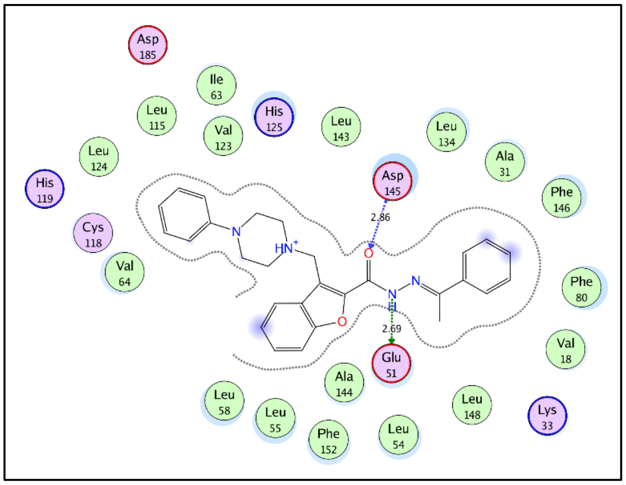

Figure S18. 2D diagram of compound **15a** showing its interaction with the CDK2 binding site.

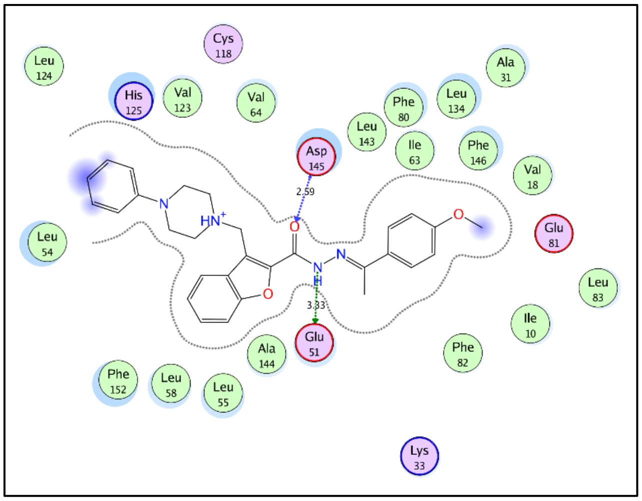

Figure S19. 2D diagram of compound **15b** showing its interaction with the CDK2 binding site.

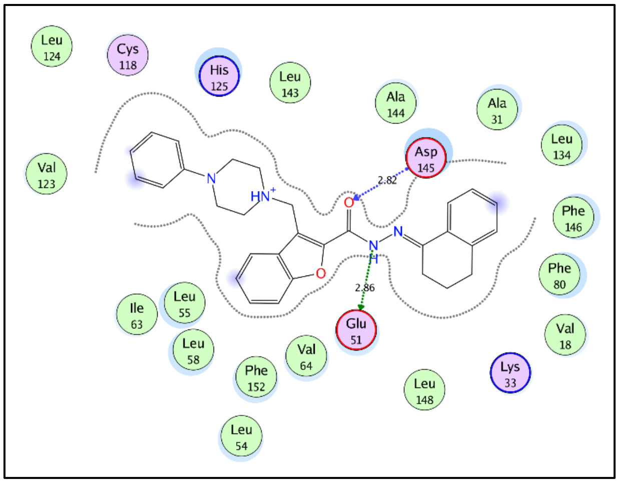

Figure S20. 2D diagram of compound **17** showing its interaction with the CDK2 binding site.

7. Characterisation of the target compounds

N-Phenyl-2-(3-((4-phenylpiperazin-1-yl)methyl)benzofuran-2-carbonyl)hydrazine-1-carbothioamide (9a)

White crystals, m.p = 198-200 ˚C. ^{1}H NMR (400-MHz, DMSO-d_6) δ 12.20 (s, 1H, N4-H thiosemicarbazide), 9.90 (s, 1H, N1-H thiosemicarbazide), 9.84 (s, 1H, N2-H thiosemicarbazide), 8.02 (d, *J*= 7.8 Hz, 1H, Ar-H at C4 of benzofuran ring), 7.74 (d, *J*= 8.3 Hz, 1H, Ar-H at C7 of benzofuran ring), 7.55 (t, *J*= 7.7 Hz, 1H, Ar-H at C6 of benzofuran ring), 7.50 – 7.40 (m, 2H, two Ar-H at C2,6 of phenyl at N4 thiosemicarbazide), 7.35 (t, *J*= 7.4 Hz, 2H, two Ar-H at C3,5 of phenyl at N4 thiosemicarbazide), 7.30 – 7.11 (m, 4H, Ar-H as follows: one at C5 benzofuran, two at C3,5 of phenyl at N4 of piprazine & one at C4 of phenyl at N4 thiosemicarbazide), 6.95 (d, *J*= 8.0 Hz, 2H, Ar-H at C2,6 of phenyl at N4 of piprazine moiety), 6.82 (t, *J*= 7.1 Hz, 1H, Ar-H at C4 of phenyl at N4 of piprazine moiety), 4.15 (s, 2H, methylene exocyclic to C3 of benzofuran ring), 3.20 (s, 4H, two $\underline{CH_2}$N piprazine moiety), 2.75 (s, 4H, two $\underline{CH_2}$N piprazine moiety). ^{13}C NMR (101 MHz, DMSO-d_6) δ 154.16, 151.76, 146.05, 140.15, 138.38, 129.97, 129.93, 129.30, 129.24, 129.11, 128.34, 126.35, 124.64, 122.75, 122.73, 120.08, 116.54, 112.83, 52.81, 50.75, 49.03. Anal. Calcd. for $C_{27}H_{27}N_5O_2S$ (485.61): C, 66.78; H, 5.60; N, 14.42; Found: C, 67.03; H, 5.62; N, 14.49.

N-(3-Methoxyphenyl)-2-(3-((4-phenylpiperazin-1-yl)methyl)benzofuran-2-carbonyl)hydrazine-1-carbothioamide (9b)

White crystals, m.p = 171-173 ˚C. ^{1}H NMR (400-MHz, DMSO-d_6) δ 12.18 (s, 1H, N4-H thiosemicarbazide), 9.86 (s, 2H, N1-H & N2-H thiosemicarbazide), 8.02 (d, *J*= 7.8 Hz, 1H, Ar-H at C4 of benzofuran ring), 7.74 (d, *J*= 8.3 Hz, 1H, Ar-H at C7 of benzofuran ring), 7.55 (t, *J*= 7.7 Hz, 1H, Ar-H at C6 of benzofuran ring), 7.43 (t, *J*= 7.5 Hz, 1H, Ar-H at C5 of benzofuran ring), 7.33 – 7.15 (m, 4H, Ar-H as follows: two at C2,5 of *m*-methoxyphenyl at N4 thiosemicarbazide & two at C3,5 of phenyl at N4 of piprazine moiety), 7.08 (d, *J*= 7.9 Hz, 1H, Ar-H at C6 of *m*-methoxyphenyl), 6.94 (d, *J*= 8.1 Hz, 2H, Ar-H at C2,6 of phenyl at N4 of piprazine moiety), 6.81 (t, *J*= 7.2 Hz, 1H, Ar-H at C4 of phenyl at N4 of piprazine moiety), 6.76 (d, *J*= 7.9 Hz, 1H, Ar-H at C4 of *m*-methoxyphenyl), 4.15 (s, 2H, methylene exocyclic to C3 of benzofuran ring), 3.75 (s, 3H, O$\underline{CH_3}$), 3.20 (s, 4H, two $\underline{CH_2}$N piprazine moiety), 2.75 (s, 4H, two $\underline{CH_2}$N piprazine moiety). ^{13}C NMR (101 MHz, DMSO-d_6) δ 159.97, 154.13, 151.72, 145.99, 141.24, 138.33, 129.91,

129.89, 129.26, 129.20, 128.29, 126.30, 124.59, 122.71, 120.02, 116.48, 112.79, 56.01, 52.78, 50.72, 48.98. Anal. Calcd. for $C_{28}H_{29}N_5O_3S$ (515.63): C, 65.22; H, 5.67; N, 13.58; Found: C, 65.41; H, 5.64; N, 13.50.

_N_-(4-Methoxyphenyl)-2-(3-((4-phenylpiperazin-1-yl)methyl)benzofuran-2-carbonyl)hydrazine-1-carbothioamide (9c)

White crystals, m.p = 182-184 ˚C. ^{1}H NMR (400-MHz, DMSO-d_6) δ 12.16 (s, 1H, N4-H thiosemicarbazide), 9.79 (s, 1H, N1-H thiosemicarbazide), 9.73 (s, 1H, N2-H thiosemicarbazide), 8.01 (d, *J*= 7.8 Hz, 1H, Ar-H at C4 of benzofuran ring), 7.73 (d, *J*= 8.3 Hz, 1H, Ar-H at C7 of benzofuran ring), 7.55 (t, *J*= 7.7 Hz, 1H, Ar-H at C6 of benzofuran ring), 7.42 (t, *J*= 7.5 Hz, 1H, Ar-H at C5 of benzofuran ring), 7.14 – 7.34 (m, 4H, overlapped signals of Ar-H at C2,6 of *p*-methoxyphenyl & Ar-H at C3,5 of phenyl at N4 of piprazine moiety), 6.95 (d, *J*= 8.1 Hz, 2H, Ar-H at C3,5 of *p*-methoxyphenyl), 6.90 (d, *J*= 8.3 Hz, 2H, Ar-H at C2,6 of phenyl at N4 of piprazine moiety), 6.82 (t, *J*= 7.2 Hz, 1H, Ar-H at C4 of phenyl at N4 of piprazine moiety), 4.14 (s, 2H, methylene exocyclic to C3 of benzofuran ring), 3.77 (s, 3H, O$\underline{CH_3}$), 3.20 (s, 4H, two $\underline{CH_2}$N piprazine moiety), 2.74 (s, 4H, two $\underline{CH_2}$N piprazine moiety). ^{13}C NMR (101 MHz, DMSO-d_6) δ 157.74, 154.13, 151.76, 146.04, 138.36, 132.92, 129.94, 129.91, 129.29, 129.22, 128.30, 126.33, 124.60, 122.71, 120.04, 116.51, 114.28, 112.80, 56.18, 52.80, 50.74, 49.02. Anal. Calcd. for $C_{28}H_{29}N_5O_3S$ (515.63): C, 65.22; H, 5.67; N, 13.58; Found: C, 64.95; H, 5.63; N, 13.66.

_N_-(3-Fluorophenyl)-2-(3-((4-phenylpiperazin-1-yl)methyl)benzofuran-2-carbonyl)hydrazine-1-carbothioamide (9d)

White crystals, m.p = 195-197 ˚C. ^{1}H NMR (400-MHz, DMSO-d_6) δ 12.17 (s, 1H, N4-H thiosemicarbazide), 10.00 (s, 2H, N1-H & N2-H thiosemicarbazide), 8.03 (d, *J*= 7.8 Hz, 1H, Ar-H at C4 of benzofuran ring), 7.74 (d, *J*= 8.4 Hz, 1H, Ar-H at C7 of benzofuran ring), 7.56 (t, *J*= 7.7 Hz, 1H, Ar-H at C6 of benzofuran ring), 7.43 (t, *J*= 7.5 Hz, 1H, Ar-H at C5 of benzofuran ring), 7.39 – 7.27 (m, 2H, Ar-H at C2,6 of *m*-fluorophenyl), 7.24 (t, *J*= 7.6 Hz, 2H, Ar-H at C3,5 of phenyl at N4 of piprazine moiety), 7.01 (s, 1H, Ar-H at C4 of phenyl at N4 of piprazine moiety), 6.95 (d, *J*= 8.1 Hz, 2H, Ar-H at C2,6 of phenyl at N4 of piprazine moiety), 6.82 (t, *J*= 7.2 Hz, 1H, Ar-H at C4 of *m*-fluorophenyl), 4.17 (s, 2H, methylene exocyclic to C3 of benzofuran ring), 3.20 (s, 4H, two $\underline{CH_2}$N piprazine moiety), 2.76 (s, 4H, two $\underline{CH_2}$N piprazine moiety). ^{13}C NMR (101

MHz, DMSO-d_6) δ 161.27, 159.02, 156.08, 154.14, 151.70, 145.98, 145.90, 141.92, 141.91, 138.35, 129.93, 129.90, 129.24, 129.21, 128.37, 126.32, 124.65, 122.78, 120.07, 118.94, 116.51, 112.81, 52.79, 50.75, 48.95. Anal. Calcd. for $C_{27}H_{26}FN_5O_2S$ (503.60): C, 64.40; H, 5.20; N, 13.91; Found: C, 64.53; H, 5.17; N, 13.84.

***N*-(4-Fluorophenyl)-2-(3-((4-phenylpiperazin-1-yl)methyl)benzofuran-2-carbonyl)hydrazine-1-carbothioamide (9e)**

White crystals, m.p = 218-220 ˚C. ^{1}H NMR (400-MHz, DMSO-d_6) δ 12.18 (s, 1H, N4-H thiosemicarbazide), 9.88 (s, 2H, N1-H & N2-H thiosemicarbazide), 8.02 (d, *J*= 7.8 Hz, 1H, Ar-H at C4 of benzofuran ring), 7.74 (d, *J*= 8.4 Hz, 1H, Ar-H at C7 of benzofuran ring), 7.55 (t, *J*= 7.7 Hz, 1H, Ar-H at C6 of benzofuran ring), 7.51 – 7.35 (m, 3H, overlapped hydrogen signals of Ar-H at C5 benzofuran & Ar-H at C2,6 of *p*-fluorophenyl), 7.24 (t, *J*= 7.7 Hz, 2H, Ar-H at C3,5 of phenyl at N4 of piprazine moiety), 7.17 (t, *J*= 8.6 Hz, 2H, Ar-H at C3,5 of *p*-fluorophenyl), 6.95 (d, *J*= 8.2 Hz, 2H, Ar-H at C2,6 of phenyl at N4 of piprazine moiety), 6.82 (t, *J*= 7.1 Hz, 1H, Ar-H at C4 of phenyl at N4 of piprazine moiety), 4.15 (s, 2H, methylene exocyclic to C3 of benzofuran ring), 3.19 (s, 4H, two $\underline{CH_2}$N piprazine moiety), 2.74 (s, 4H, two $\underline{CH_2}$N piprazine moiety). ^{13}C NMR (101 MHz, DMSO-d_6) δ 154.10, 151.73, 145.91, 145.86, 136.42, 129.91, 129.25, 129.19, 128.30, 124.58, 122.77, 120.00, 116.47, 115.81, 115.79, 115.58, 115.57, 112.77, 52.81, 50.73, 49.00. Anal. Calcd. for $C_{27}H_{26}FN_5O_2S$ (503.60): C, 64.40; H, 5.20; N, 13.91; Found: C, 64.48; H, 5.22; N, 13.87.

***N*-(3-Chlorophenyl)-2-(3-((4-phenylpiperazin-1-yl)methyl)benzofuran-2-carbonyl)hydrazine-1-carbothioamide (9f)**

White crystals, m.p = 181-183 ˚C. ^{1}H NMR (400-MHz, DMSO-d_6) δ 12.16 (s, 1H, N4-H thiosemicarbazide), 10.01 (s, 1H, N1-H thiosemicarbazide), 9.96 (s, 1H, N2-H thiosemicarbazide), 8.03 (d, *J*= 7.7 Hz, 1H, Ar-H at C4 of benzofuran ring), 7.74 (d, *J*= 8.3 Hz, 1H, Ar-H at C7 of benzofuran ring), 7.56 (t, *J*= 7.7 Hz, 1H, Ar-H at C6 of benzofuran ring), 7.50 (d, *J*= 7.9 Hz, 1H, Ar-H at C6 of *m*-chlorophenyl), 7.43 (t, *J*= 7.8 Hz, 1H, Ar-H at C5 of benzofuran ring), 7.37 (t, *J*= 8.0 Hz, 1H, Ar-H at C5 of *m*-chlorophenyl), 7.32 – 7.15 (m, 4H, overlapped signals of Ar-H at C2,4 of *m*-chlorophenyl & Ar-H at C3,5 of phenyl at N4 of piprazine moiety), 6.95 (d, *J*= 8.0 Hz, 2H, Ar-H at C2,6 of phenyl at N4 of piprazine moiety), 6.82 (t, *J*= 7.2 Hz, 1H, Ar-H at C4 of

phenyl at N4 of piprazine moiety), 4.17 (s, 2H, methylene exocyclic to C3 of benzofuran ring), 3.20 (s, 4H, two $\underline{CH_2}N$ piprazine moiety), 2.76 (s, 4H, two $\underline{CH_2}N$ piprazine moiety). ^{13}C NMR (101 MHz, DMSO-d_6) δ 154.11, 151.70, 141.72, 141.69, 138.32, 130.57, 129.91, 129.88, 129.30, 129.23, 129.19, 128.35, 126.30, 124.61, 122.80, 122.77, 120.04, 116.49, 112.78, 52.80, 50.77, 48.97. Anal. Calcd. for $C_{27}H_{26}ClN_5O_2S$ (520.05): C, 62.36; H, 5.04; N, 13.47; Found: C, 62.17; H, 5.00; N, 13.55.

_N_-(4-Chlorophenyl)-2-(3-((4-phenylpiperazin-1-yl)methyl)benzofuran-2-carbonyl)hydrazine-1-carbothioamide (9g)

White crystals, m.p = 197-199 ˚C. ^{1}H NMR (400-MHz, DMSO-d_6) δ 12.18 (s, 1H, N4-H thiosemicarbazide), 9.94 (s, 2H, N1-H & N2-H thiosemicarbazide), 8.02 (d, *J*= 7.9 Hz, 1H, Ar-H at C4 of benzofuran ring), 7.74 (d, *J*= 8.3 Hz, 1H, Ar-H at C7 of benzofuran ring), 7.66 – 7.47 (m, 3H, overlapped signals of hydrogens of Ar-H at C6 benzofuran & Ar-H at C2,6 of *p*-chlorophenyl), 7.46 – 7.32 (m, 3H, overlapped signals of hydrogens of Ar-H at C5 benzofuran & Ar-H at C3,5 of *p*-chlorophenyl), 7.24 (t, *J*= 7.6 Hz, 2H, Ar-H at C3,5 of phenyl at N4 of piprazine moiety), 6.94 (d, *J*= 8.1 Hz, 2H, Ar-H at C2,6 of phenyl at N4 of piprazine moiety), 6.82 (t, *J*= 7.2 Hz, 1H, Ar-H at C4 of phenyl at N4 of piprazine moiety), 4.16 (s, 2H, methylene exocyclic to C3 of benzofuran ring), 3.19 (s, 4H, two $\underline{CH_2}N$ piprazine moiety), 2.75 (s, 4H, two $\underline{CH_2}N$ piprazine moiety). ^{13}C NMR (101 MHz, DMSO-d_6) δ 154.10, 151.70, 145.86, 145.84, 139.19, 139.15, 129.90, 129.24, 129.19, 128.95, 128.91, 128.32, 124.59, 122.79, 122.77, 120.01, 116.47, 112.77, 52.80, 50.80, 48.96. Anal. Calcd. for $C_{27}H_{26}ClN_5O_2S$ (520.05): C, 62.36; H, 5.04; N, 13.47; Found: C, 62.24; H, 5.01; N, 13.52.

2-(3-((4-Phenylpiperazin-1-yl)methyl)benzofuran-2-carbonyl)-_N_-(3-(trifluoromethyl)phenyl)hydrazine-1-carbothioamide (9h)

White crystals, m.p = 179-181 ˚C. ^{1}H NMR (400-MHz, DMSO-d_6) δ 12.16 (s, 1H, N4-H thiosemicarbazide), 10.08 (s, 2H, N1-H & N2-H thiosemicarbazide), 8.03 (d, *J*= 7.8 Hz, 1H, Ar-H at C4 of benzofuran ring), 7.78-7.98 (m, 2H, overlapped hydrogen signals of Ar-H at C2 & C6 of *m*-(trifluoromethyl phenyl ring), 7.74 (d, *J*= 8.3 Hz, 1H, Ar-H at C7 of benzofuran ring), 7.62 – 7.49 (m, 3H, overlapped signals of hydrogens of Ar-H at C6 benzofuran & Ar-H at C4,5 of *m*-(trifluoromethyl phenyl ring), 7.43 (t, *J*= 7.5 Hz, 1H, Ar-H at C5 of benzofuran ring), 7.23 (t, *J*=

7.7 Hz, 2H, Ar-H at C3,5 of phenyl at N4 of piprazine moiety), 6.94 (d, *J*= 8.1 Hz, 2H, Ar-H at C2,6 of phenyl at N4 of piprazine moiety), 6.81 (t, *J*= 7.2 Hz, 1H, Ar-H at C4 of phenyl at N4 of piprazine moiety), 4.18 (s, 2H, methylene exocyclic to C3 of benzofuran ring), 3.19 (s, 4H, two $\underline{CH_2}$N piprazine moiety), 2.76 (s, 4H, two $\underline{CH_2}$N piprazine moiety). ^{13}C NMR (101 MHz, DMSO-d_6) δ 154.15, 151.73, 145.81, 145.72, 141.03, 130.16, 129.93, 129.25, 128.43, 126.43, 124.67, 123.72, 122.89, 122.87, 120.09, 116.51, 112.82, 52.85, 50.82, 49.02. Anal. Calcd. for $C_{28}H_{26}F_3N_5O_2S$ (553.60): C, 60.75; H, 4.73; N, 12.65; Found: C, 60.92; H, 4.76; N, 12.57.

***N*-(4-Acetylphenyl)-2-(3-((4-phenylpiperazin-1-yl)methyl)benzofuran-2-carbonyl)hydrazine-1-carbothioamide (9i)**

White crystals, m.p = 201-203 ˚C. ^{1}H NMR (400-MHz, DMSO-d_6) δ 12.17 (s, 1H, N4-H thiosemicarbazide), 10.07 (s, 2H, N1-H & N2-H thiosemicarbazide), 8.03 (d, *J*= 7.8 Hz, 1H, Ar-H at C4 of benzofuran ring), 7.93 (d, *J*= 8.2 Hz, 2H, Ar-H at C3,5 of *p*-acetylphenyl ring), 7.75 (d, *J*= 8.15 Hz, 1H, Ar-H at C7 of benzofuran ring), 7.56 (t, *J*= 7.7 Hz, 1H, Ar-H at C6 of benzofuran ring), 7.43 (t, *J*= 7.5 Hz, 1H, Ar-H at C5 of benzofuran ring), 7.32 – 7.16 (m, 4H, overlapped signals of Ar-H at C2,6 of *p*-acetylphenyl ring & Ar-H at C3,5 of phenyl at N4 of piprazine moiety), 6.94 (d, *J*= 7.8 Hz, 2H, Ar-H at C2,6 of phenyl at N4 of piprazine moiety), 6.81 (t, *J*= 7.2 Hz, 1H, Ar-H at C4 of phenyl at N4 of piprazine moiety), 4.19 (s, 2H, methylene exocyclic to C3 of benzofuran ring), 3.19 (s, 4H, two $\underline{CH_2}$N piprazine moiety), 2.78 (s, 4H, two $\underline{CH_2}$N piprazine moiety), 2.57 (s, 3H, $\underline{CH_3}$C=O). ^{13}C NMR (101 MHz, DMSO-d_6) δ 166.31, 154.11, 151.63, 144.66, 138.32, 131.14, 129.90, 129.88, 129.21, 129.19, 128.36, 126.30, 124.64, 122.81, 122.71, 121.36, 120.05, 116.49, 112.79, 52.75, 50.69, 48.89, 27.52. Anal. Calcd. for $C_{29}H_{29}N_5O_3S$ (527.64): C, 66.01; H, 5.54; N, 13.27; Found: C, 65.83; H, 5.56; N, 13.32.

***N*-Phenyl-2-(3-((4-phenylpiperazin-1-yl)methyl)benzofuran-2-carbonyl)hydrazine-1-carboxamide (11a)**

White crystals, m.p = 205-207 ˚C. ^{1}H NMR (400-MHz, DMSO-d_6) δ 12.20 (s, 1H, N4-H semicarbazide), [9.91 (s, 1H), 9.84 (s, 1H), N1-H & N2-H semicarbazide], 8.02 (d, *J*= 7.8 Hz, 1H, Ar-H at C4 of benzofuran ring), 7.74 (d, *J*= 8.3 Hz, 1H, Ar-H at C7 of benzofuran ring), 7.55 (t, *J*= 7.7 Hz, 1H, Ar-H at C6 of benzofuran ring), 7.48 (brs, 2H, Ar-H at C2,6 of phenyl at N4 semicarbazide), 7.42 (t, *J*= 7.5 Hz, 1H, Ar-H at C5 of benzofuran ring), 7.35 (t, *J*= 7.5 Hz, 2H,

Ar-H at C3,5 of phenyl at N4 semicarbazide), 7.28 – 7.15 (m, 3H, overlapped signals of Ar-H at C3,5 of phenyl at N4 of piprazine & Ar-H at C4 of phenyl at N4 semicarbazide), 6.95 (d, *J*= 8.1 Hz, 2H, Ar-H at C2,6 of phenyl at N4 of piprazine moiety), 6.82 (t, *J*= 7.2 Hz, 1H, Ar-H at C4 of phenyl at N4 of piprazine moiety), 4.15 (s, 2H, methylene exocyclic to C3 of benzofuran ring), 3.20 (s, 4H, two $\underline{CH_2}N$ piprazine moiety), 2.75 (s, 4H, two $\underline{CH_2}N$ piprazine moiety). ^{13}C NMR (101 MHz, DMSO-d_6) δ 154.15, 151.75, 146.03, 140.15, 138.36, 129.95, 129.92, 129.29, 129.23, 129.11, 128.33, 126.34, 124.62, 122.74, 121.30, 120.06, 116.52, 112.81, 52.81, 50.75, 49.02. Anal. Calcd. for $C_{27}H_{27}N_5O_3$ (469.55): C, 69.07; H, 5.80; N, 14.92; Found: C, 68.91; H, 5.83; N, 14.96.

_N_-(3-Methoxyphenyl)-2-(3-((4-phenylpiperazin-1-yl)methyl)benzofuran-2-carbonyl)hydrazine-1-carboxamide (11b)

White crystals, m.p = 217-219 ˚C. ^1H NMR (400-MHz, DMSO-d_6) δ 11.59 (s, 1H, N4-H semicarbazide), [8.95 (s, 1H), 8.44 (s, 1H), N1-H & N2-H semicarbazide], 8.03 (d, *J*= 7.8 Hz, 1H, Ar-H at C4 of benzofuran ring), 7.73 (d, *J*= 8.3 Hz, 1H, Ar-H at C7 of benzofuran ring), 7.55 (t, *J*= 7.7 Hz, 1H, Ar-H at C6 of benzofuran ring), 7.42 (t, *J*= 7.5 Hz, 1H, Ar-H at C5 of benzofuran ring), 7.26 – 7.14 (m, 4H, overlapped signals of Ar-H at C2,6 of *m*-methoxyphenyl ring & Ar-H at C3,5 of phenyl at N4 of piprazine moiety), 7.03 (d, *J*= 7.9 Hz, 1H, Ar-H at C5 of *m*-methoxyphenyl ring), 6.94 (d, *J*= 8.1 Hz, 2H, Ar-H at C2,6 of phenyl at N4 of piprazine moiety), 6.80 (t, *J*= 7.2 Hz, 1H, Ar-H at C4 of phenyl at N4 of piprazine moiety), 6.58 (d, *J*= 8.1 Hz, 1H, Ar-H at C4 of *m*-methoxyphenyl ring), 4.10 (s, 2H, methylene exocyclic to C3 of benzofuran ring), 3.73 (s, 3H, $O\underline{CH_3}$), 3.19 (s, 4H, two $\underline{CH_2}N$ piprazine moiety), 2.70 (s, 4H, two $\underline{CH_2}N$ piprazine moiety). ^{13}C NMR (101 MHz, DMSO-d_6) δ 160.52, 159.89, 155.92, 154.15, 151.81, 145.56, 141.77, 130.40, 129.88, 129.25, 128.26, 124.55, 122.92, 121.68, 119.89, 116.43, 112.76, 111.78, 108.30, 105.23, 55.84, 53.00, 50.98, 49.04. Anal. Calcd. for $C_{28}H_{29}N_5O_4$ (499.57): C, 67.32; H, 5.85; N, 14.02; Found: C, 67.14; H, 5.88; N, 13.95.

_N_-(4-Methoxyphenyl)-2-(3-((4-phenylpiperazin-1-yl)methyl)benzofuran-2-carbonyl)hydrazine-1-carboxamide (11c)

White crystals, m.p = 252-254 ˚C. ^1H NMR (400-MHz, DMSO-d_6) δ 11.56 (s, 1H, N4-H semicarbazide), [8.76 (s, 1H), 8.34 (s, 1H), N1-H & N2-H semicarbazide], 8.02 (d, *J*= 7.5 Hz, 1H, Ar-H at C4 of benzofuran ring), 7.72 (d, *J*= 8.1 Hz, 1H, Ar-H at C7 of benzofuran ring), 7.55 (t,

J= 7.4 Hz, 1H, Ar-H at C6 of benzofuran ring), 7.47 – 7.33 (m, 3H, overlapped signals of Ar-H at C5 benzofuran & Ar-H at C2,6 of *p*-methoxyphenyl at N4 of piprazine moiety), 7.22 (t, *J*= 7.1 Hz, 2H, Ar-H at C3,5 of phenyl at N4 of piprazine moiety), 6.94 (d, *J*= 7.6 Hz, 2H, Ar-H at C2,6 of phenyl at N4 of piprazine moiety), 6.87 (d, *J*= 8.1 Hz, 2H, Ar-H at C3,5 of *p*-methoxyphenyl at N4 of piprazine moiety), 6.80 (t, *J*= 6.8 Hz, 1H, Ar-H at C4 of phenyl at N4 of piprazine moiety), 4.10 (s, 2H, methylene exocyclic to C3 of benzofuran ring), 3.74 (s, 3H, O$\underline{CH_3}$), 3.18 (s, 4H, two $\underline{CH_2}$N piprazine moiety), 2.70 (s, 4H, two $\underline{CH_2}$N piprazine moiety). ^{13}C NMR (101 MHz, DMSO-d_6) δ 161.02, 160.01, 156.34, 155.52, 154.17, 151.83, 145.70, 133.53, 129.92, 129.29, 128.27, 124.58, 122.91, 121.43, 119.94, 116.48, 114.81, 112.80, 56.12, 53.00, 50.98, 49.09. Anal. Calcd. for $C_{28}H_{29}N_5O_4$ (499.57): C, 67.32; H, 5.85; N, 14.02; Found: C, 67.18; H, 5.87; N, 13.98.

_N_-(4-Chloro-3-(trifluoromethyl)phenyl)-2-(3-((4-phenylpiperazin-1-yl)methyl)benzofuran-2-carbonyl)hydrazine-1-carboxamide (11d)

White crystals, m.p = 229-231 ˚C. ^{1}H NMR (400-MHz, DMSO-d_6) δ 11.57 (s, 1H, N4-H semicarbazide), [9.42 (s, 1H), 8.74 (s, 1H), N1-H & N2-H semicarbazide], 8.11 (s, 1H, Ar-H at C2 of 4-chloro-3-(trifluoromethyl)phenyl ring), 8.04 (d, *J*= 7.8 Hz, 1H, Ar-H at C4 of benzofuran ring), 7.82 (d, *J*= 7.0 Hz, 1H, Ar-H at C5 of 4-chloro-3-(trifluoromethyl)phenyl ring), 7.73 (d, *J*= 8.3 Hz, 1H, Ar-H at C7 of benzofuran ring), 7.63 (d, *J*= 8.8 Hz, 1H, Ar-H at C6 of 4-chloro-3-(trifluoromethyl)phenyl ring), 7.56 (t, *J*= 7.7 Hz, 1H, Ar-H at C6 of benzofuran ring), 7.42 (t, *J*= 7.4 Hz, 1H, Ar-H at C5 of benzofuran ring), 7.22 (t, *J*= 7.6 Hz, 2H, Ar-H at C3,5 of phenyl at N4 of piprazine moiety), 6.93 (d, *J*= 8.1 Hz, 2H, Ar-H at C2,6 of phenyl at N4 of piprazine moiety), 6.80 (t, *J*= 7.1 Hz, 1H, Ar-H at C4 of phenyl at N4 of piprazine moiety), 4.12 (s, 2H, methylene exocyclic to C3 of benzofuran ring), 3.17 (s, 4H, two $\underline{CH_2}$N piprazine moiety), 2.70 (s, 4H, two $\underline{CH_2}$N piprazine moiety). ^{13}C NMR (101 MHz, DMSO-d_6) δ 160.17, 158.87, 156.03, 154.15, 151.81, 145.39, 140.30, 132.88, 129.86, 129.23, 128.35, 124.57, 124.30, 123.39, 123.04, 122.43, 122.05, 119.92, 116.42, 112.76, 67.32, 53.05, 51.06, 49.10. Anal. Calcd. for $C_{28}H_{25}ClF_3N_5O_3$ (571.99): C, 58.80; H, 4.41; N, 12.24; Found: C, 59.02; H, 4.38; N, 12.26.

_N_-(6-Methoxypyridin-3-yl)-2-(3-((4-phenylpiperazin-1-yl)methyl)benzofuran-2-carbonyl)hydrazine-1-carboxamide (11e)

White crystals, m.p = 233-236 ˚C. ^1H NMR (400-MHz, DMSO-d_6) δ 11.55 (s, 1H, N4-H semicarbazide), [8.91 (s, 1H), 8.52 (s, 1H), N1-H & N2-H semicarbazide], 8.23 (s, 1H, Ar-H at C2 of 6-methoxypyridin-3-yl ring), 8.03 (d, *J*= 7.8 Hz, 1H, Ar-H at C4 of benzofuran ring), 7.84 (d, *J*= 8.9 Hz, 1H, Ar-H at C4 of 6-methoxypyridin-3-yl ring), 7.72 (d, *J*= 8.3 Hz, 1H, Ar-H at C7 of benzofuran ring), 7.55 (t, *J*= 7.7 Hz, 1H, Ar-H at C6 of benzofuran ring), 7.42 (t, *J*= 7.5 Hz, 1H, Ar-H at C5 of benzofuran ring), 7.22 (t, *J*= 7.6 Hz, 2H, Ar-H at C3,5 of phenyl at N4 of piprazine moiety), 6.94 (d, *J*= 8.1 Hz, 2H, Ar-H at C2,6 of phenyl at N4 of piprazine moiety), 6.78-6.83 (m, 2H, overlapped signal of Ar-H at C4 of phenyl at N4 of piprazine & Ar-H at C6 of 6-methoxypyridin-3-yl ring), 4.11 (s, 2H, methylene exocyclic to C3 of benzofuran ring), 3.83 (s, 3H, O$\underline{CH_3}$), 3.18 (s, 4H, two $\underline{CH_2}$N piprazine moiety), 2.70 (s, 4H, two $\underline{CH_2}$N piprazine moiety). ^{13}C NMR (101 MHz, DMSO-d_6) δ 160.05, 156.51, 154.13, 151.78, 145.55, 138.36, 132.55, 131.34, 129.90, 129.22, 128.29, 124.60, 122.96, 121.78, 119.91, 116.46, 112.78, 110.86, 54.03, 52.99, 50.99, 49.08. Anal. Calcd. for $C_{27}H_{28}N_6O_4$ (500.56): C, 64.79; H, 5.64; N, 16.79; Found: C, 64.53; H, 5.67; N, 16.72.

***N'*-Benzylidene-3-((4-phenylpiperazin-1-yl)methyl)benzofuran-2-carbohydrazide (13a)**

White crystals, m.p = 192-194 ˚C. ^1H NMR (400-MHz, DMSO-d_6) δ 12.79 (s, 1H, NH hydrazone), 8.53 (s, 1H, H-C=N hydrazone), 8.06 (d, *J*= 7.8 Hz, 1H, Ar-H at C4 of benzofuran ring), 7.86 – 7.66 (m, 3H, overlapped signals of Ar-H at C7 benzofuran & Ar-H at C2,6 of benzylidene moiety), 7.63 – 7.46 (m, 4H, overlapped signals of Ar-H at C6 benzofuran & Ar-H at C3,4,5 of benzylidene moiety), 7.43 (t, *J*= 7.5 Hz, 1H, Ar-H at C5 of benzofuran ring), 7.23 (t, *J*= 7.6 Hz, 2H, Ar-H at C3,5 of phenyl at N4 of piprazine moiety), 6.96 (d, *J*= 8.1 Hz, 2H, Ar-H at C2,6 of phenyl at N4 of piprazine moiety), 6.80 (t, *J*= 7.2 Hz, 1H, Ar-H at C4 of phenyl at N4 of piprazine moiety), 4.17 (s, 2H, methylene exocyclic to C3 of benzofuran ring), 3.21 (s, 4H, two $\underline{CH_2}$N piprazine moiety), 2.73 (s, 4H, two $\underline{CH_2}$N piprazine moiety). ^{13}C NMR (101 MHz, DMSO-d_6) δ 156.50, 154.17, 151.86, 149.50, 145.34, 135.08, 131.31, 129.89, 129.41, 128.42, 128.20, 124.61, 123.20, 122.48, 119.88, 116.45, 112.78, 53.23, 51.18, 49.14. Anal. Calcd. for $C_{27}H_{26}N_4O_2$ (438.53): C, 73.95; H, 5.98; N, 12.78; Found: C, 74.18; H, 6.00; N, 12.73.

***N'*-(3-Methoxybenzylidene)-3-((4-phenylpiperazin-1-yl)methyl)benzofuran-2-carbohydrazide (13b)**

White crystals, m.p = 176-178 ˚C. ^{1}H NMR (400-MHz, DMSO-d_6) δ 12.79 (s, 1H, NH hydrazone), 8.50 (s, 1H, H-C=N hydrazone), 8.06 (d, *J*= 7.9 Hz, 1H, Ar-H at C4 of benzofuran ring), 7.74 (d, *J*= 8.2 Hz, 1H, Ar-H at C7 of benzofuran ring), 7.57 (t, *J*= 7.7 Hz, 1H, Ar-H at C6 of benzofuran ring), 7.43 (t, *J*= 7.58 Hz, 2H, overlapped signals of Ar-H at C5 benzofuran & Ar-H at C6 of *m*-methoxybenzylidene moiety), 7.28-7.35 (m, 2H, Ar-H at C2,5 of *m*-methoxybenzylidene moiety), 7.23 (t, *J*= 7.7 Hz, 2H, Ar-H at C3,5 of phenyl at N4 of piprazine moiety), 7.07 (d, *J*= 7.9 Hz, 1H, Ar-H at C4 of *m*-methoxybenzylidene moiety), 6.96 (d, *J*= 8.1 Hz, 2H, Ar-H at C2,6 of phenyl at N4 of piprazine moiety), 6.80 (t, *J*= 7.2 Hz, 1H, Ar-H at C4 of phenyl at N4 of piprazine moiety), 4.17 (s, 2H, methylene exocyclic to C3 of benzofuran ring), 3.21 (s, 4H, two $\underline{CH_2}$N piprazine moiety), 2.73 (s, 4H, two $\underline{CH_2}$N piprazine moiety). ^{13}C NMR (101 MHz, DMSO-d_6) δ 160.52, 156.50, 154.16, 151.86, 149.38, 145.29, 136.50, 131.01, 129.89, 129.40, 128.43, 124.61, 123.22, 122.55, 121.27, 119.87, 117.48, 116.44, 112.78, 112.09, 56.16, 53.23, 51.17, 49.12. Anal. Calcd. for $C_{28}H_{28}N_4O_3$ (468.56): C, 71.78; H, 6.02; N, 11.96; Found: C, 71.99; H, 5.98; N, 11.90.

***N'*-(4-Methoxybenzylidene)-3-((4-phenylpiperazin-1-yl)methyl)benzofuran-2-carbohydrazide (13c)**

White crystals, m.p = 181-182 ˚C. ^{1}H NMR (400-MHz, DMSO-d_6) δ 12.67 (s, 1H, NH hydrazone), 8.46 (s, 1H, H-C=N hydrazone), 8.05 (d, *J*= 7.9 Hz, 1H, Ar-H at C4 of benzofuran ring), 7.78 – 7.65 (m, 3H, overlapped signals of Ar-H at C7 benzofuran & Ar-H at C2,6 of *p*-methoxybenzylidene moiety), 7.56 (t, *J*= 7.7 Hz, 1H, Ar-H at C6 of benzofuran ring), 7.42 (t, *J*= 7.4 Hz, 1H, Ar-H at C5 of benzofuran ring), 7.23 (t, *J*= 7.5 Hz, 2H, Ar-H at C3,5 of phenyl at N4 of piprazine moiety), 7.08 (d, *J*= 8.1 Hz, 2H, Ar-H at C3,5 of *p*-methoxybenzylidene moiety), 6.96 (d, *J*= 8.1 Hz, 2H, Ar-H at C2,6 of phenyl at N4 of piprazine moiety), 6.80 (t, *J*= 7.1 Hz, 1H, Ar-H at C4 of phenyl at N4 of piprazine moiety), 4.15 (s, 2H, methylene exocyclic to C3 of benzofuran ring), 3.85 (s, 3H), 3.21 (s, 4H, two $\underline{CH_2}$N piprazine moiety), 2.72 (s, 4H, two $\underline{CH_2}$N piprazine moiety). ^{13}C NMR (101 MHz, DMSO-d_6) δ 161.99, 156.32, 154.14, 151.86, 149.37, 145.53, 129.90, 129.85, 129.45, 128.32, 127.61, 124.58, 123.13, 122.10, 119.90, 116.46, 115.39, 112.77,

56.31, 53.21, 51.16, 49.14. Anal. Calcd. for $C_{28}H_{28}N_4O_3$ (468.56): C, 71.78; H, 6.02; N, 11.96; Found: C, 71.51; H, 6.05; N, 12.02.

_N'_-(1-Phenylethylidene)-3-((4-phenylpiperazin-1-yl)methyl)benzofuran-2-carbohydrazide (15a)

White crystals, m.p = 191-193 ˚C. ^{1}H NMR (400-MHz, DMSO-d_6) δ 11.46 (s, 1H, NH hydrazone), 8.05 (d, *J*= 7.8 Hz, 1H, Ar-H at C4 of benzofuran ring), 7.93 (s, 2H, Ar-H at C2,6 of benzylidene moiety), 7.77 (d, *J*= 8.3 Hz, 1H, Ar-H at C7 of benzofuran ring), 7.56 (t, *J*= 7.7 Hz, 1H, Ar-H at C6 of benzofuran ring), 7.48 (s, 3H, overlapped signals of Ar-H at C3,4,5 of benzylidene moiety), 7.43 (t, *J*= 7.5 Hz, 1H, Ar-H at C5 of benzofuran ring), 7.23 (t, *J*= 7.5 Hz, 2H, Ar-H at C3,5 of phenyl at N4 of piprazine moiety), 6.95 (d, *J*= 8.0 Hz, 2H, Ar-H at C2,6 of phenyl at N4 of piprazine moiety), 6.80 (t, *J*= 7.2 Hz, 1H, Ar-H at C4 of phenyl at N4 of piprazine moiety), 4.12 (s, 2H, methylene exocyclic to C3 of benzofuran ring), 3.19 (s, 4H, two $\underline{CH_2}$N piprazine moiety), 2.72 (s, 4H, two $\underline{CH_2}$N piprazine moiety), 2.46 (s, 3H, $\underline{CH_3}$-C=N). ^{13}C NMR (101 MHz, DMSO-d_6) δ 156.60, 155.99, 154.27, 151.79, 146.10, 138.65, 130.67, 129.90, 129.52, 129.38, 128.31, 127.50, 124.63, 122.84, 120.93, 119.91, 116.42, 112.88, 53.40, 51.01, 48.72, 15.98. Anal. Calcd. for $C_{28}H_{28}N_4O_2$ (452.56): C, 74.31; H, 6.24; N, 12.38; Found: C, 74.48; H, 6.25; N, 12.34.

_N'_-(1-(4-Methoxyphenyl)ethylidene)-3-((4-phenylpiperazin-1-yl)methyl)benzofuran-2-carbohydrazide (15b)

White crystals, m.p = 212-214 ˚C. ^{1}H NMR (400-MHz, DMSO-d_6) δ 11.39 (s, 1H, NH hydrazone), 8.04 (d, *J*= 7.9 Hz, 1H, Ar-H at C4 of benzofuran ring), 7.89 (d, *J*= 8.3 Hz, 2H, Ar-H at C2,6 of *p*-methoxybenzylidene moiety), 7.76 (d, *J*= 8.2 Hz, 1H, Ar-H at C7 of benzofuran ring), 7.55 (t, *J*= 7.7 Hz, 1H, Ar-H at C6 of benzofuran ring), 7.43 (t, *J*= 7.5 Hz, 1H, Ar-H at C5 of benzofuran ring), 7.23 (t, *J*= 7.6 Hz, 2H, Ar-H at C3,5 of phenyl at N4 of piprazine moiety), 7.04 (d, *J*= 8.5 Hz, 2H, Ar-H at C3,5 of *p*-methoxybenzylidene moiety), 6.96 (d, *J*= 8.0 Hz, 2H, Ar-H at C2,6 of phenyl at N4 of piprazine moiety), 6.80 (t, *J*= 7.2 Hz, 1H, Ar-H at C4 of phenyl at N4 of piprazine moiety), 4.11 (s, 2H, methylene exocyclic to C3 of benzofuran ring), 3.84 (s, 3H, O$\underline{CH_3}$), 3.20 (s, 4H, two $\underline{CH_2}$N piprazine moiety), 2.72 (s, 4H, two $\underline{CH_2}$N piprazine moiety), 2.42 (s, 3H, $\underline{CH_3}$-C=N). ^{13}C NMR (101 MHz, DMSO-d_6) δ 161.55, 156.47, 156.13, 154.25, 151.83, 146.21,

131.00, 129.90, 129.58, 129.08, 128.24, 124.60, 122.82, 120.71, 119.91, 116.42, 114.73, 112.85, 56.24, 53.42, 51.03, 48.76, 15.85. Anal. Calcd. for $C_{29}H_{30}N_4O_3$ (482.58): C, 72.18; H, 6.27; N, 11.61; Found: C, 71.93; H, 6.31; N, 11.65.

***N'*-(3,4-Dihydronaphthalen-1(2H)-ylidene)-3-((4-phenylpiperazin-1-yl)methyl)benzofuran-2-carbohydrazide (17)**

White crystals, m.p = 161-162 ˚C. ^{1}H NMR (400-MHz, DMSO-d_6) δ 11.37 (s, 1H, NH hydrazone), 8.17 (d, *J*= 7.4 Hz, 1H, Ar-H at C4 of benzofuran ring), 8.04 (d, *J*= 7.8 Hz, 1H, Ar-H at C8 of dihydronaphthaylidene moiety), 7.77 (d, *J*= 8.3 Hz, 1H, Ar-H at C7 of benzofuran ring), 7.56 (t, *J*= 7.6 Hz, 1H, Ar-H at C6 of benzofuran ring), 7.43 (t, *J*= 7.5 Hz, 1H, Ar-H at C5 of benzofuran ring), 7.40 – 7.28 (m, 2H, overlapped signals of Ar-H at C6,7 of dihydronaphthaylidene moiety), 7.28 – 7.18 (m, 3H, overlapped signals of Ar-H at C3,5 of phenyl at N4 of piprazine & Ar-H at C5 of dihydronaphthaylidene moiety), 6.96 (d, *J*= 8.0 Hz, 2H, Ar-H at C2,6 of phenyl at N4 of piprazine moiety), 6.80 (t, *J*= 7.2 Hz, 1H, Ar-H at C4 of phenyl at N4 of piprazine moiety), 4.10 (s, 2H, methylene exocyclic to C3 of benzofuran ring), 3.20 (s, 4H, two $\underline{CH_2}$N piprazine moiety), 2.92 – 2.78 (m, 4H, aliph. H at C2,4 of dihydronaphthaylidene moiety), 2.72 (s, 4H, two $\underline{CH_2}$N piprazine moiety), 1.99 – 1.80 (m, 2H, aliph. H at C3 of dihydronaphthaylidene moiety). ^{13}C NMR (101 MHz, DMSO-d_6) δ 156.48, 155.01, 154.30, 151.81, 146.20, 141.25, 132.94, 130.58, 129.91, 129.64, 129.59, 128.30, 127.29, 125.74, 124.62, 122.78, 120.70, 119.90, 116.38, 112.87, 53.45, 50.95, 48.65, 29.73, 27.54, 22.24. Anal. Calcd. for $C_{30}H_{30}N_4O_2$ (478.60): C, 75.29; H, 6.32; N, 11.71; Found: C, 75.06; H, 6.35; N, 11.67.

1H NMR spectrum of compound 6 in DMSO-d6 at 400 MHz

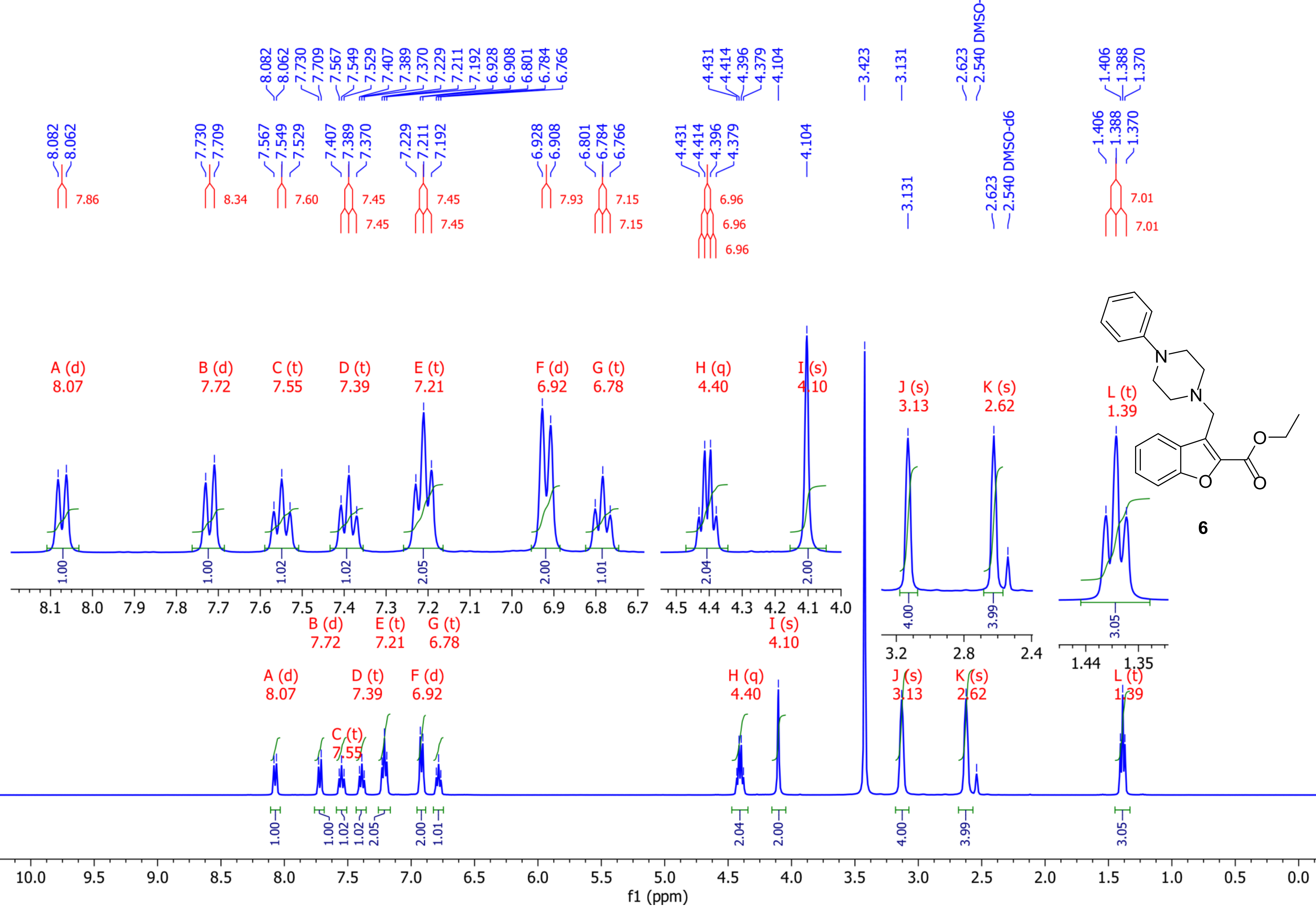

1H NMR spectrum of compound 7 in DMSO-d6 at 400 MHz

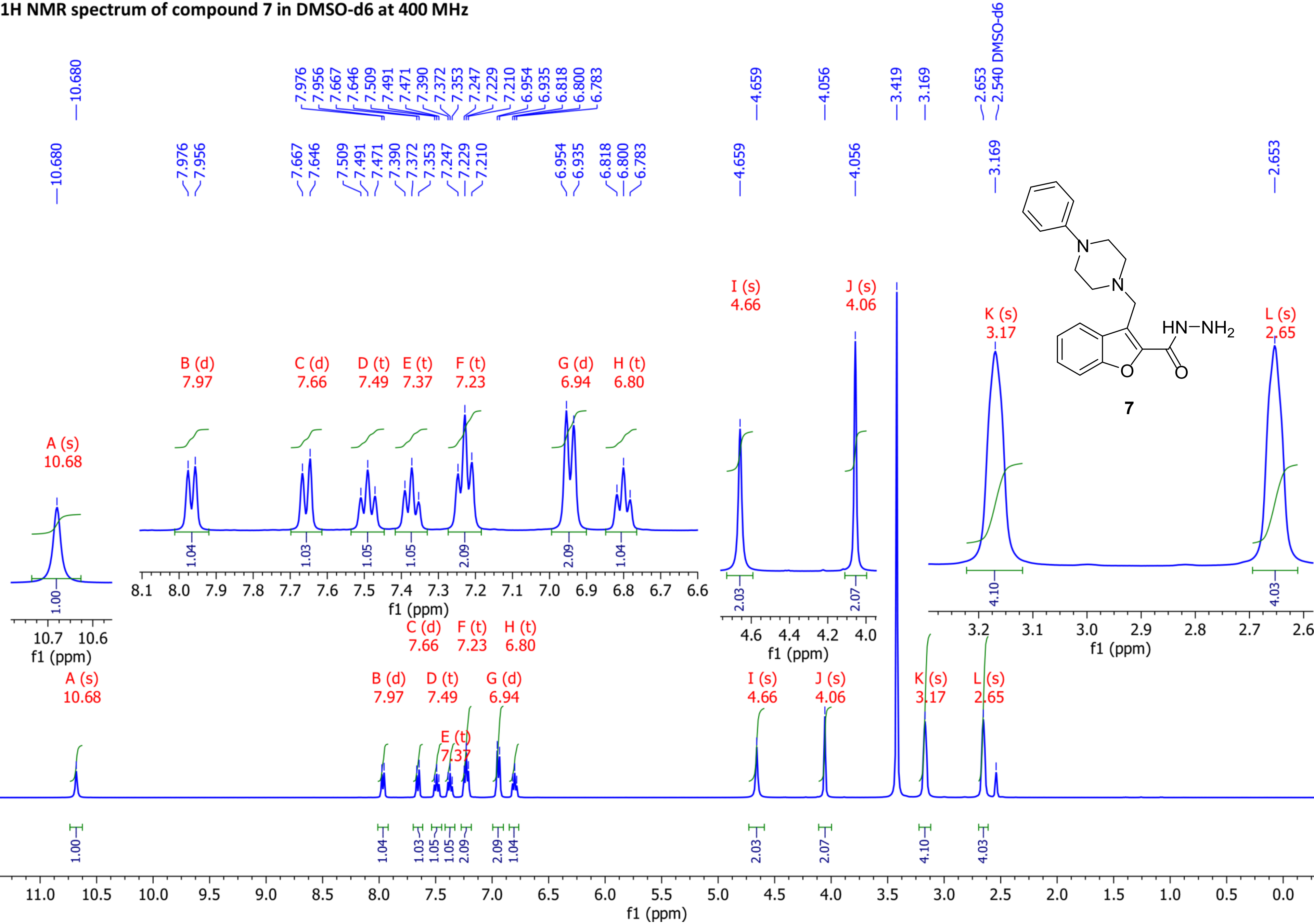

1H NMR spectrum of compound 9a in DMSO-d6 at 400 MHz

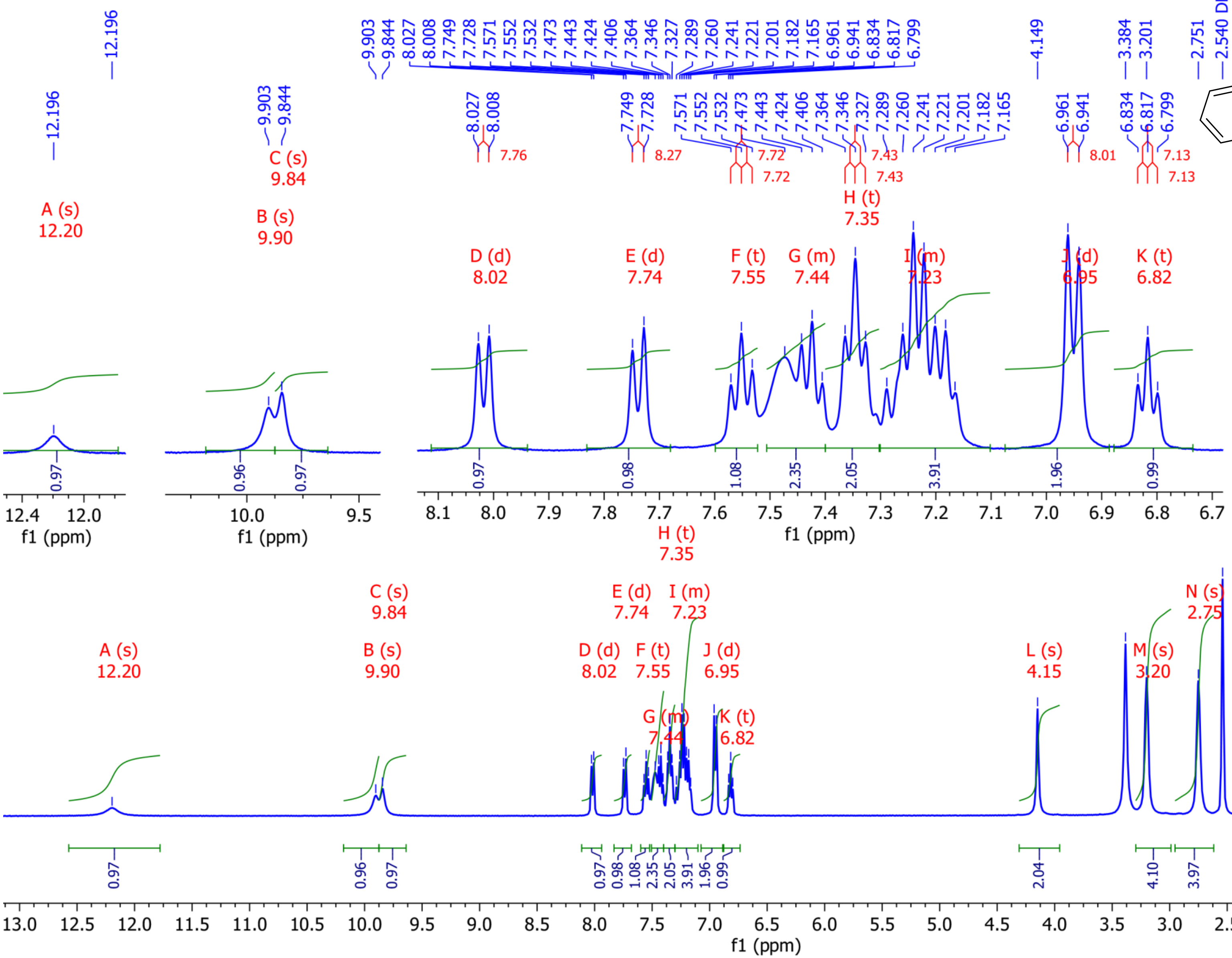

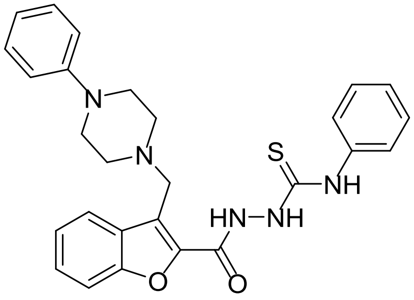

9a

13C NMR spectrum of compound 9a in DMSO-d6 at 400 MHz

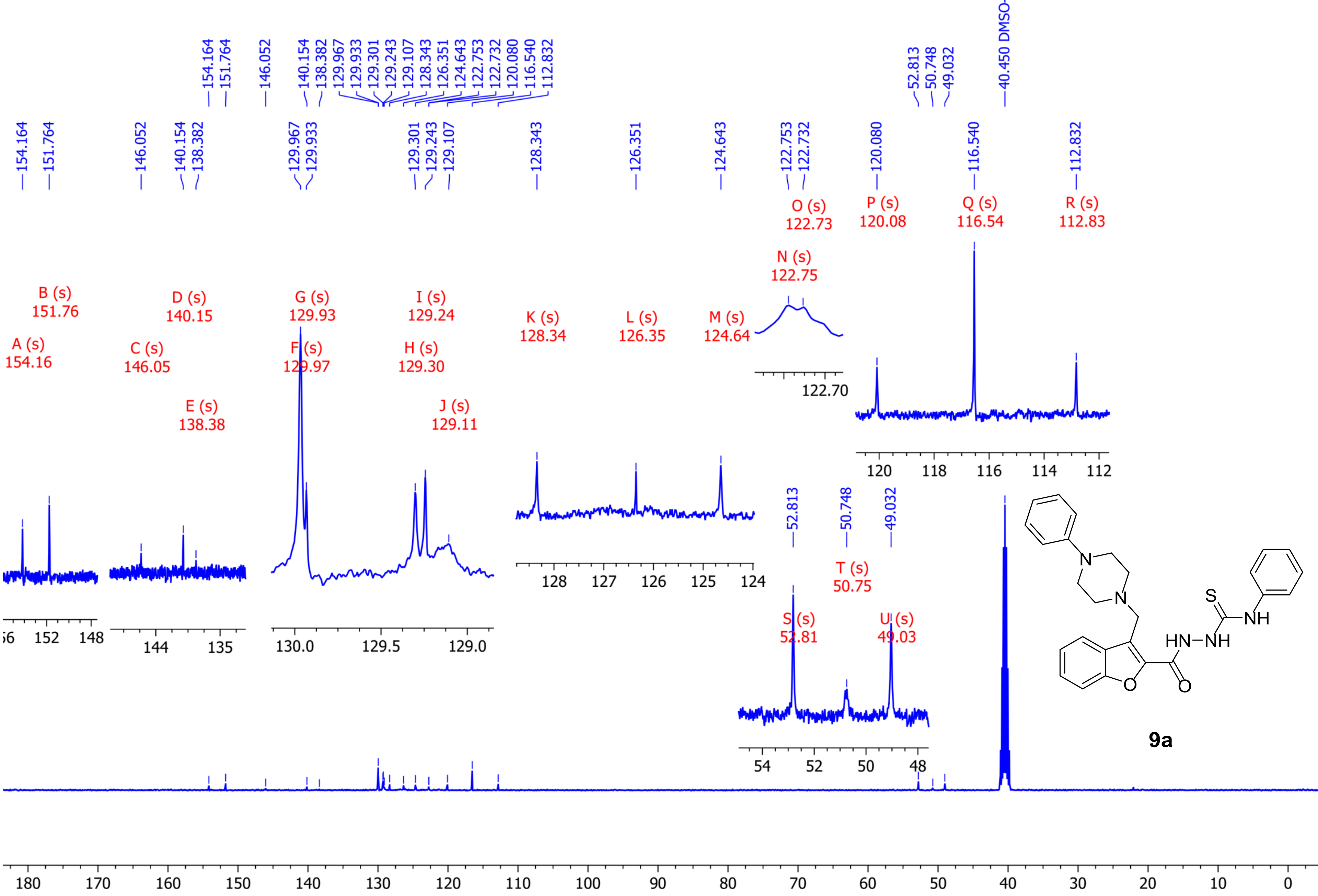

1H NMR spectrum of compound 9b in DMSO-d6 at 400 MHz

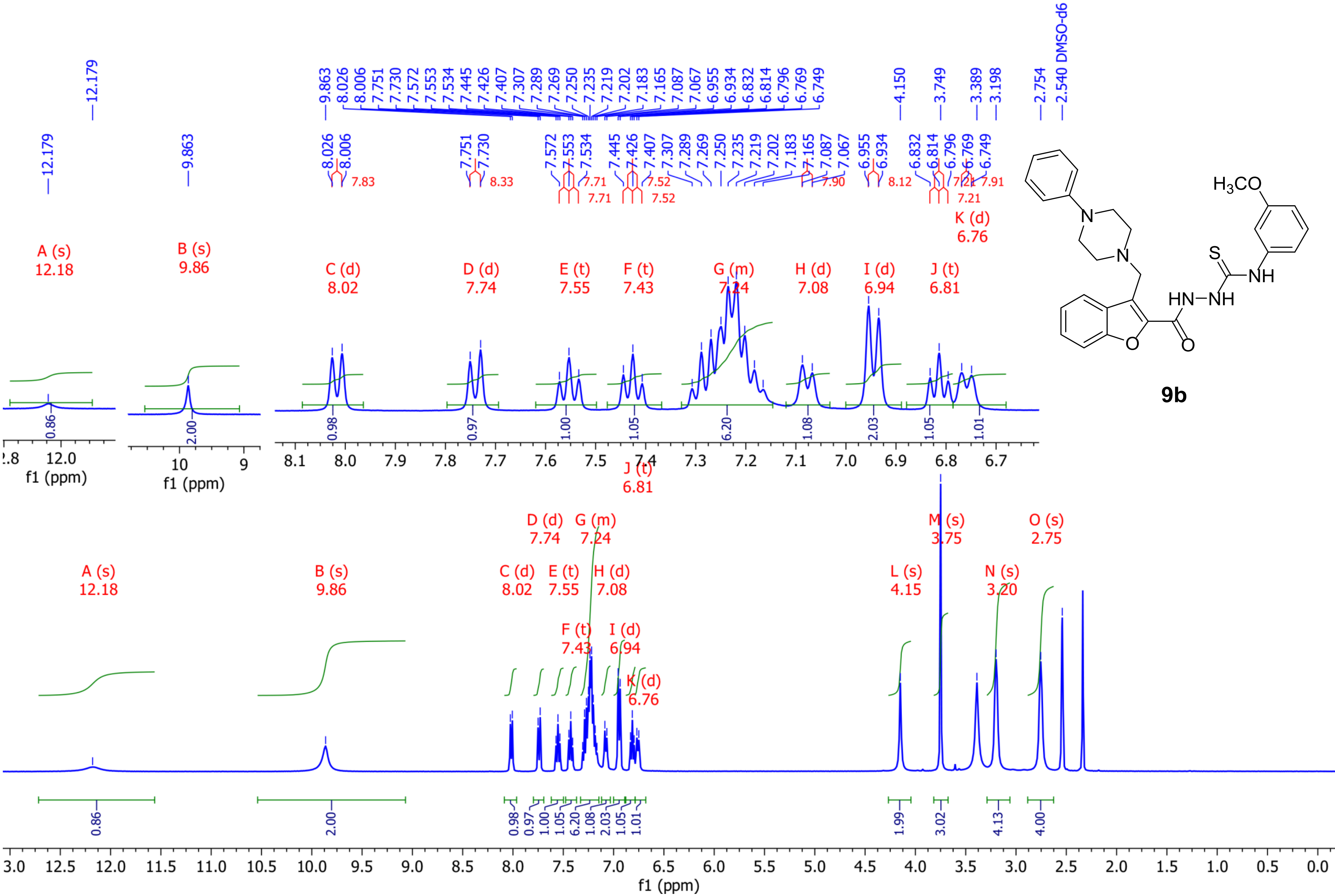

13C NMR spectrum of compound 9b in DMSO-d6 at 400 MHz

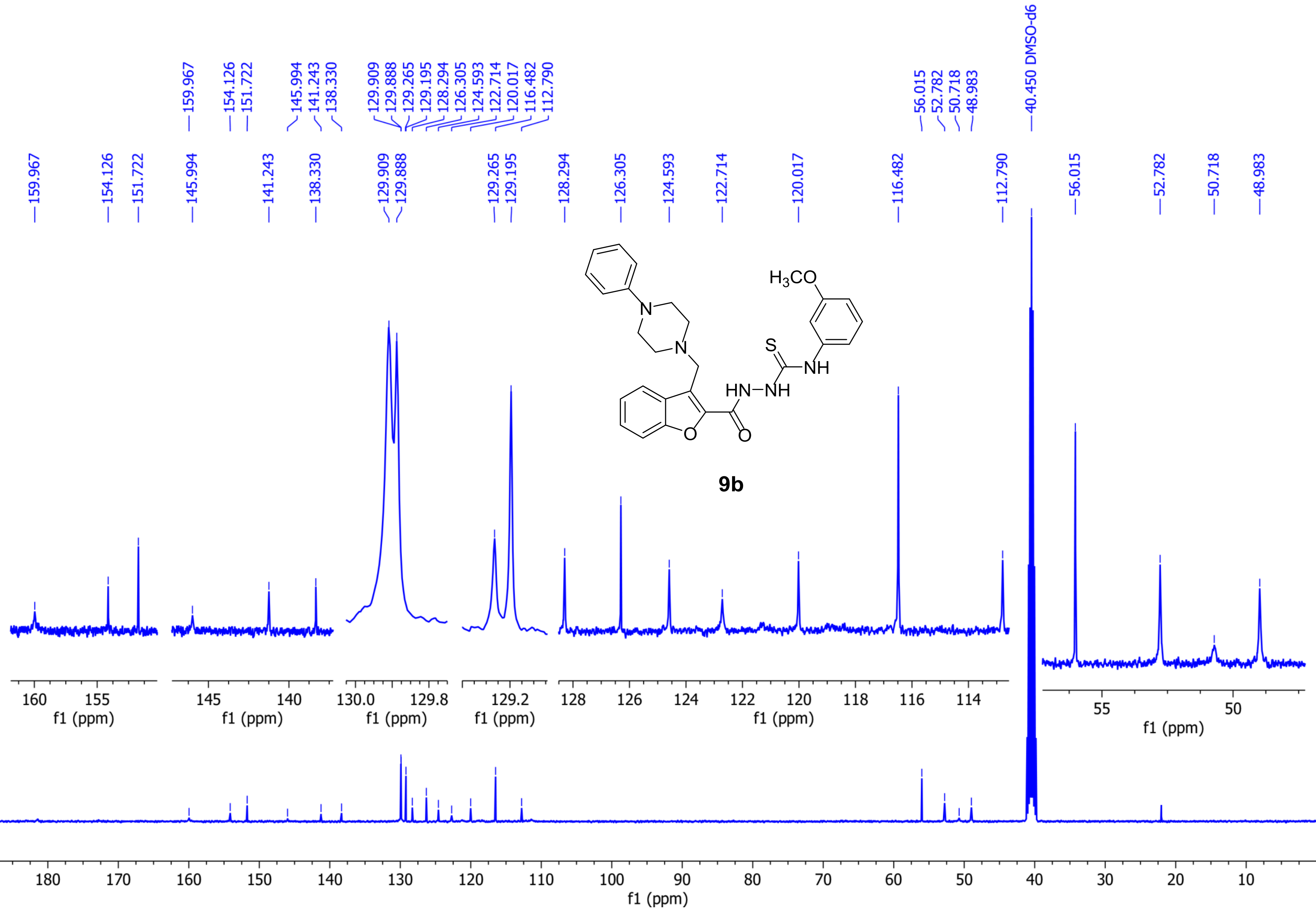

1H NMR spectrum of compound 9c in DMSO-d6 at 400 MHz

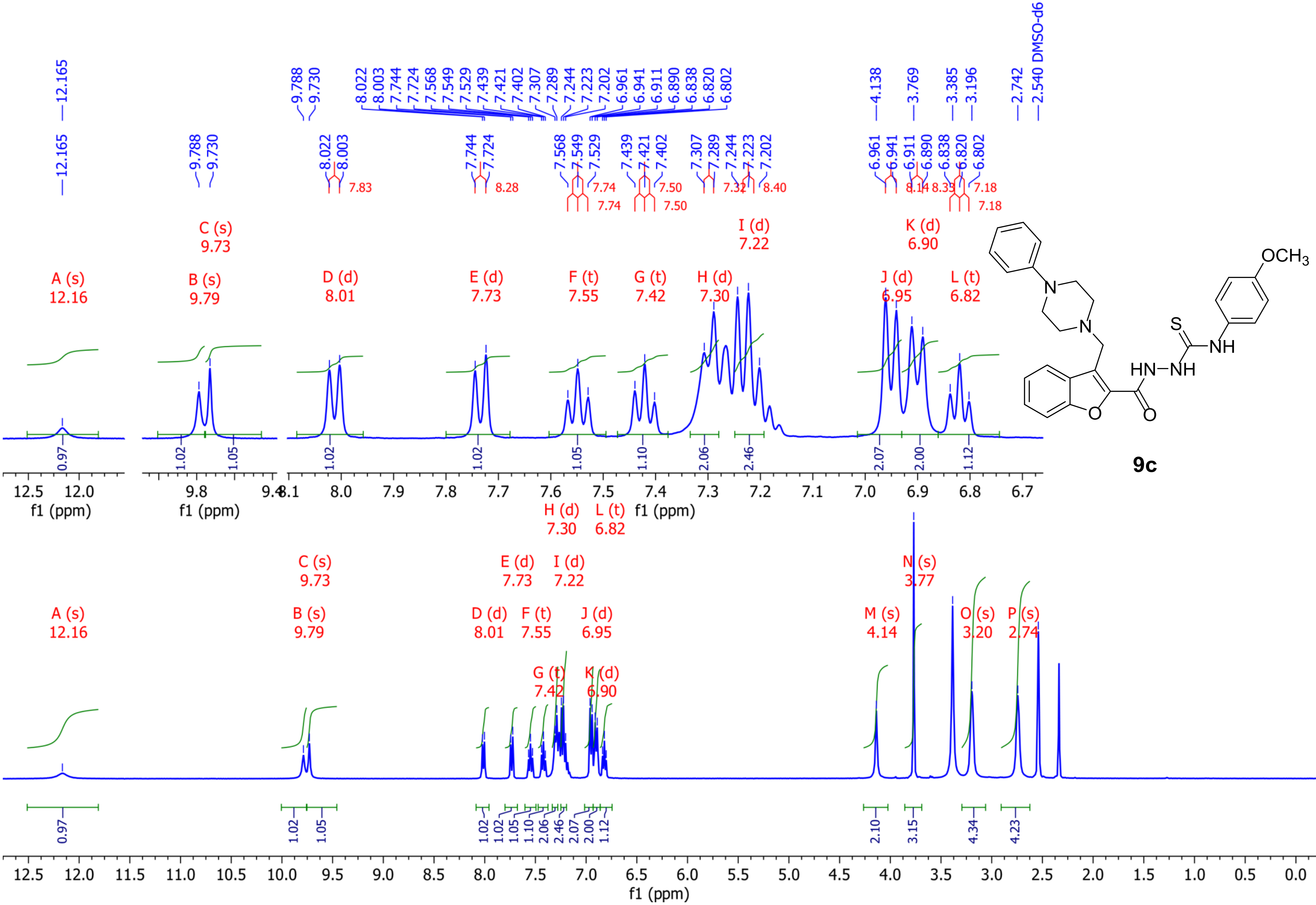

9c

13C NMR spectrum of compound 9c in DMSO-d6 at 400 MHz

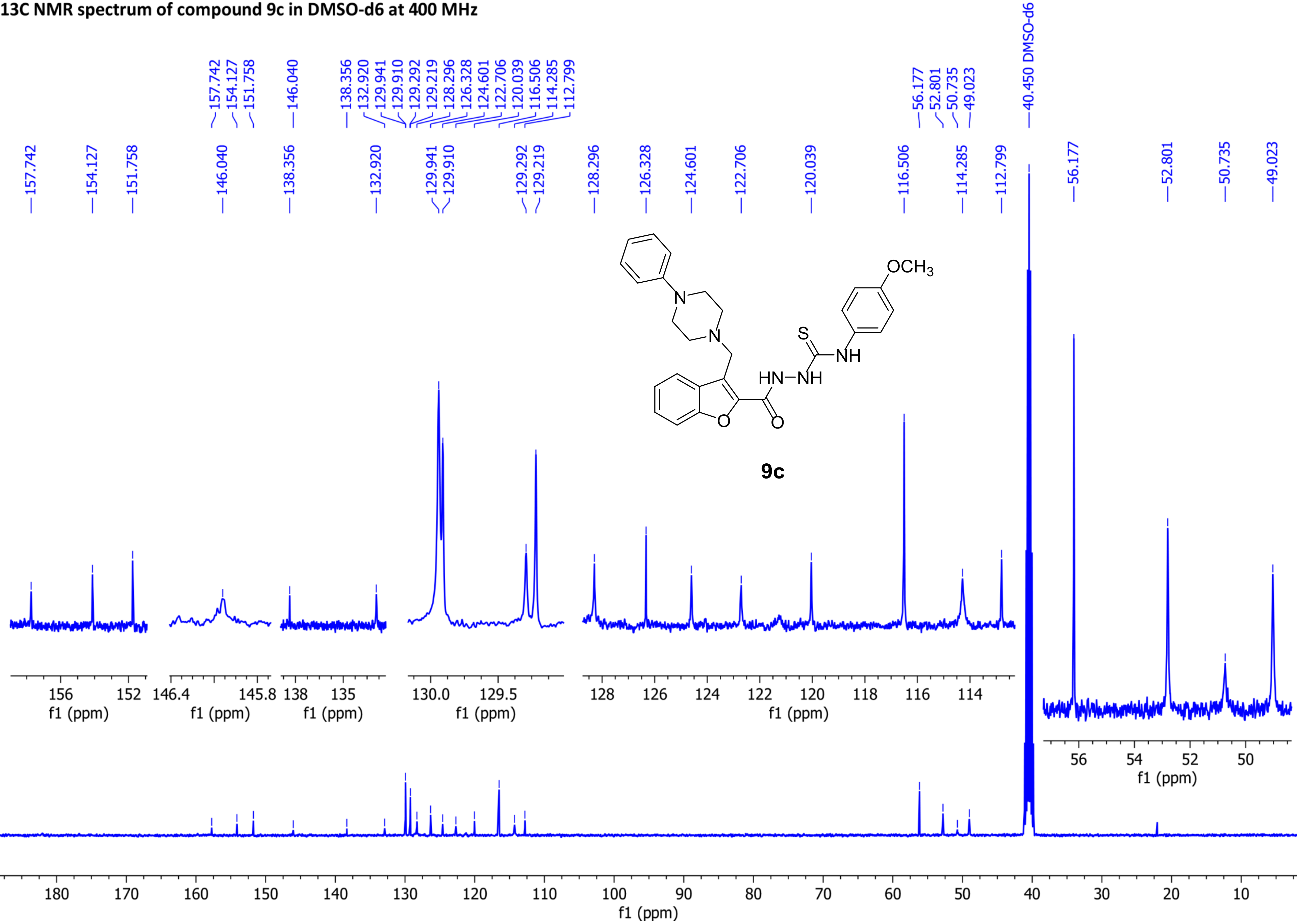

1H NMR spectrum of compound 9d in DMSO-d6 at 400 MHz

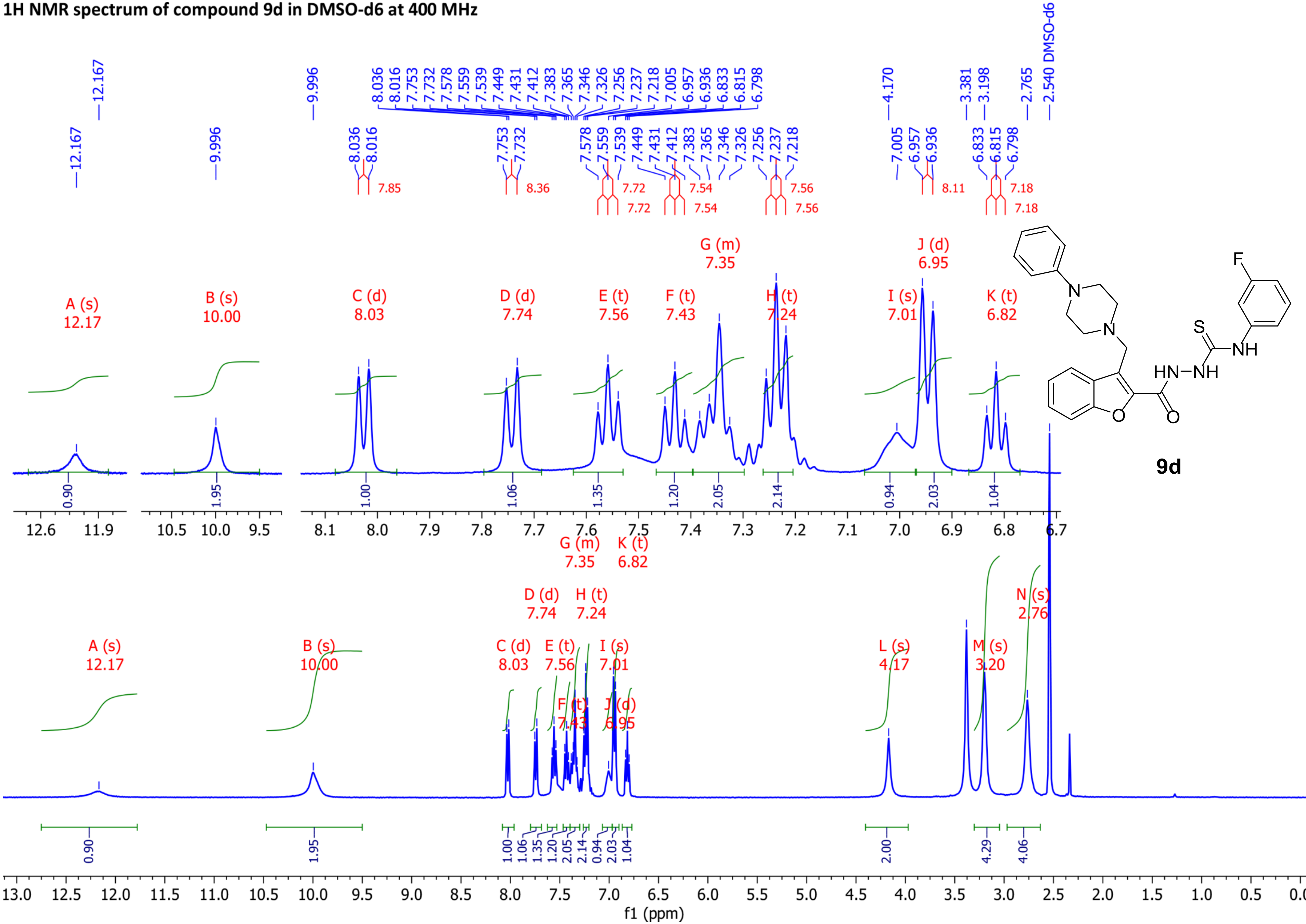

13C NMR spectrum of compound 9d in DMSO-d6 at 400 MHz

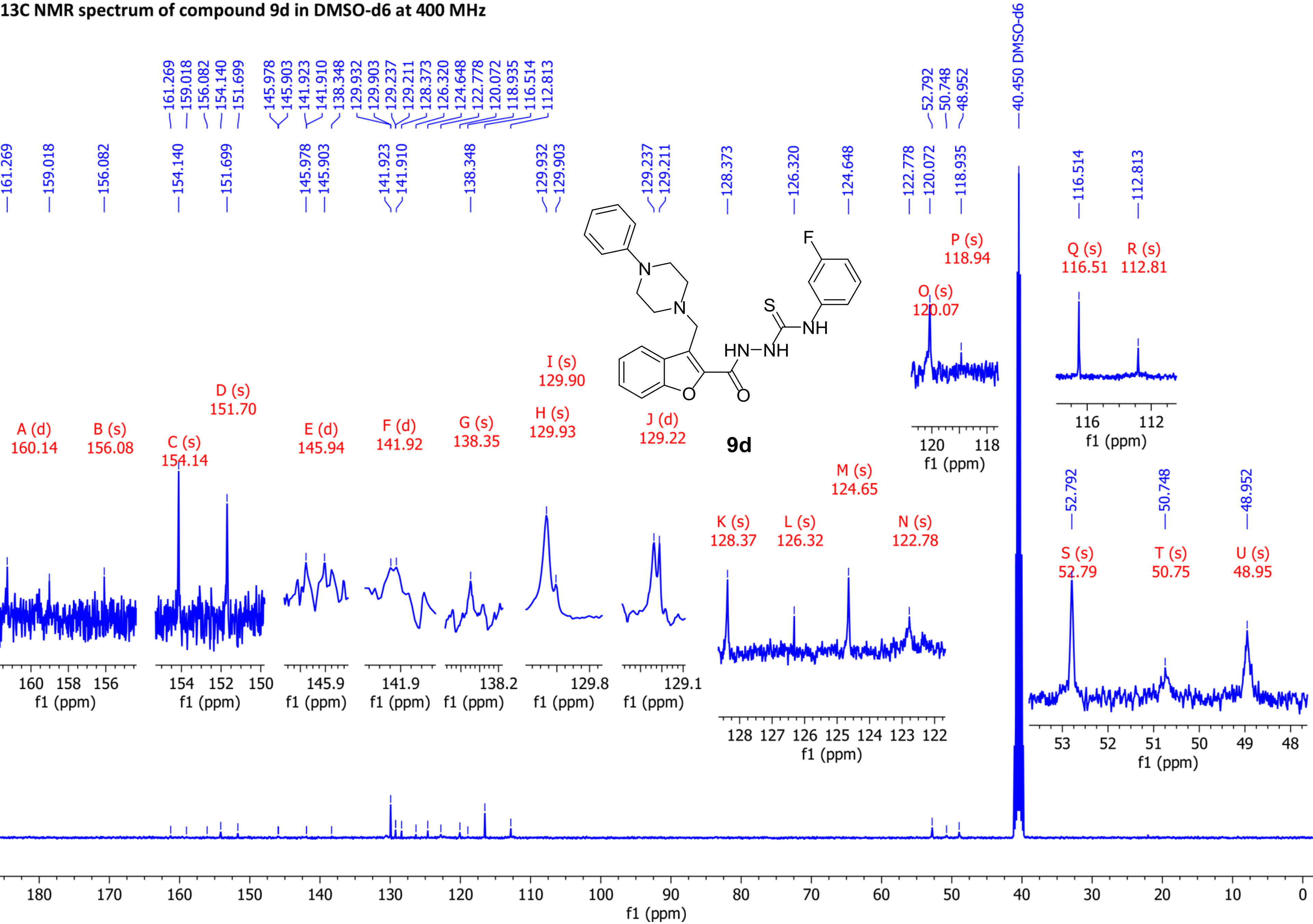

1H NMR spectrum of compound 9e in DMSO-d6 at 400 MHz

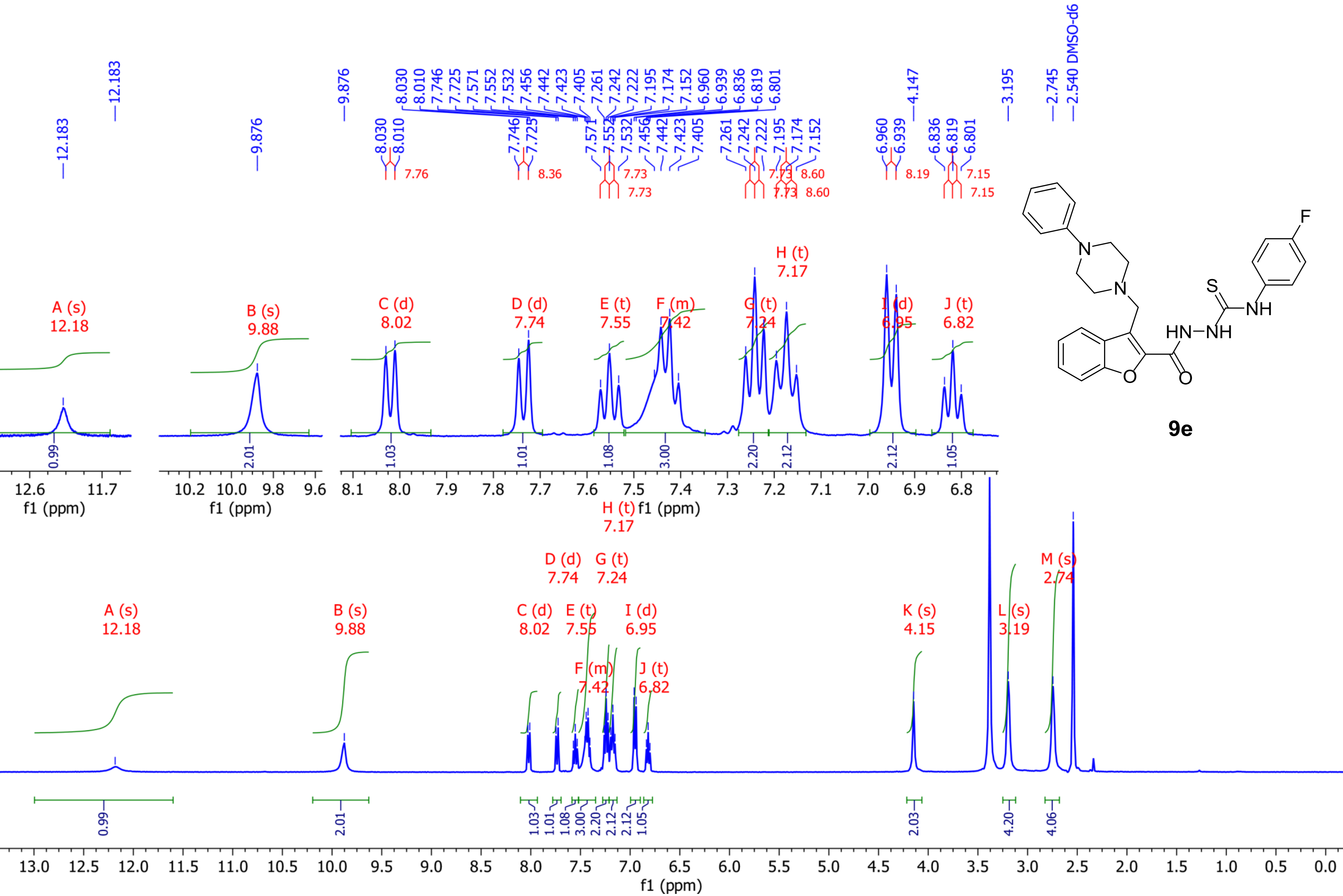

13C NMR spectrum of compound 9e in DMSO-d6 at 400 MHz

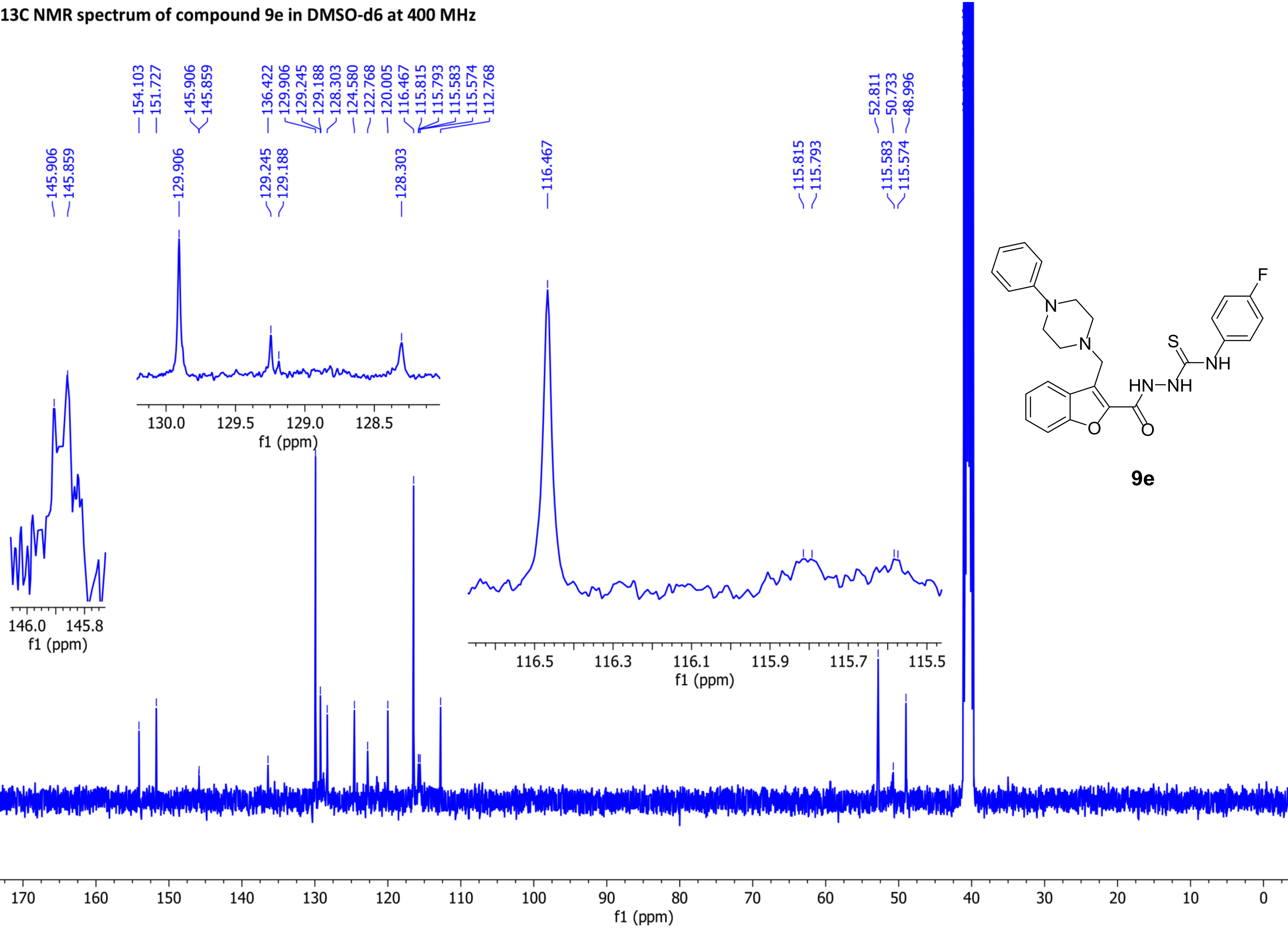

1H NMR spectrum of compound 9f in DMSO-d6 at 400 MHz

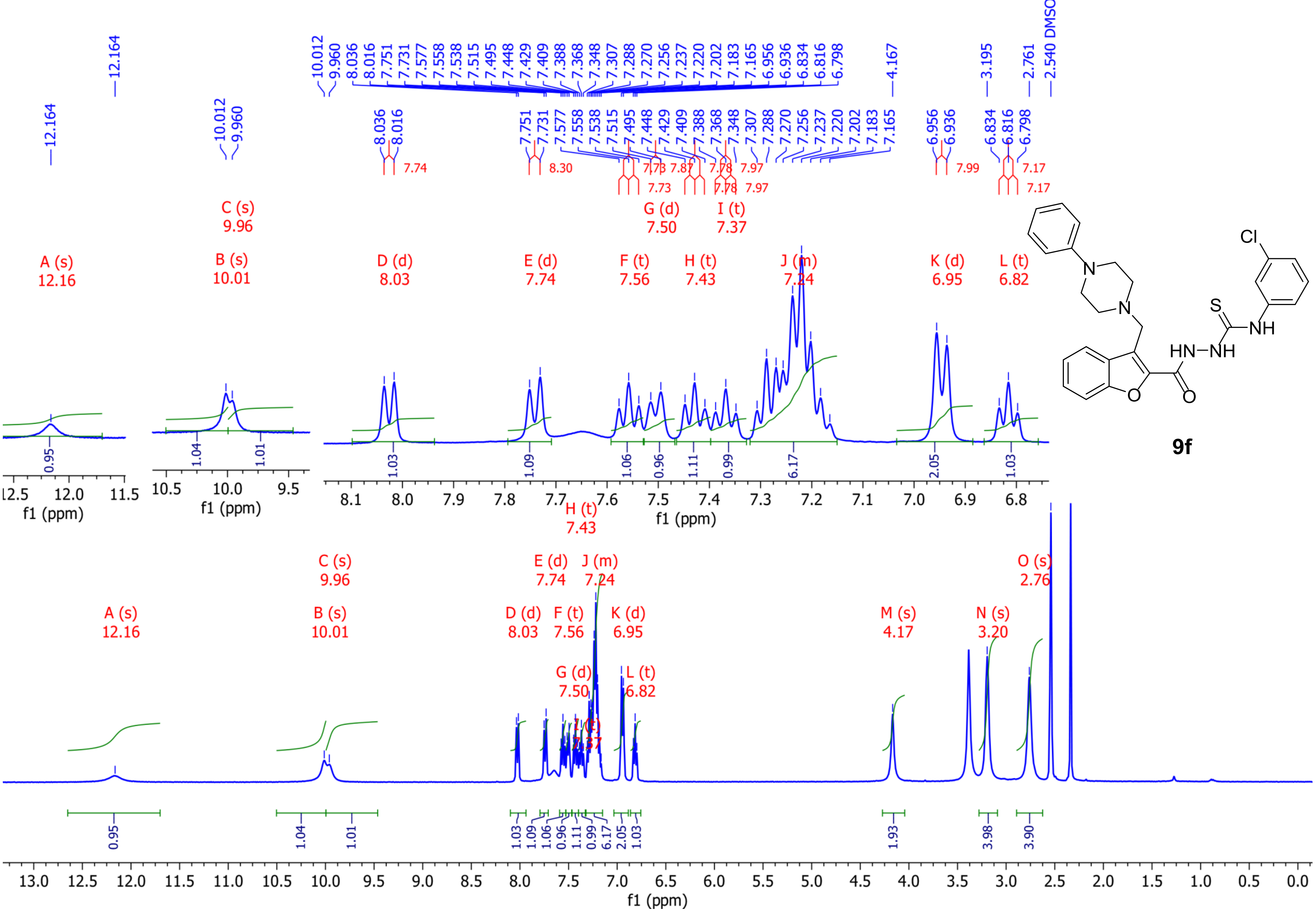

13C NMR spectrum of compound 9f in DMSO-d6 at 400 MHz

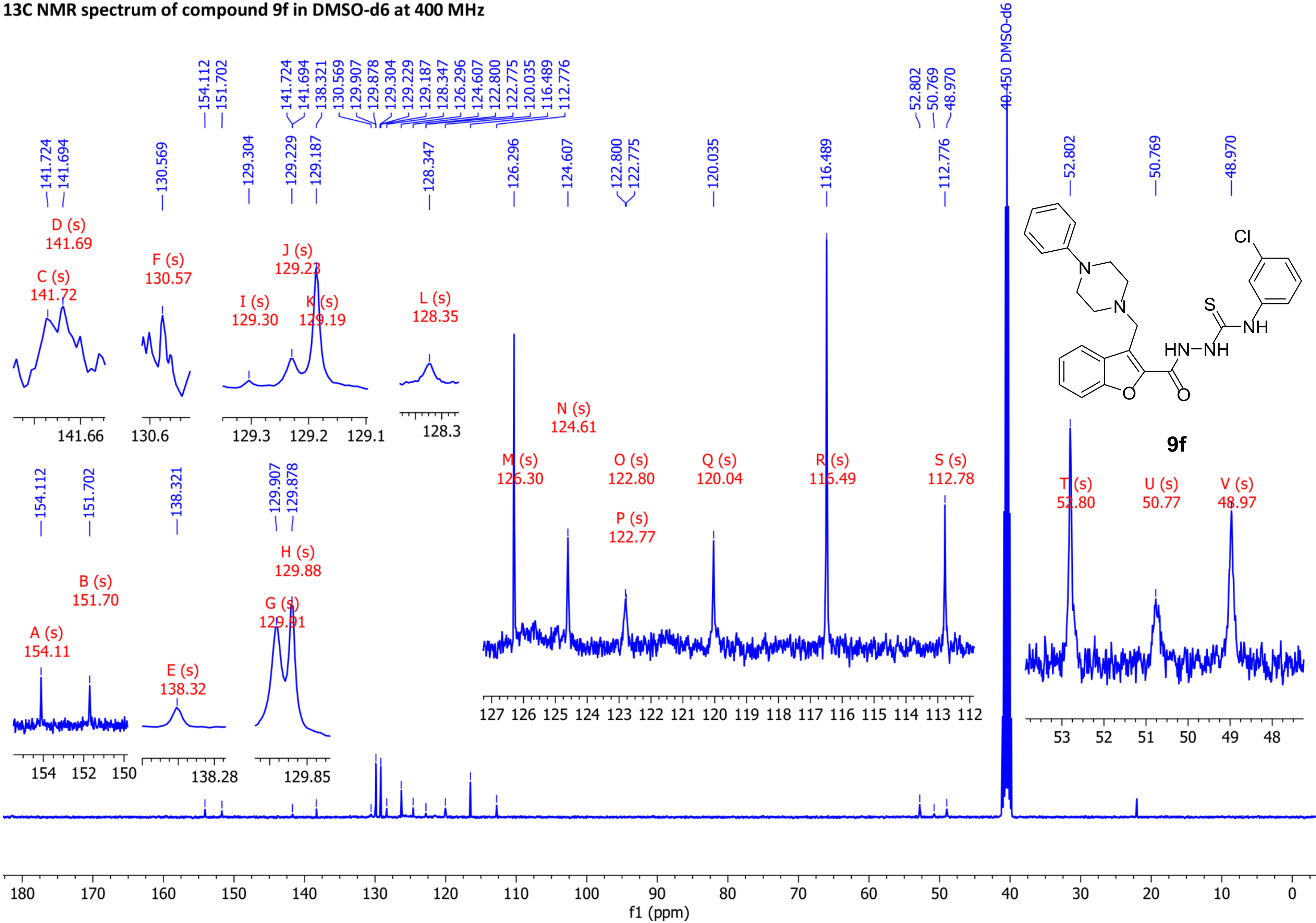

1H NMR spectrum of compound 9g in DMSO-d6 at 400 MHz

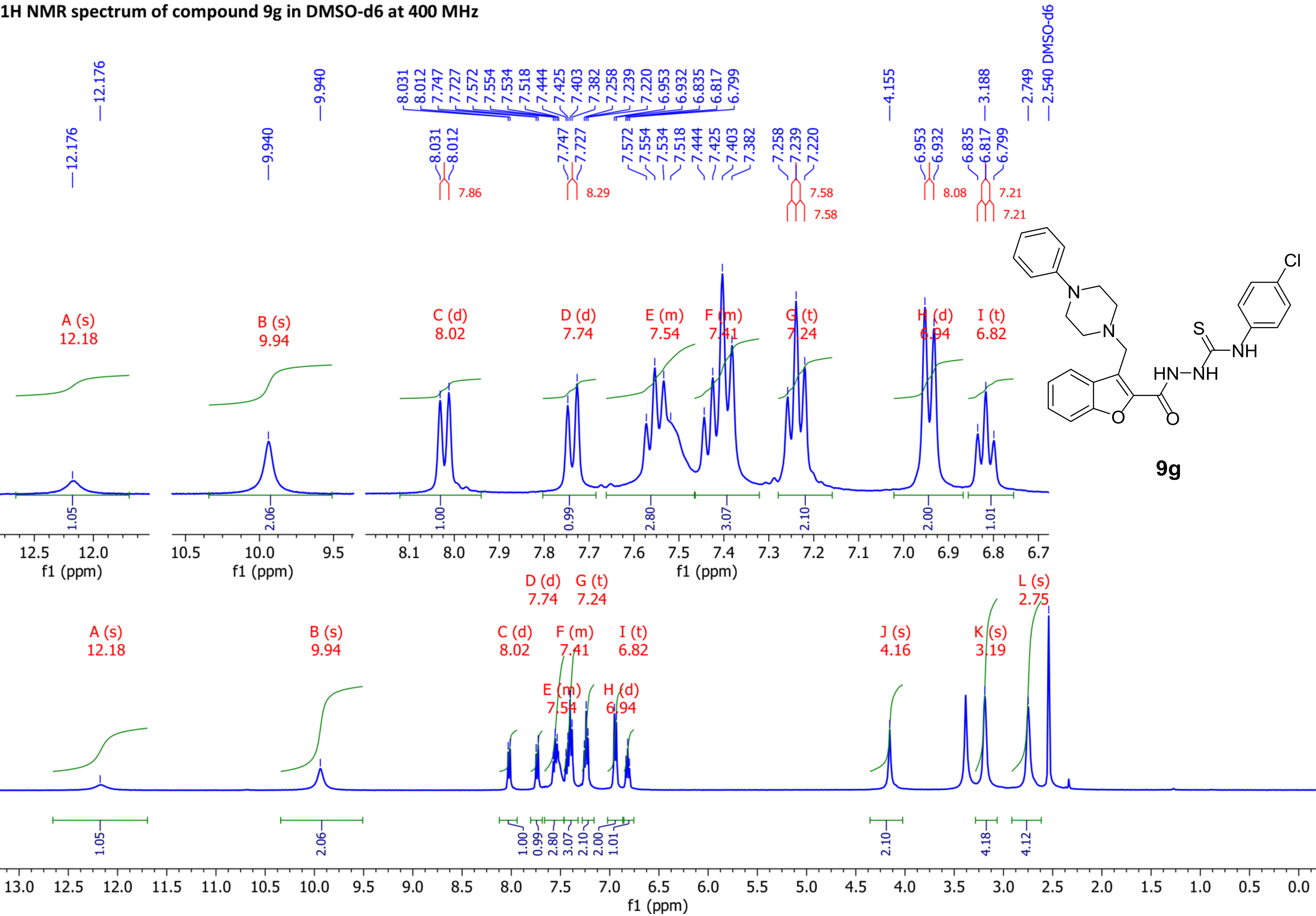

13C NMR spectrum of compound 9g in DMSO-d6 at 400 MHz

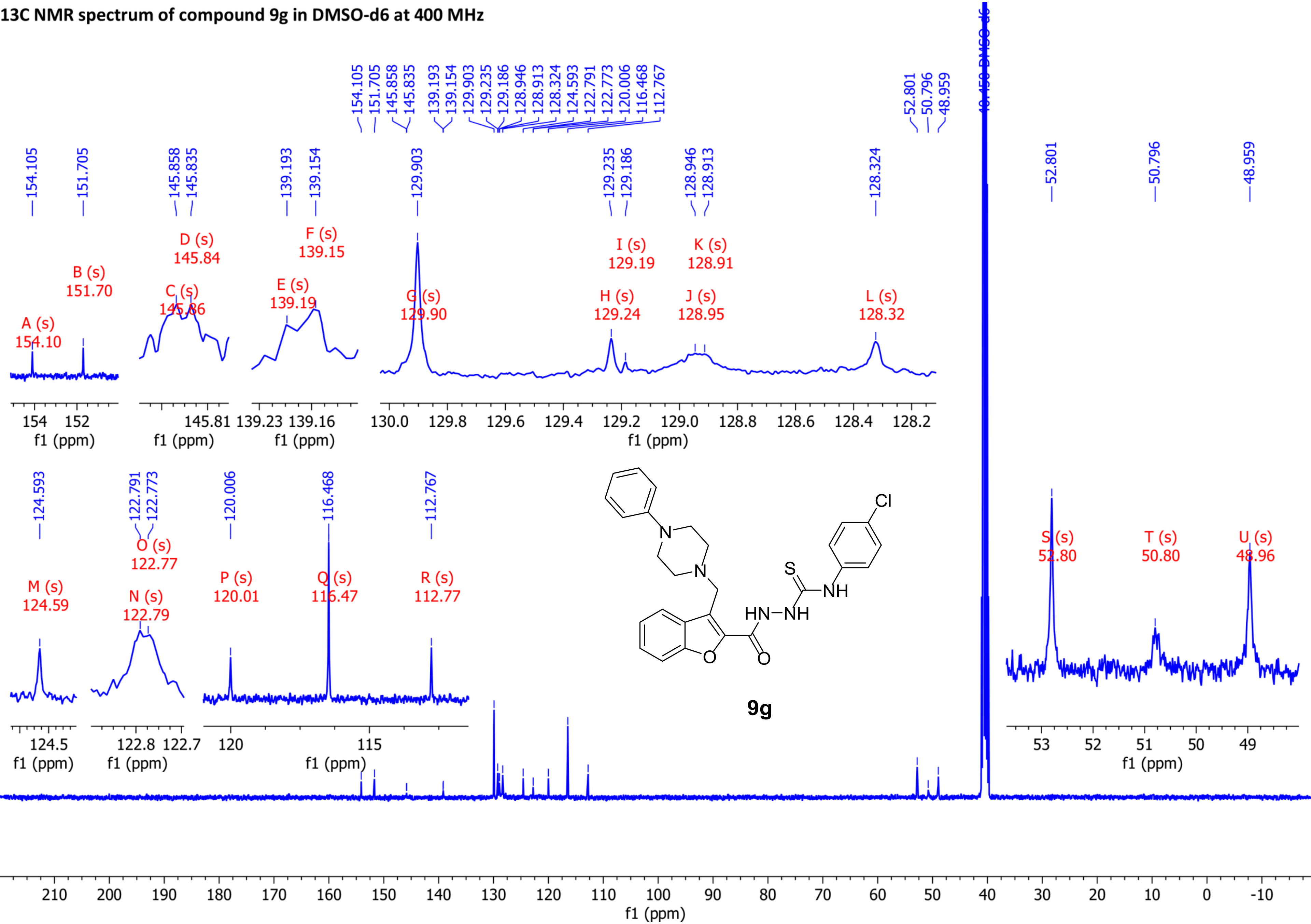

1H NMR spectrum of compound 9h in DMSO-d6 at 400 MHz

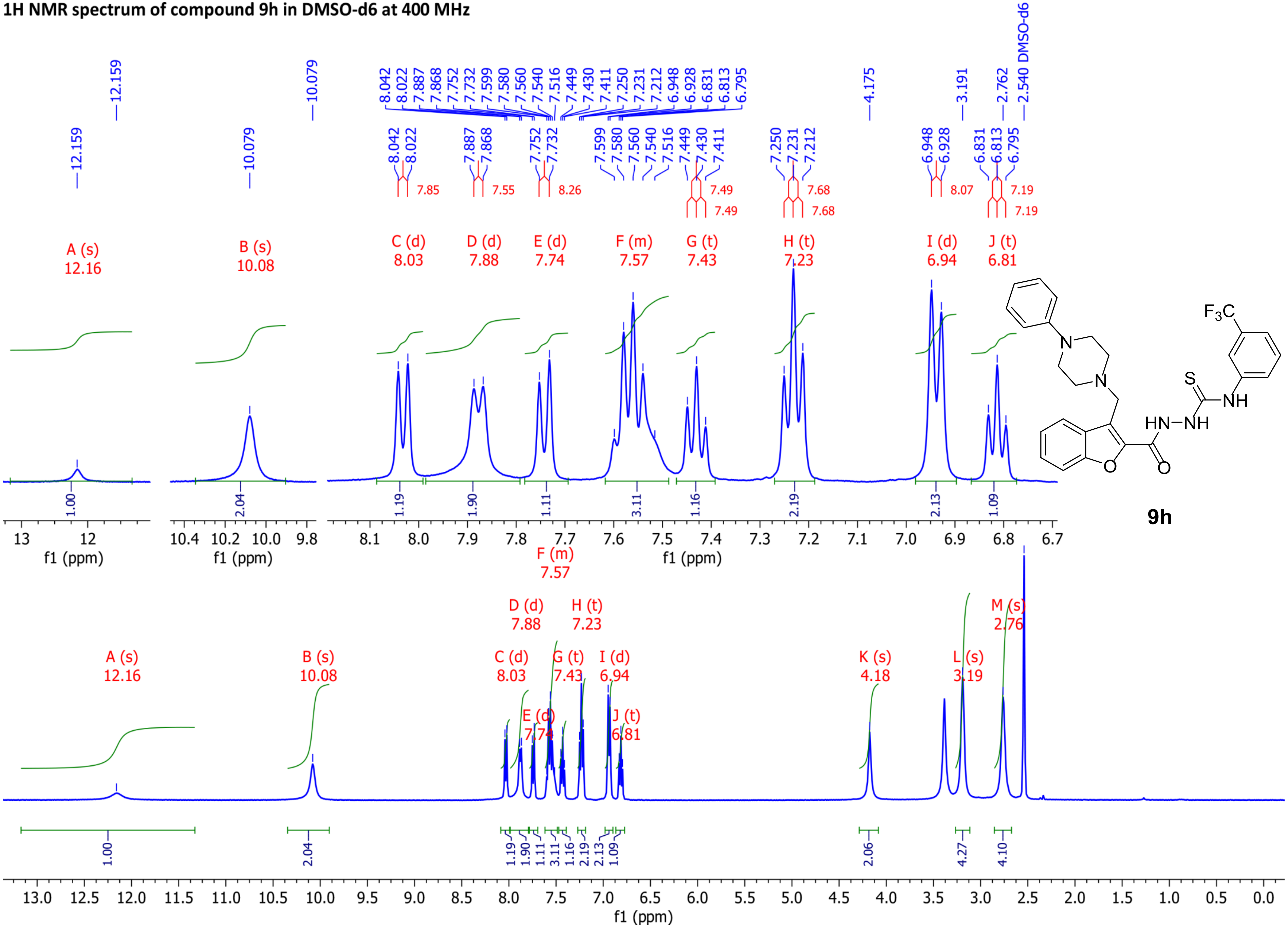

13C NMR spectrum of compound 9h in DMSO-d6 at 400 MHz

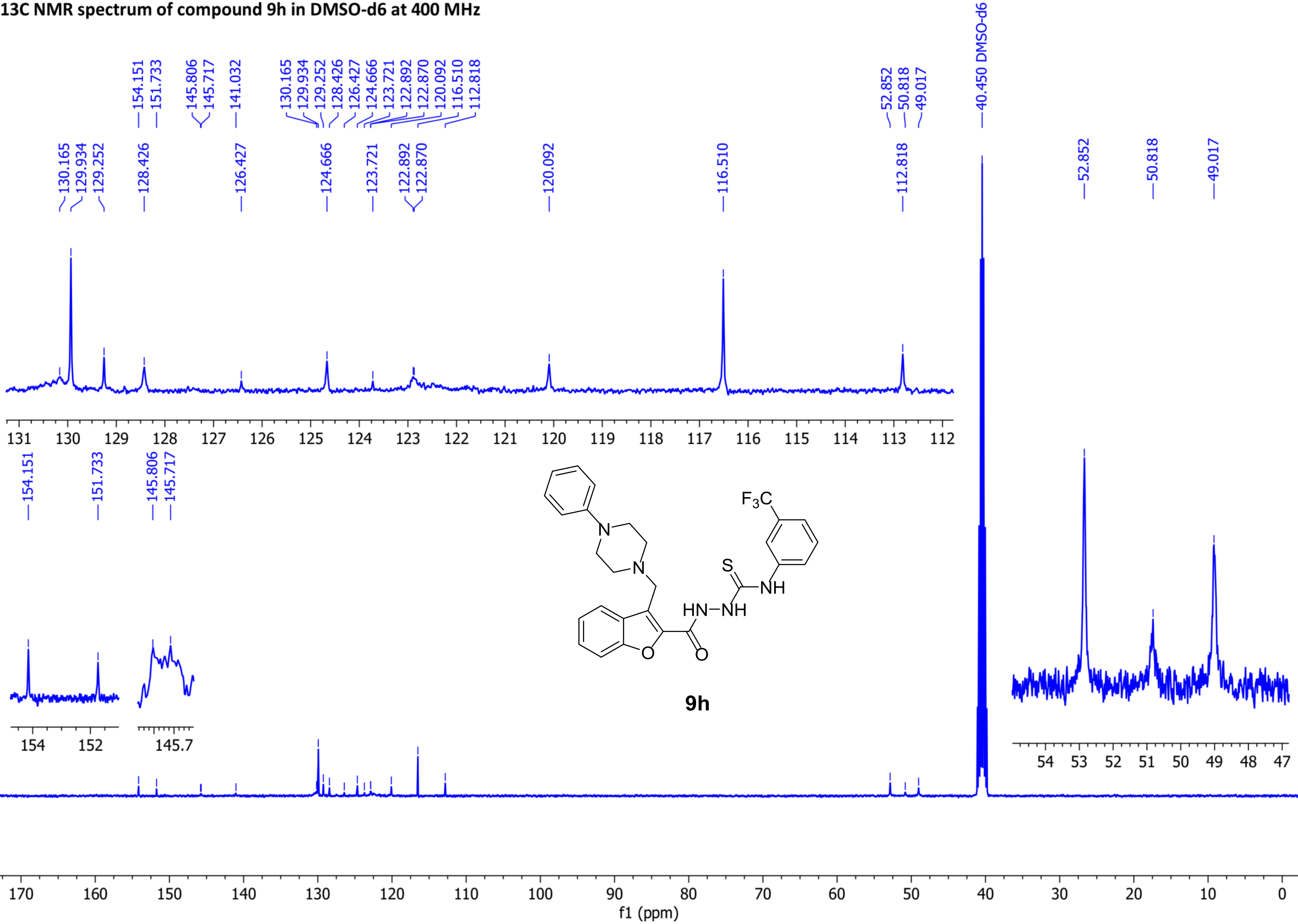

1H NMR spectrum of compound 9i in DMSO-d6 at 400 MHz

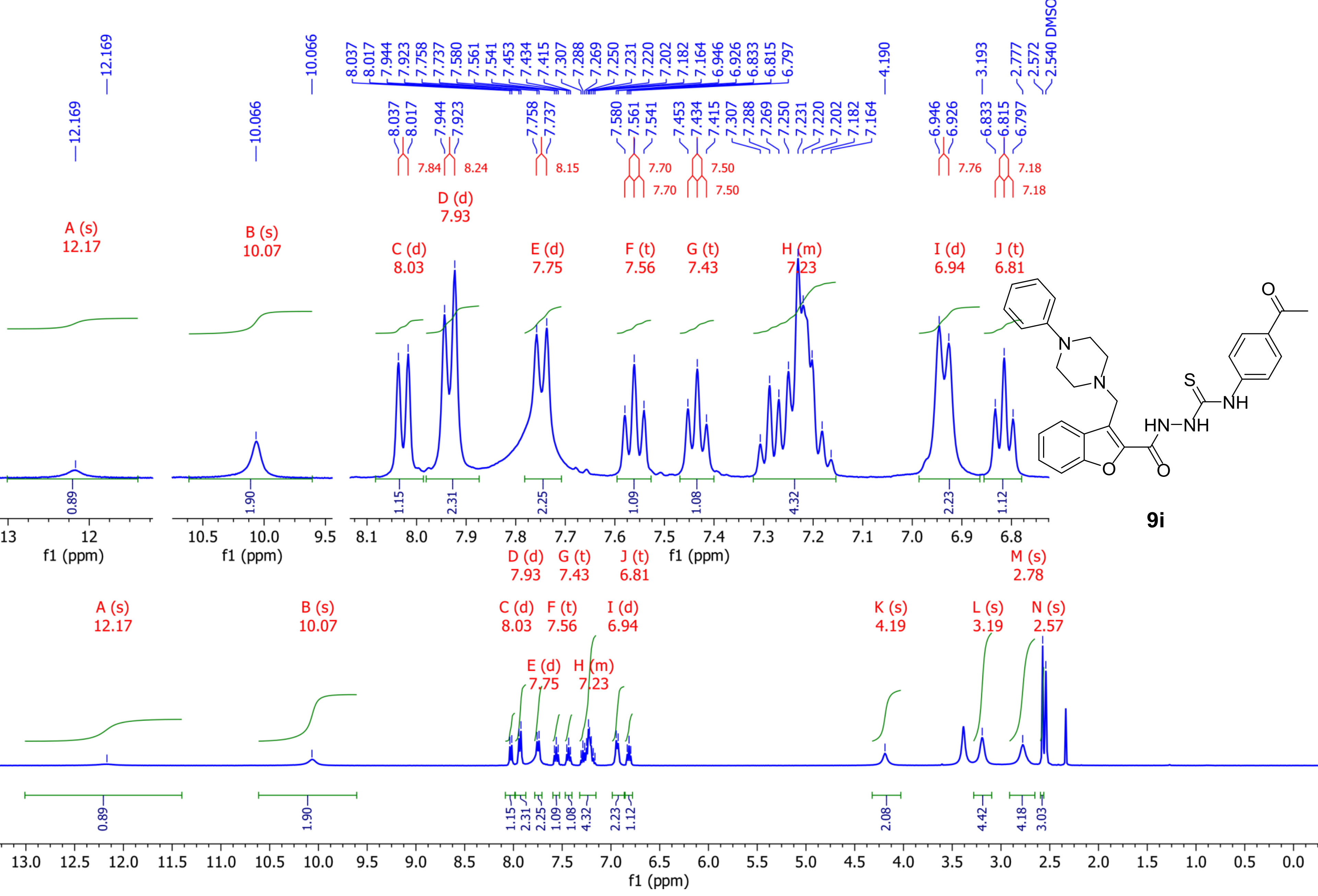

13C NMR spectrum of compound 9i in DMSO-d6 at 400 MHz

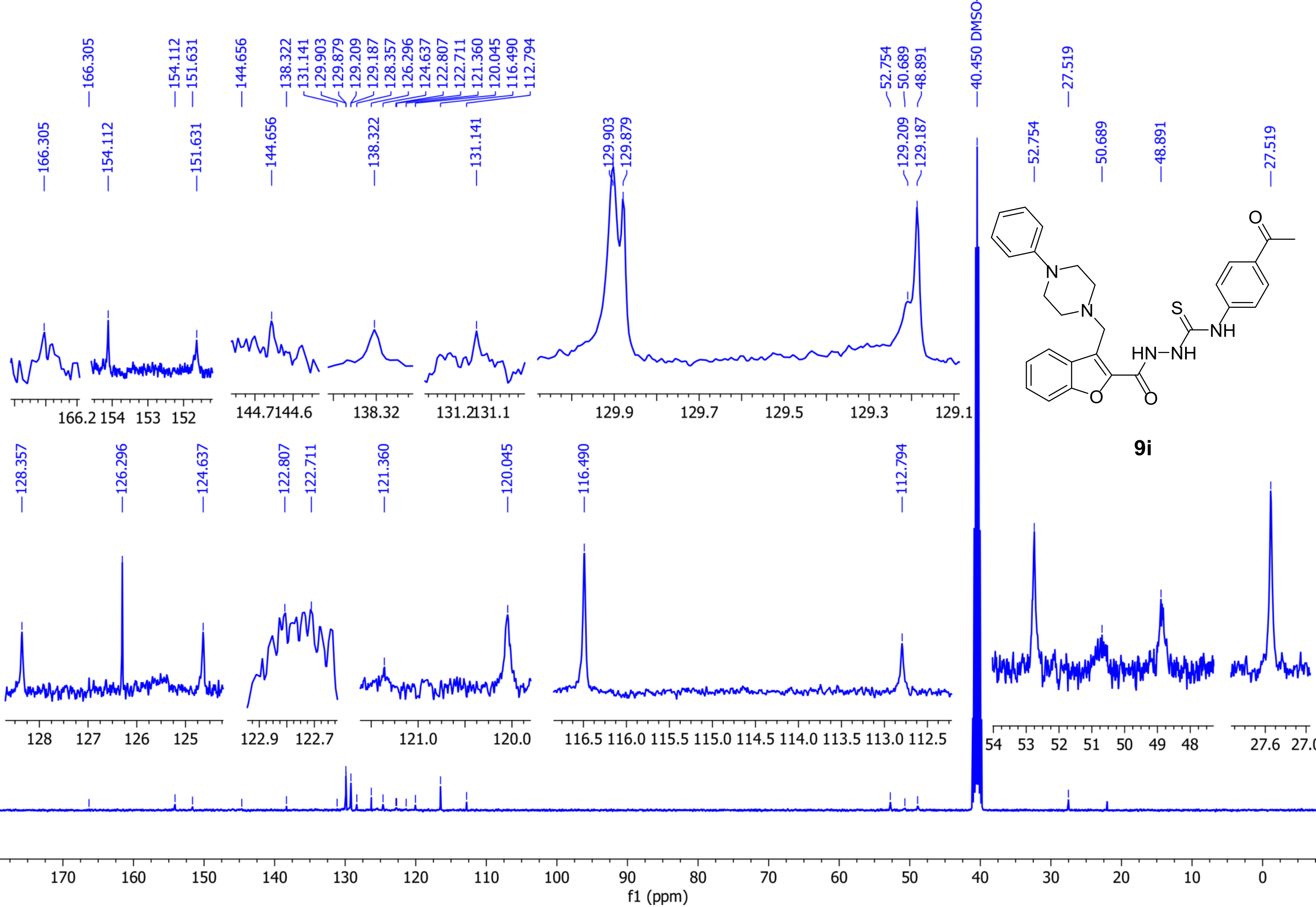

1H NMR spectrum of compound 11a in DMSO-d6 at 400 MHz

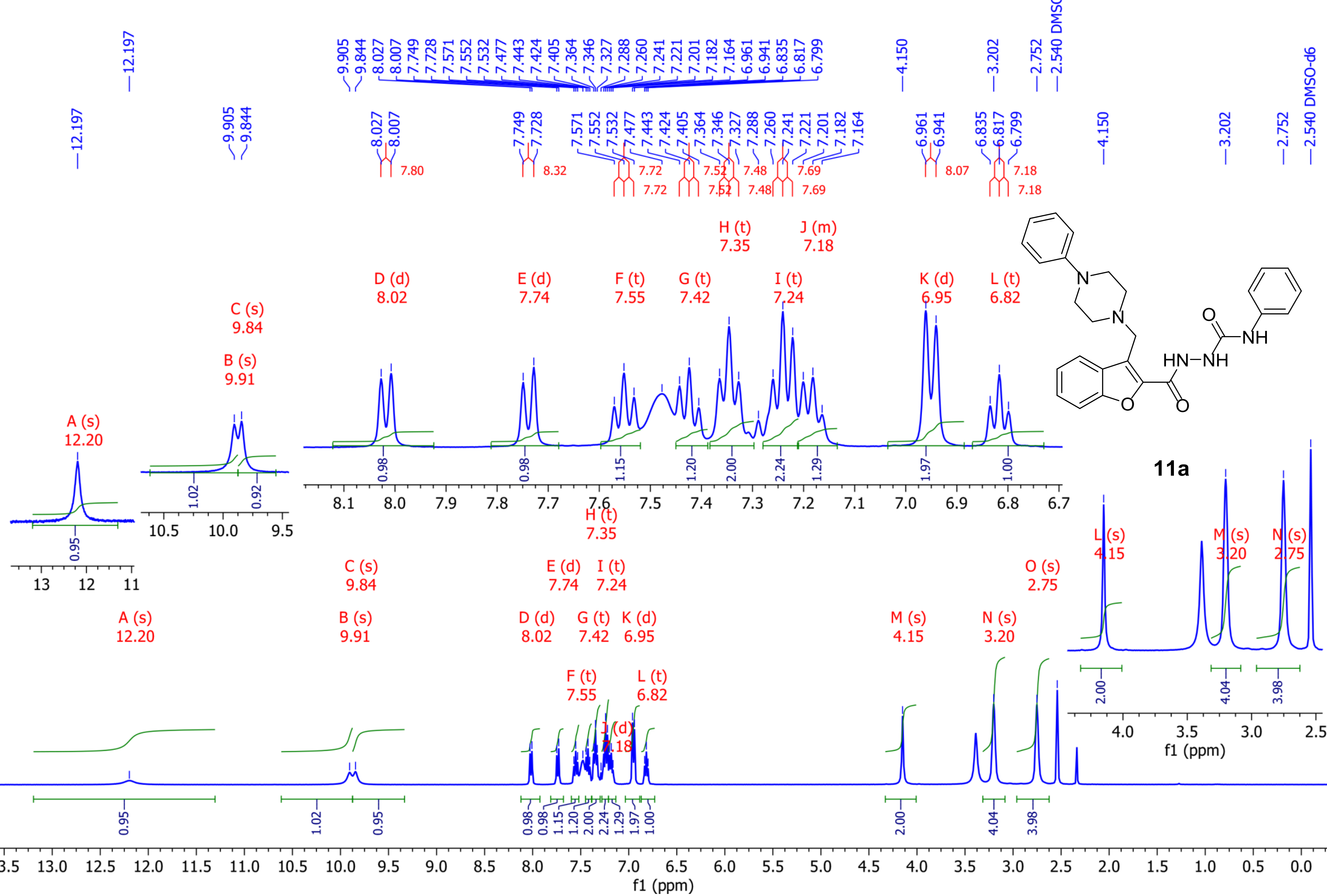

13C NMR spectrum of compound 11a in DMSO-d6 at 400 MHz

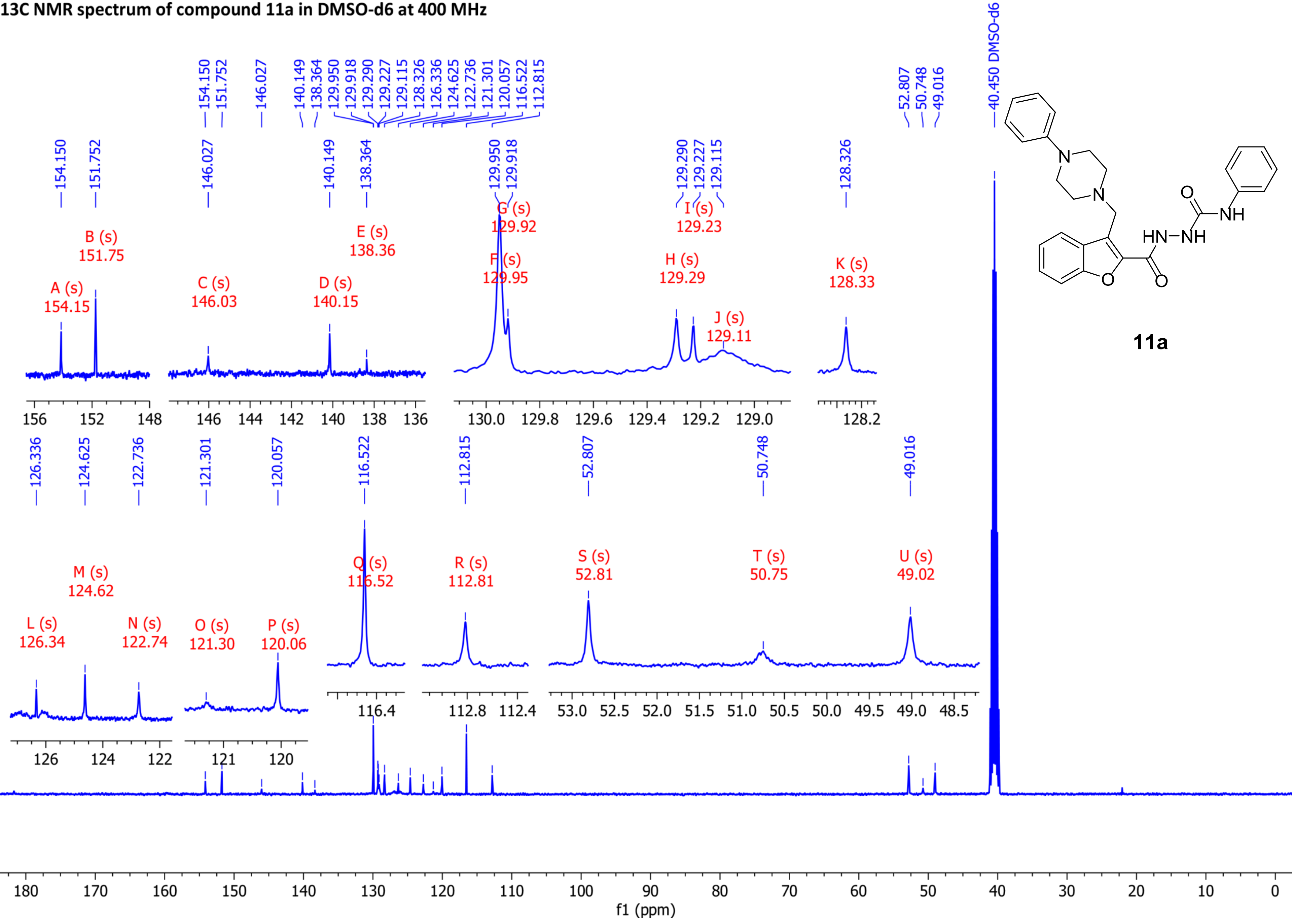

1H NMR spectrum of compound 11b in DMSO-d6 at 400 MHz

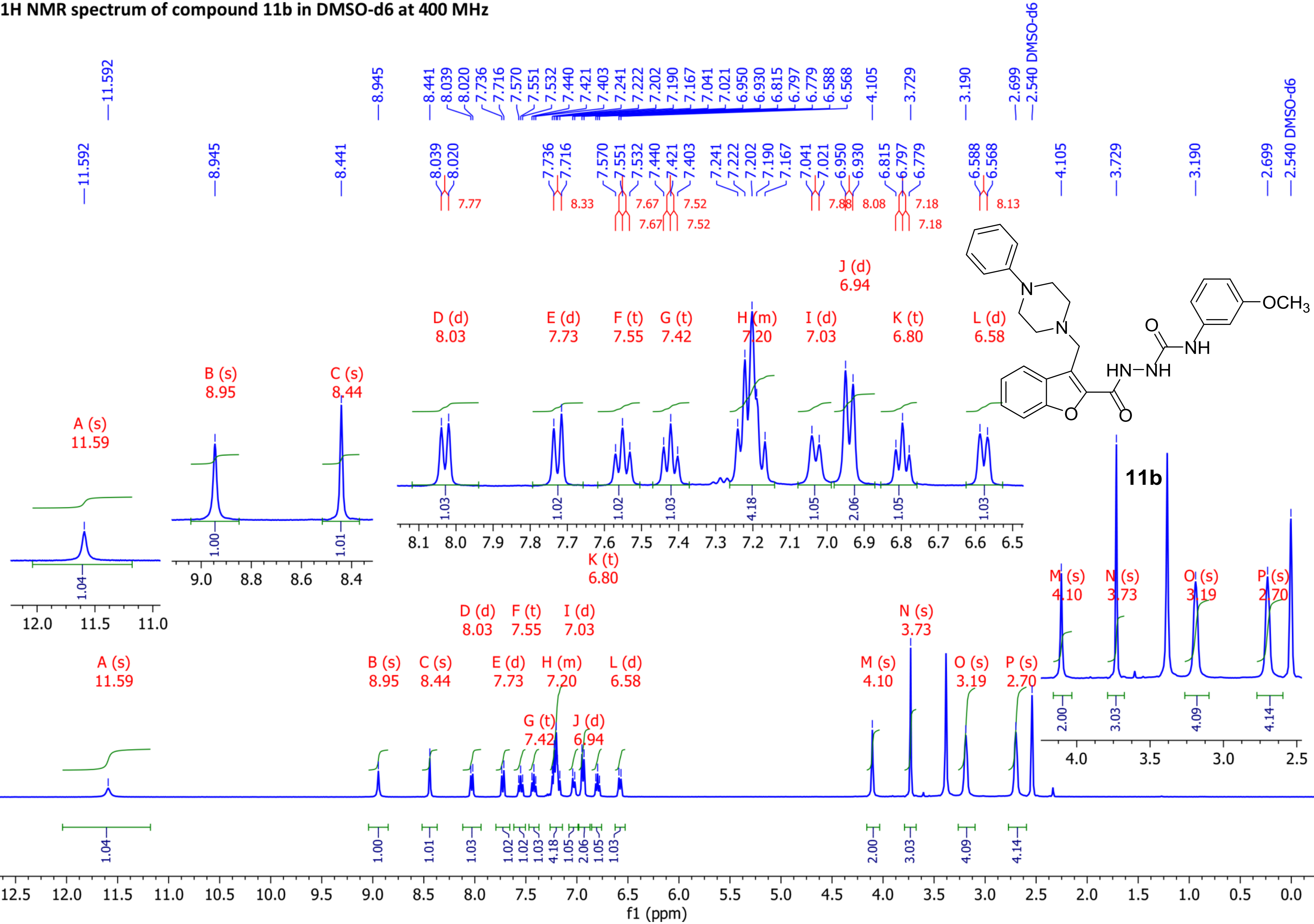

13C NMR spectrum of compound 11b in DMSO-d6 at 400 MHz

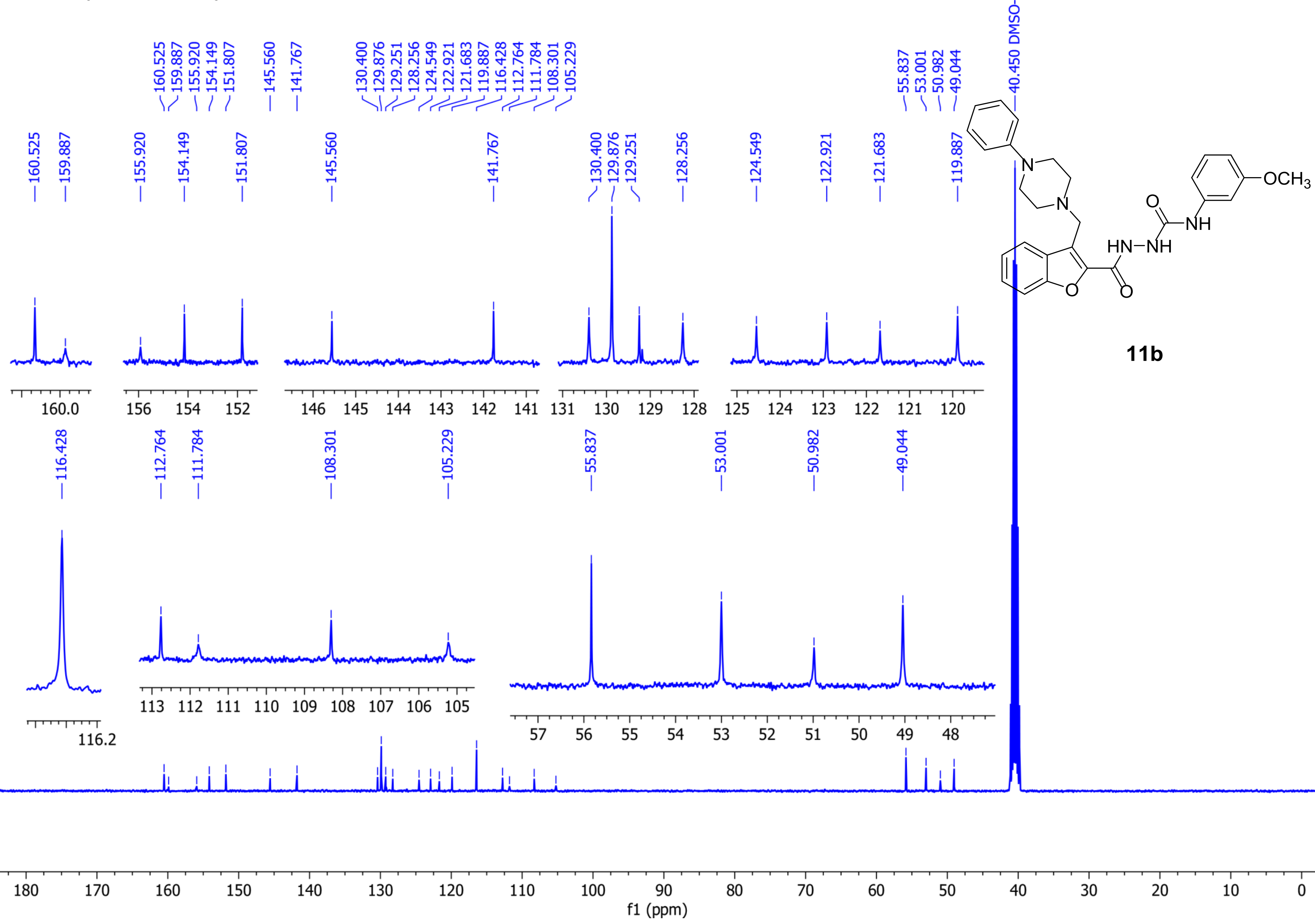

1H NMR spectrum of compound 11c in DMSO-d6 at 400 MHz

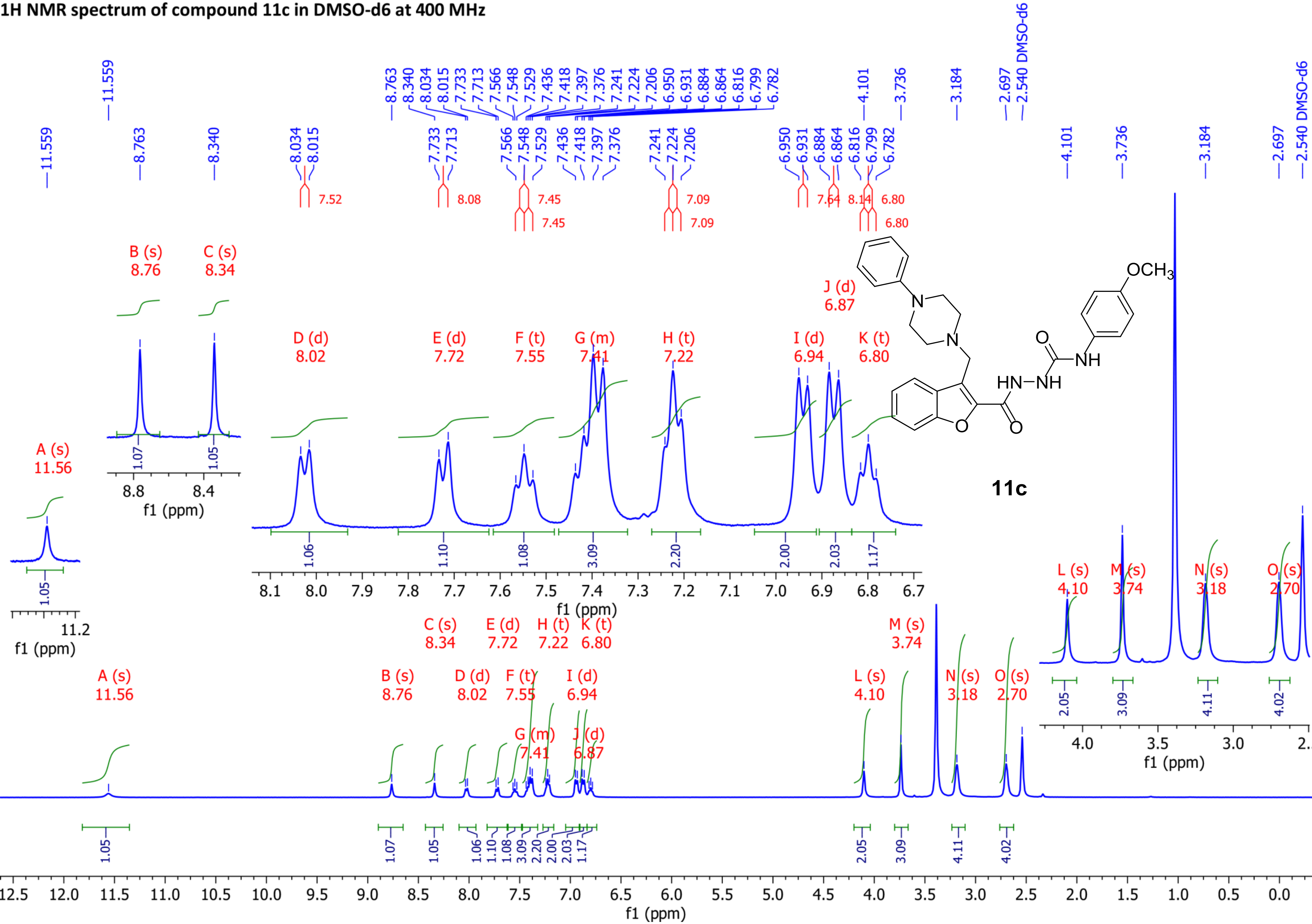

13C NMR spectrum of compound 11c in DMSO-d6 at 400 MHz

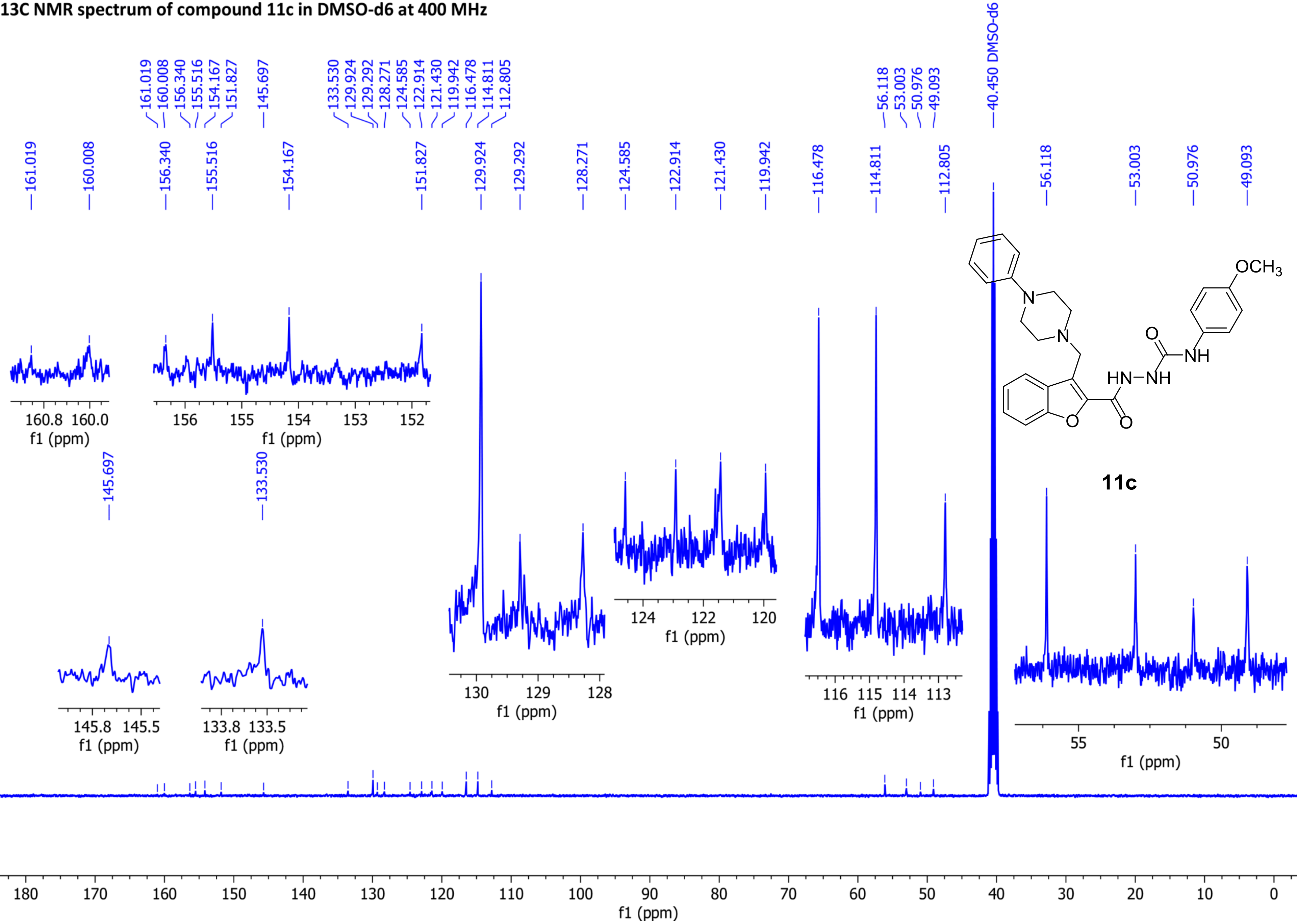

1H NMR spectrum of compound 11d in DMSO-d6 at 400 MHz

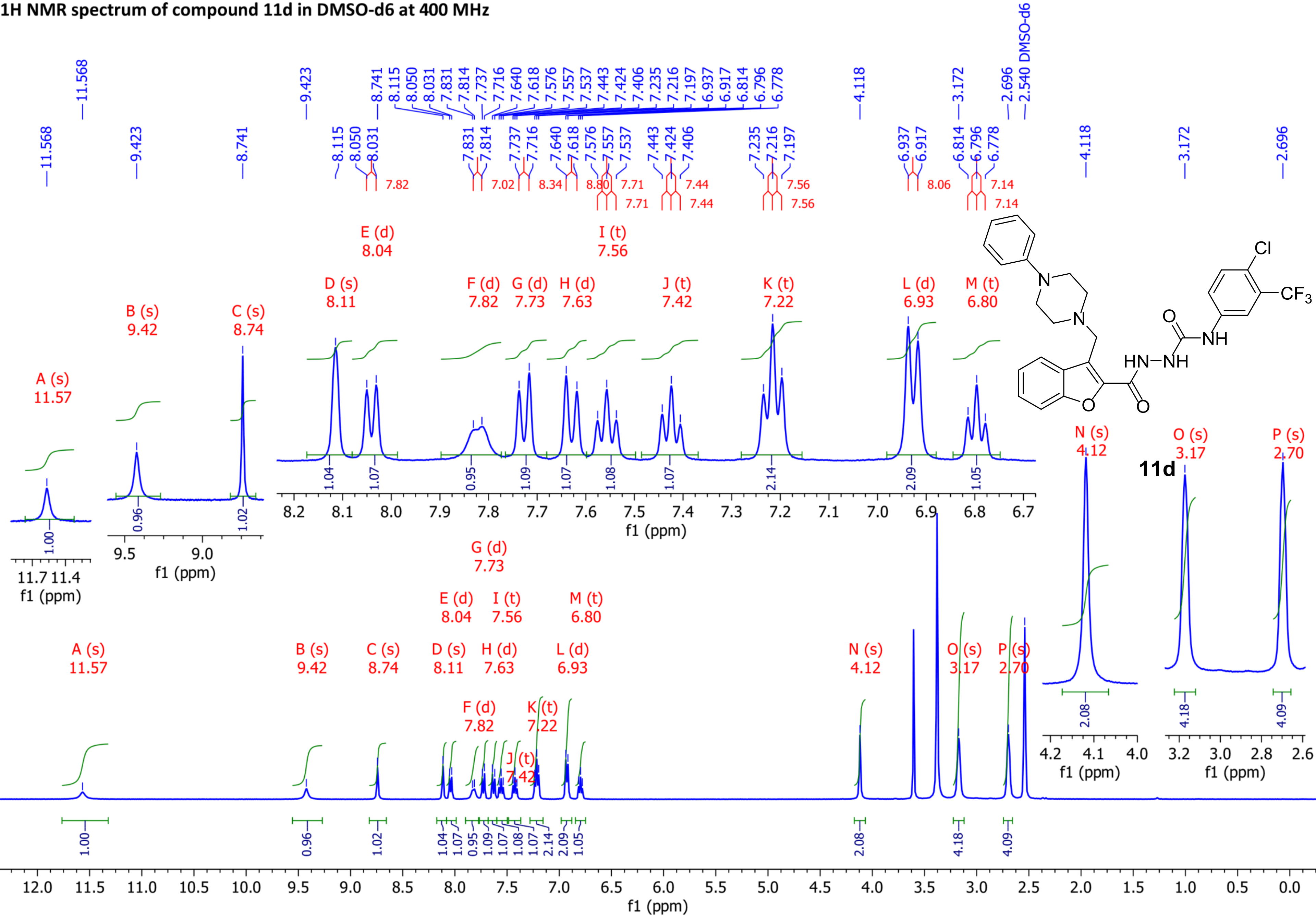

13C NMR spectrum of compound 11d in DMSO-d6 at 400 MHz

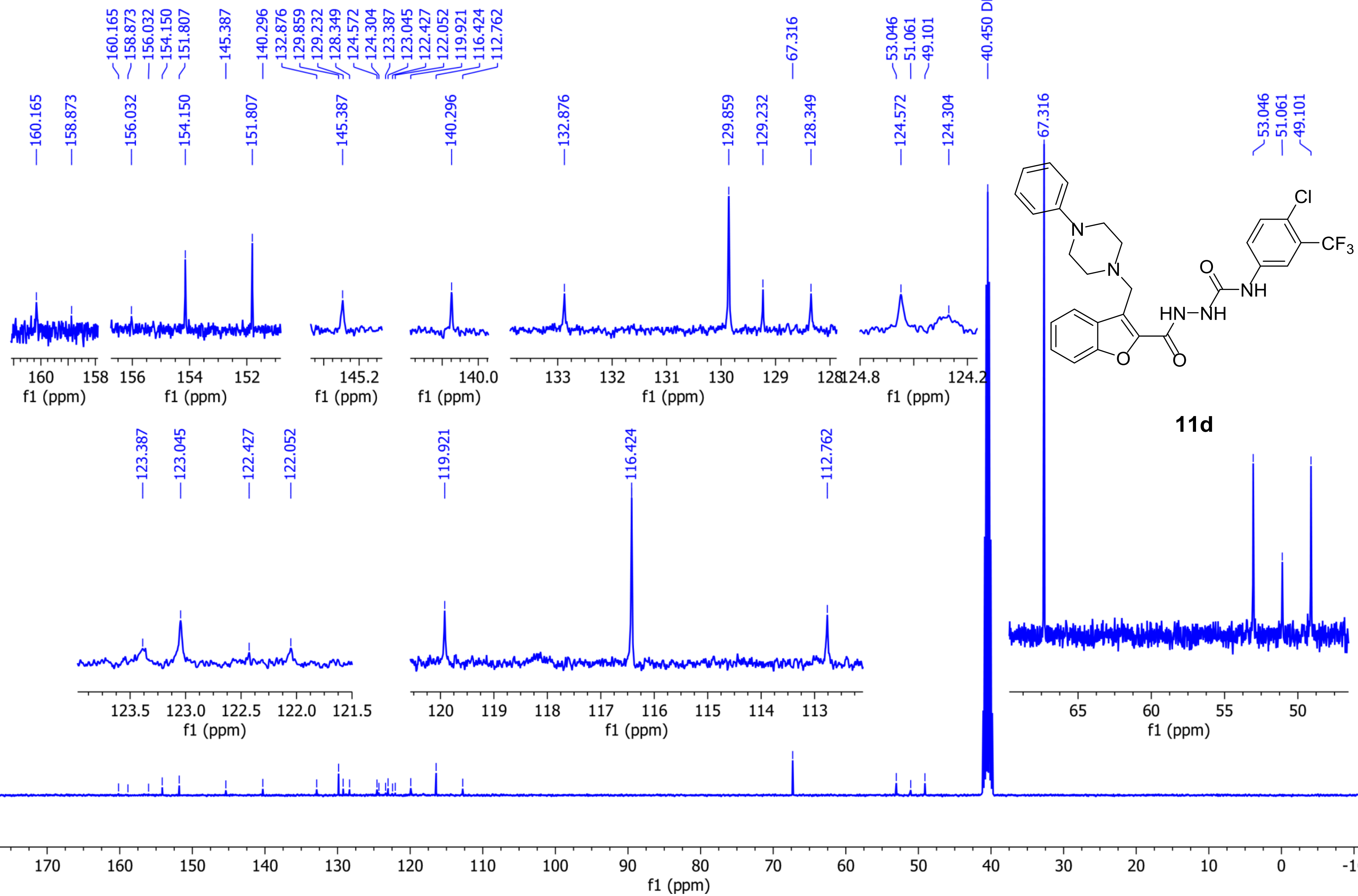

1H NMR spectrum of compound 11e in DMSO-d6 at 400 MHz

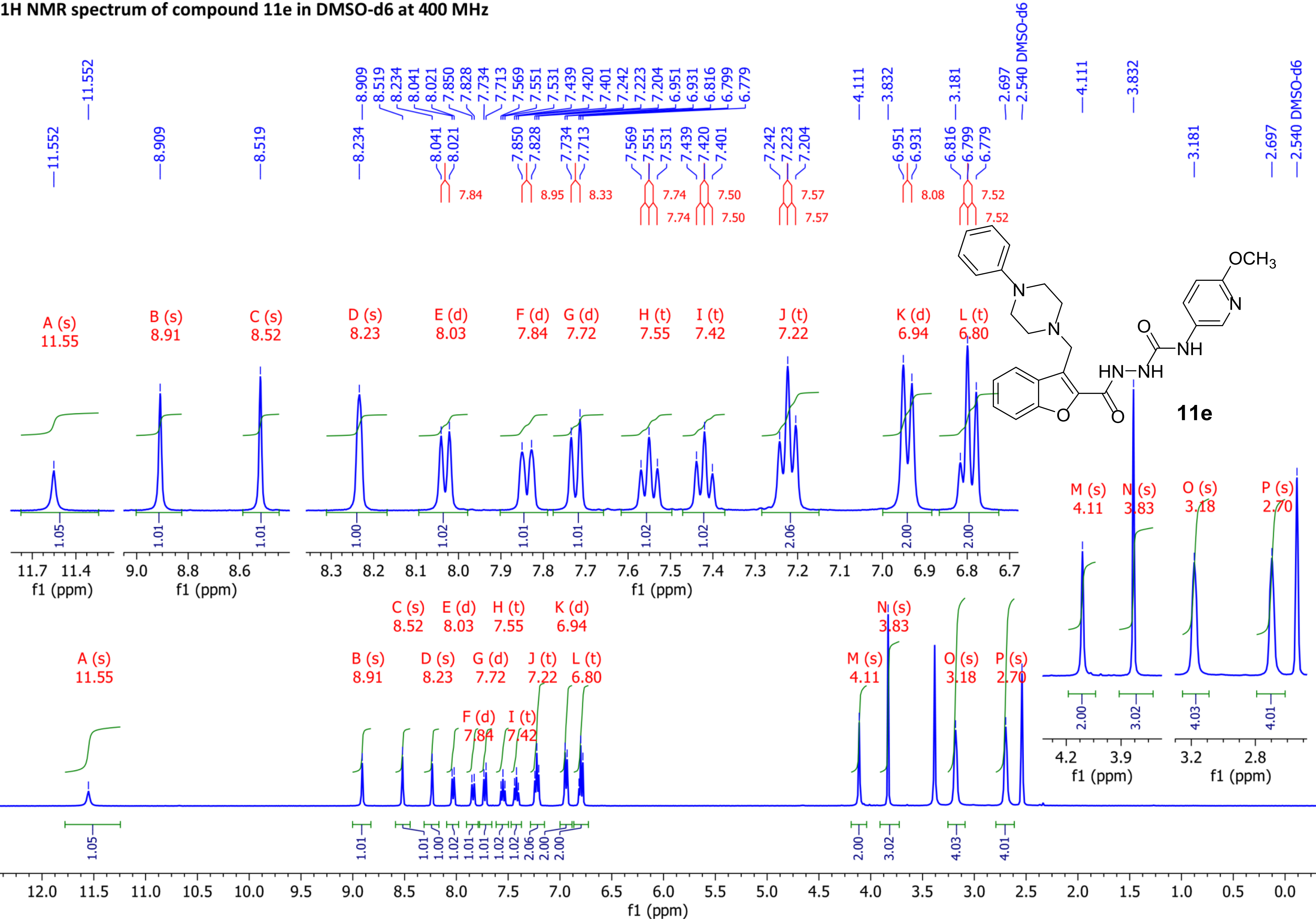

13C NMR spectrum of compound 11e in DMSO-d6 at 400 MHz

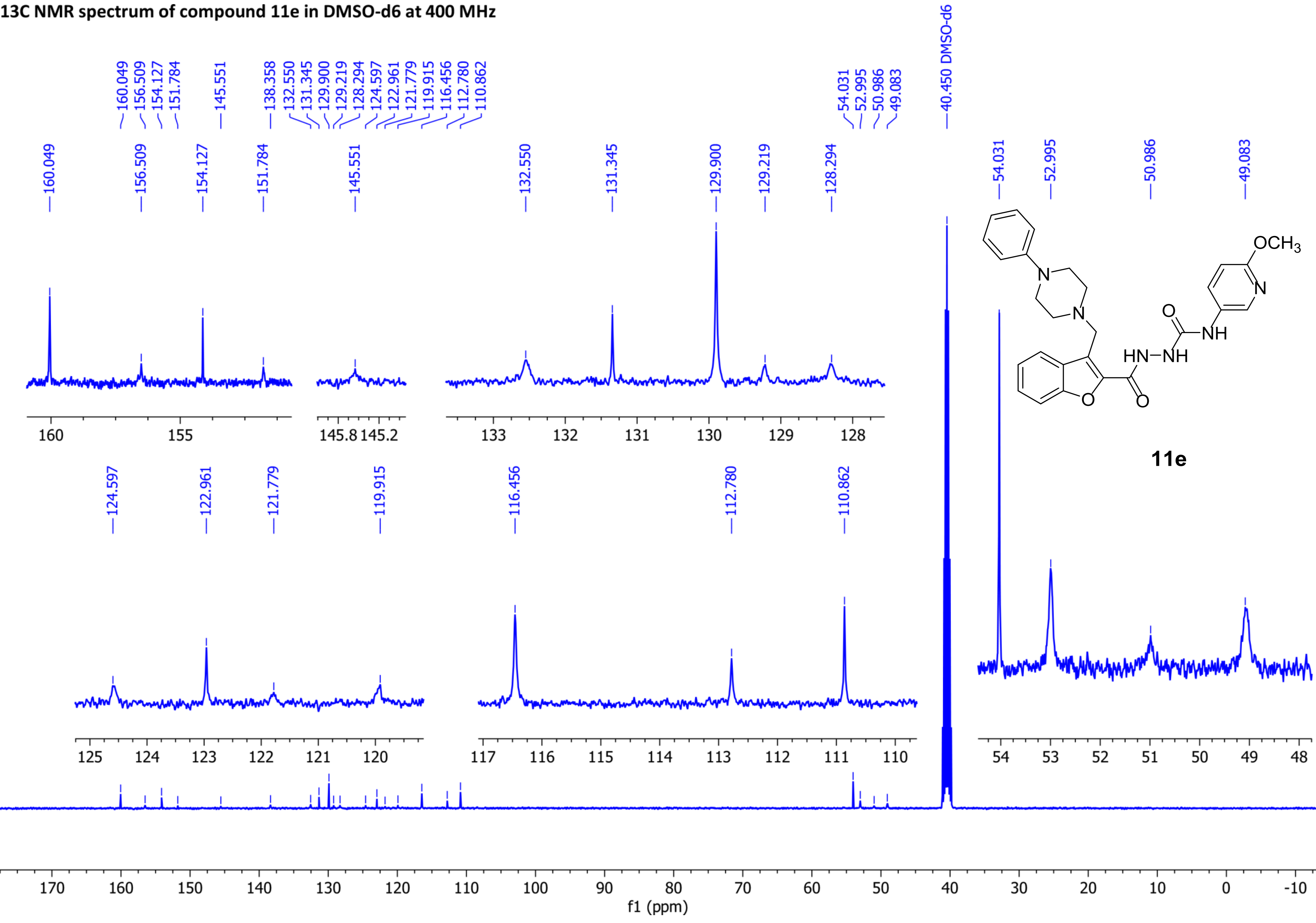

1H NMR spectrum of compound 13a in DMSO-d6 at 400 MHz

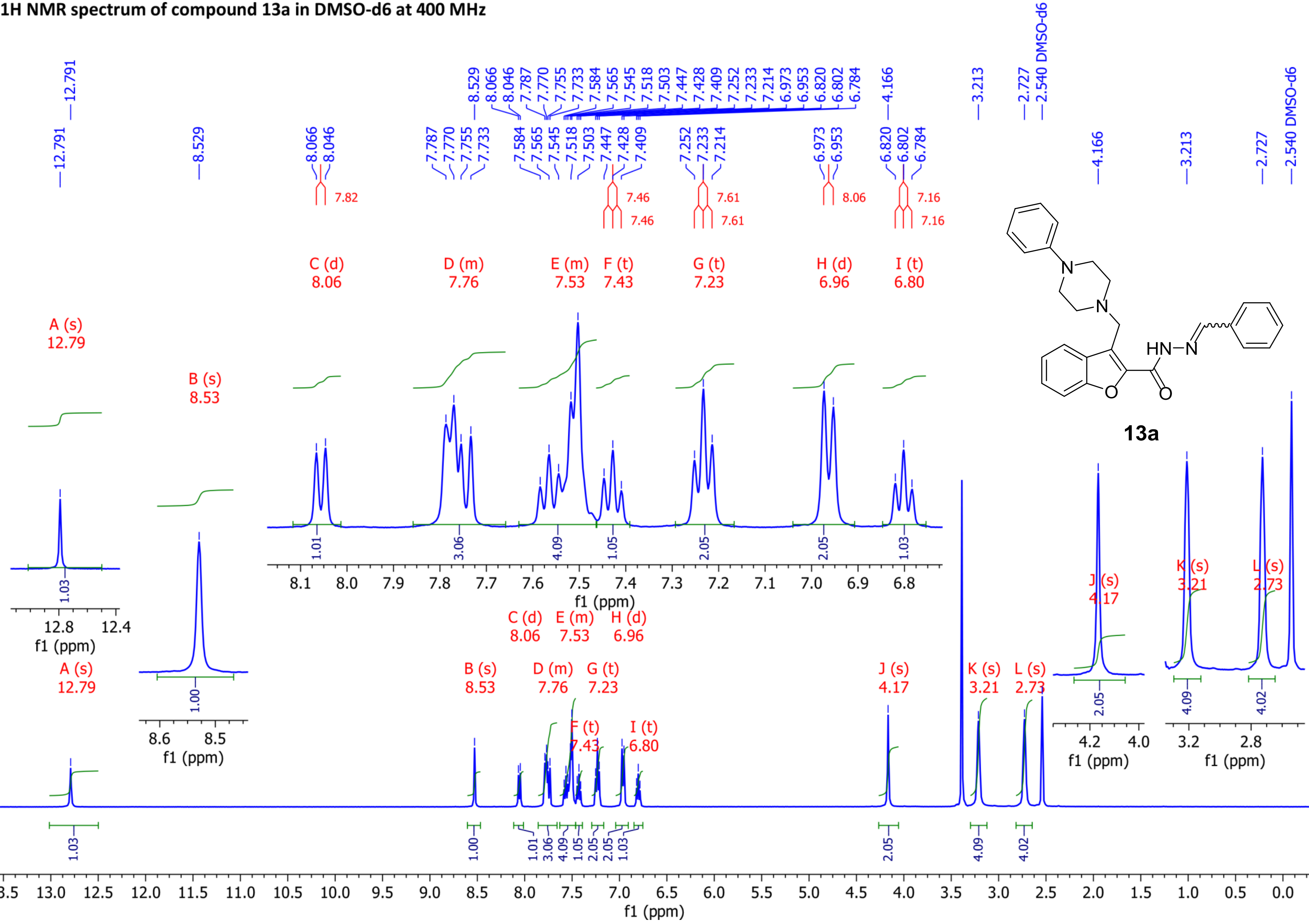

13C NMR spectrum of compound 13a in DMSO-d6 at 400 MHz

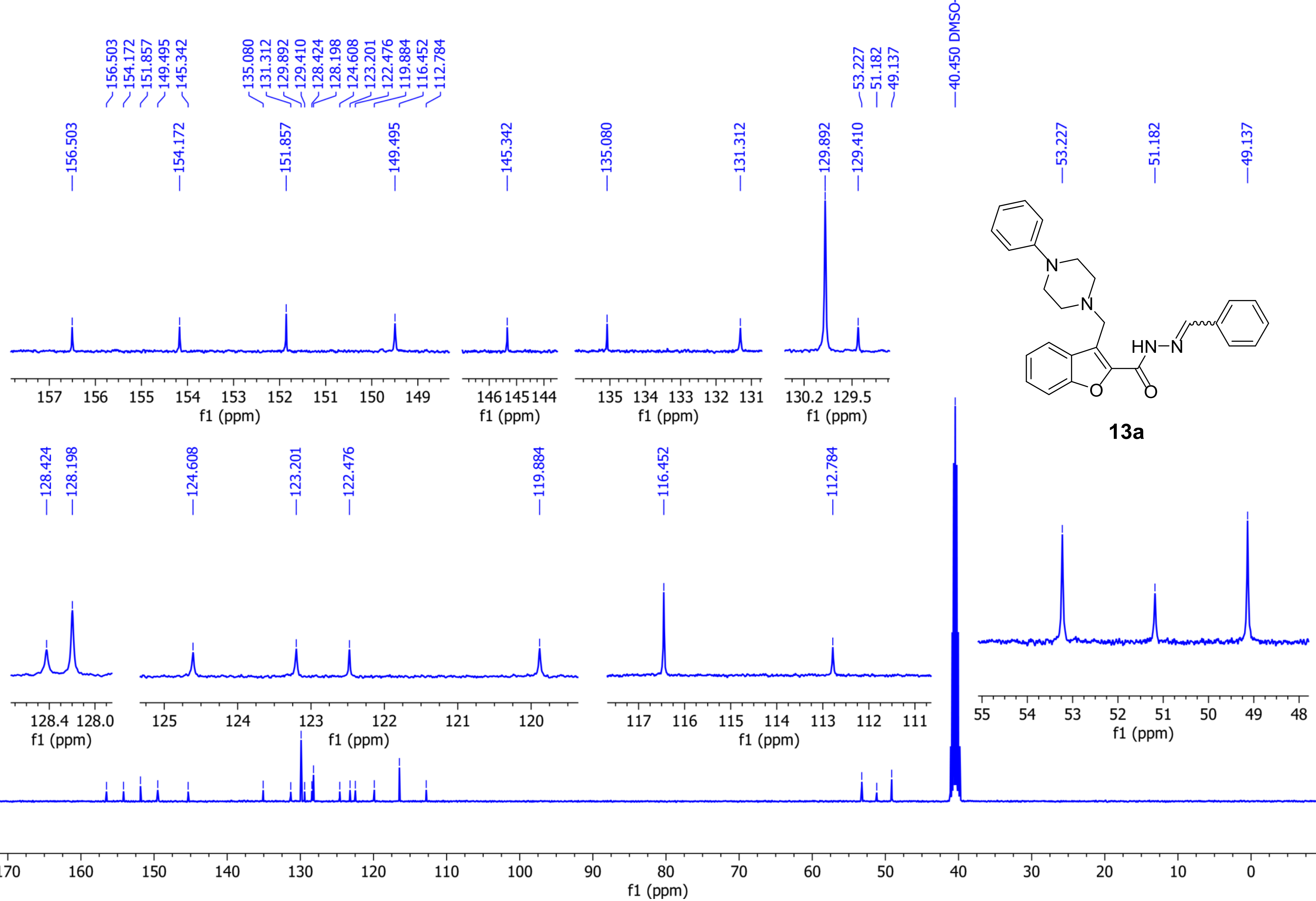

1H NMR spectrum of compound 13b in DMSO-d6 at 400 MHz

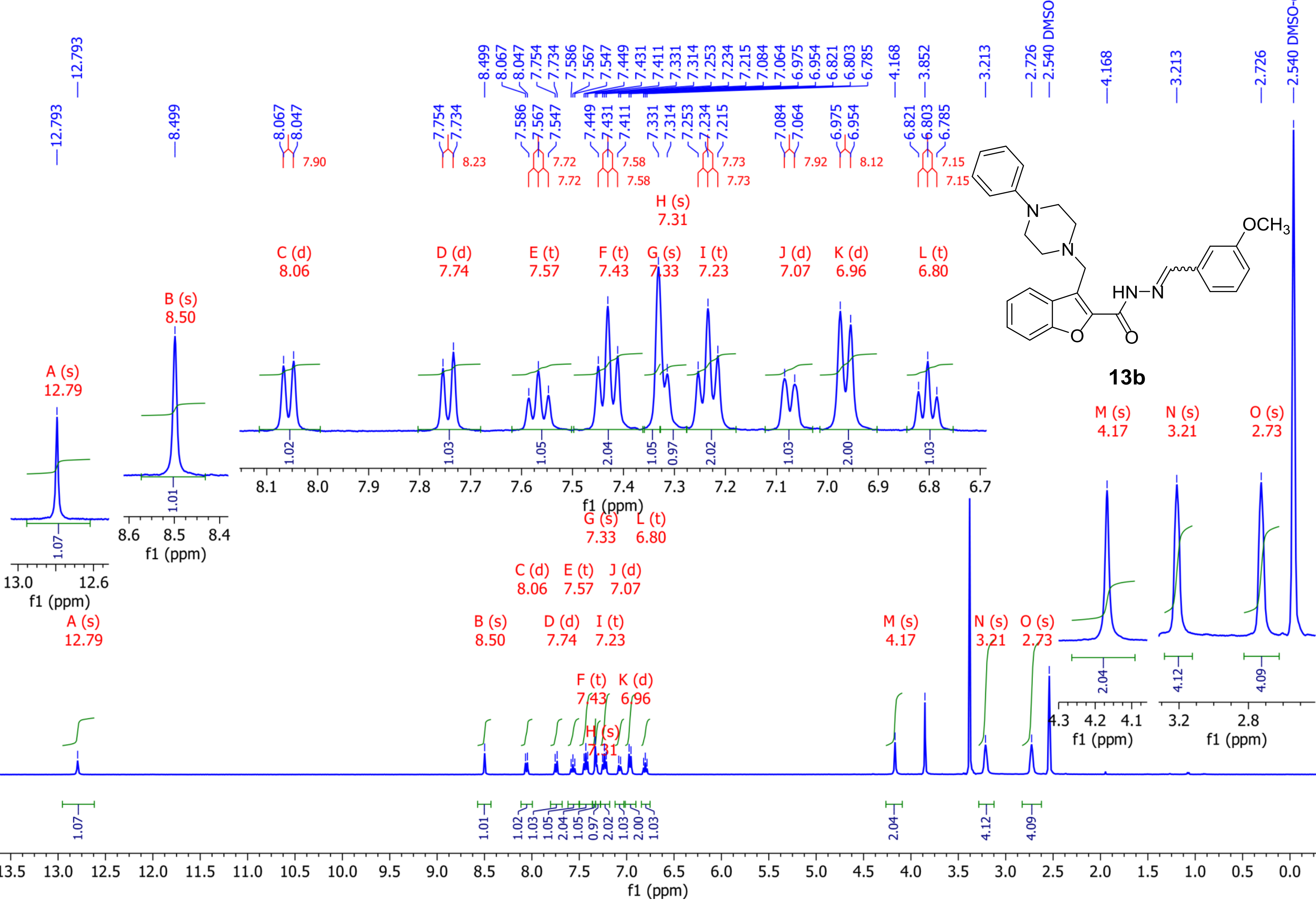

13C NMR spectrum of compound 13b in DMSO-d6 at 400 MHz

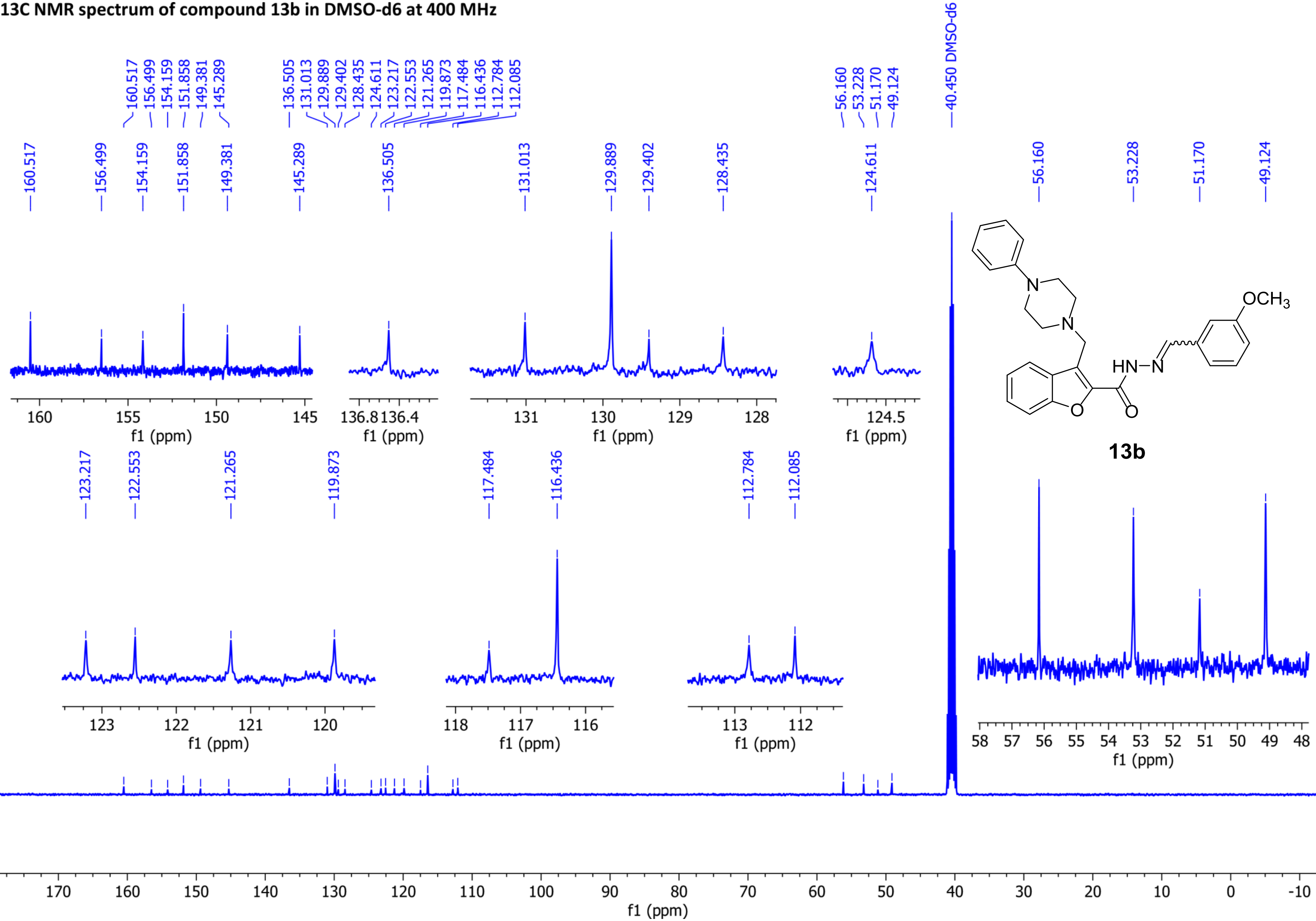

1H NMR spectrum of compound 13c in DMSO-d6 at 400 MHz

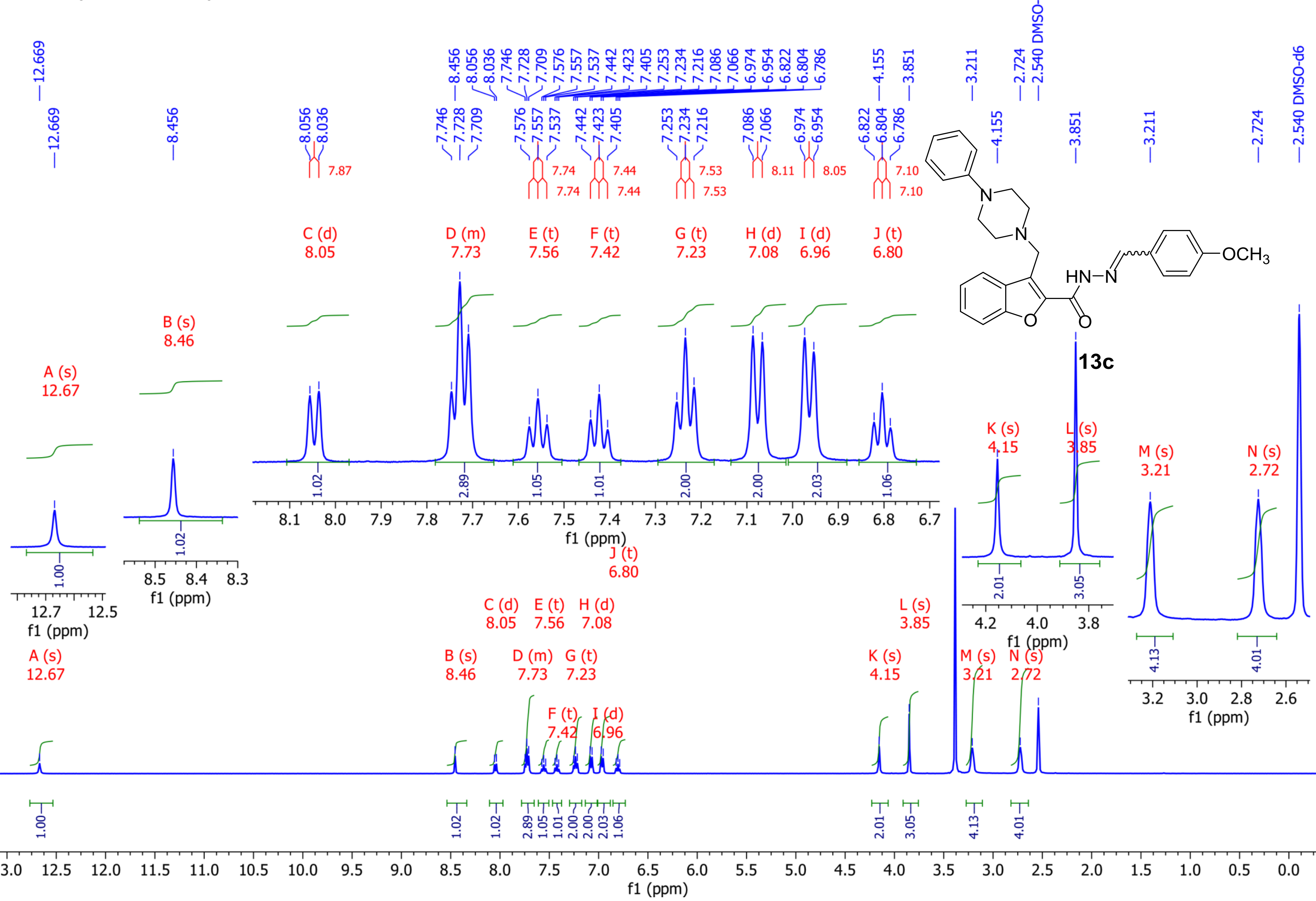

13C NMR spectrum of compound 13c in DMSO-d6 at 400 MHz

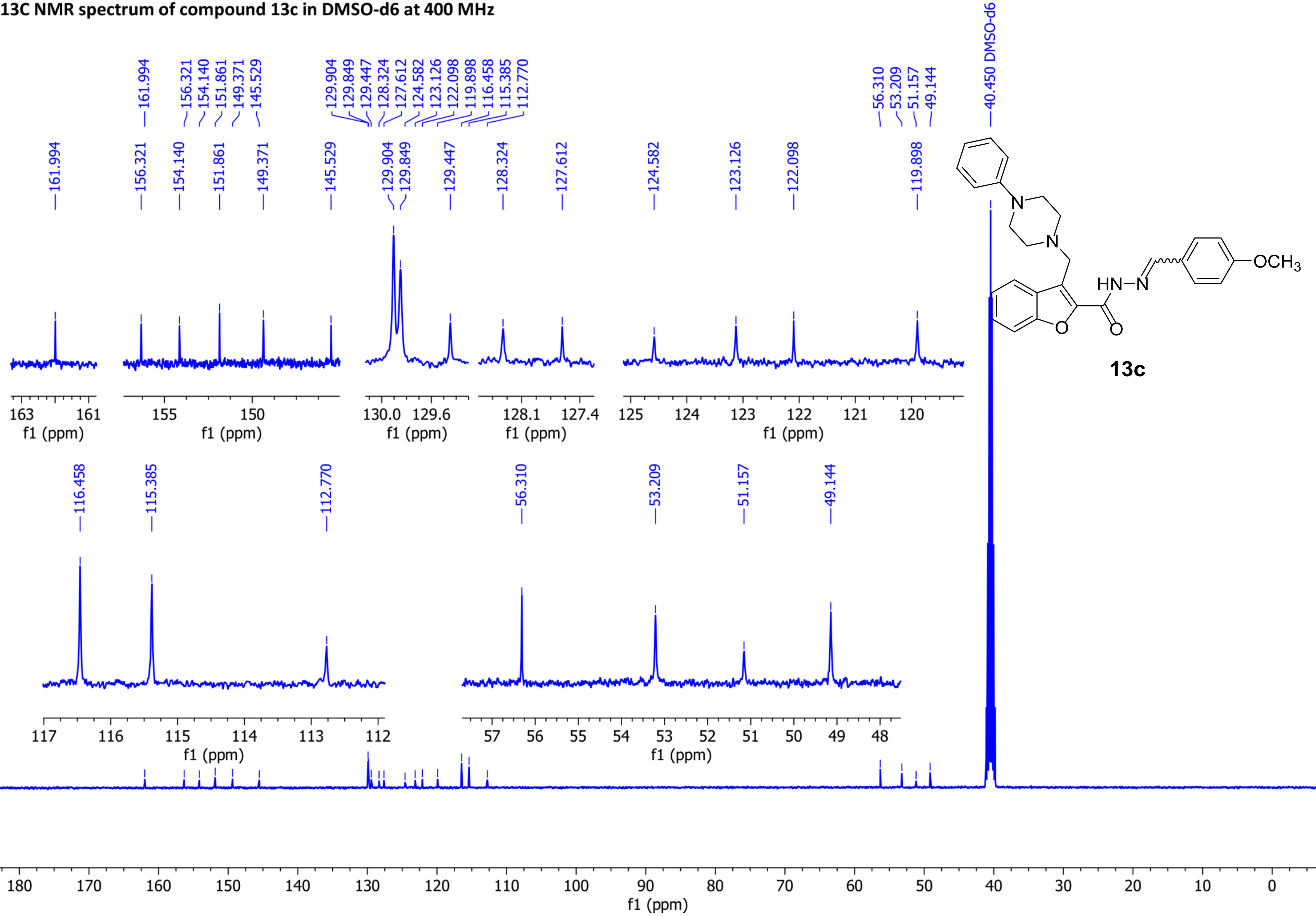

1H NMR spectrum of compound 15a in DMSO-d6 at 400 MHz

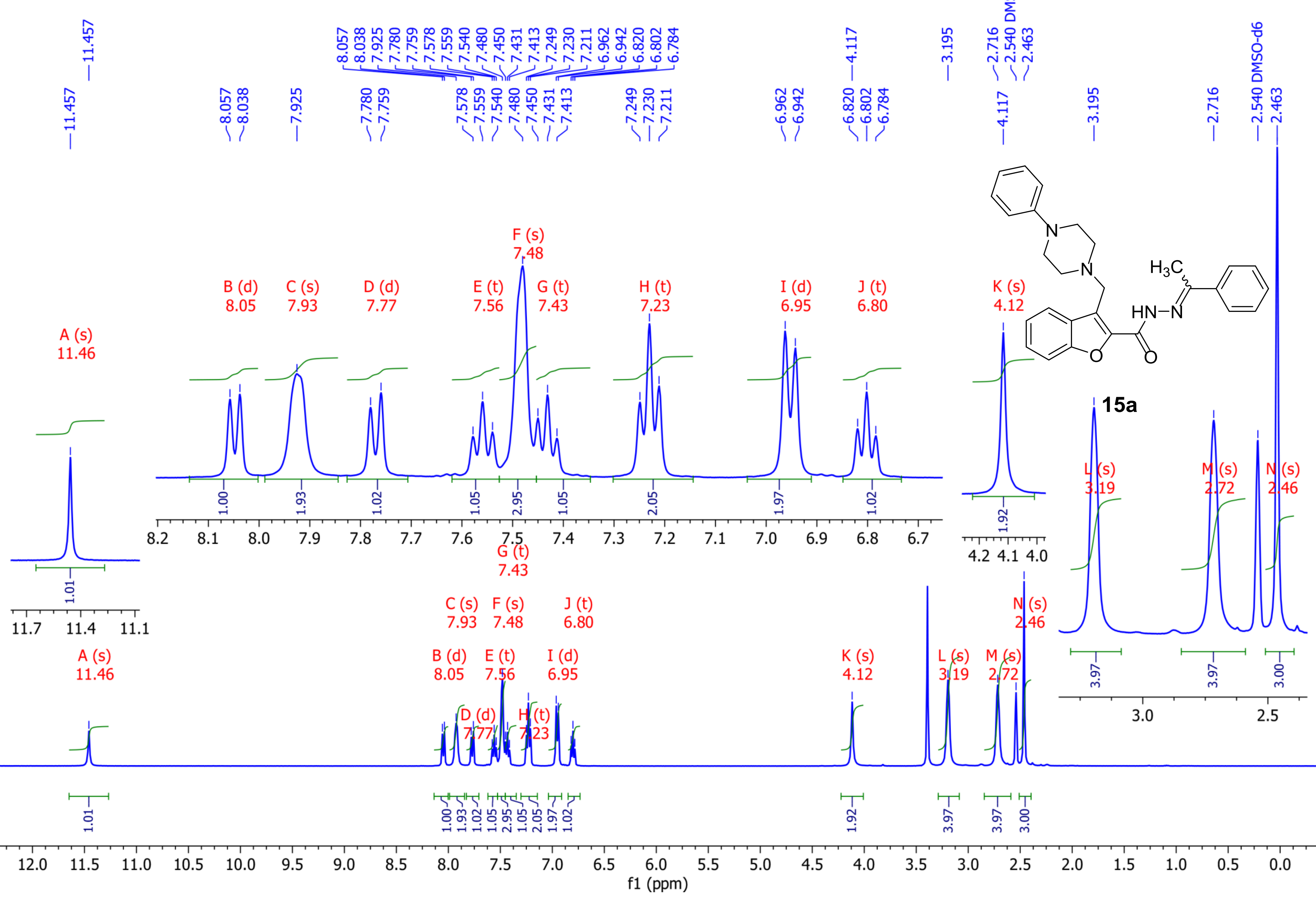

13C NMR spectrum of compound 15a in DMSO-d6 at 400 MHz

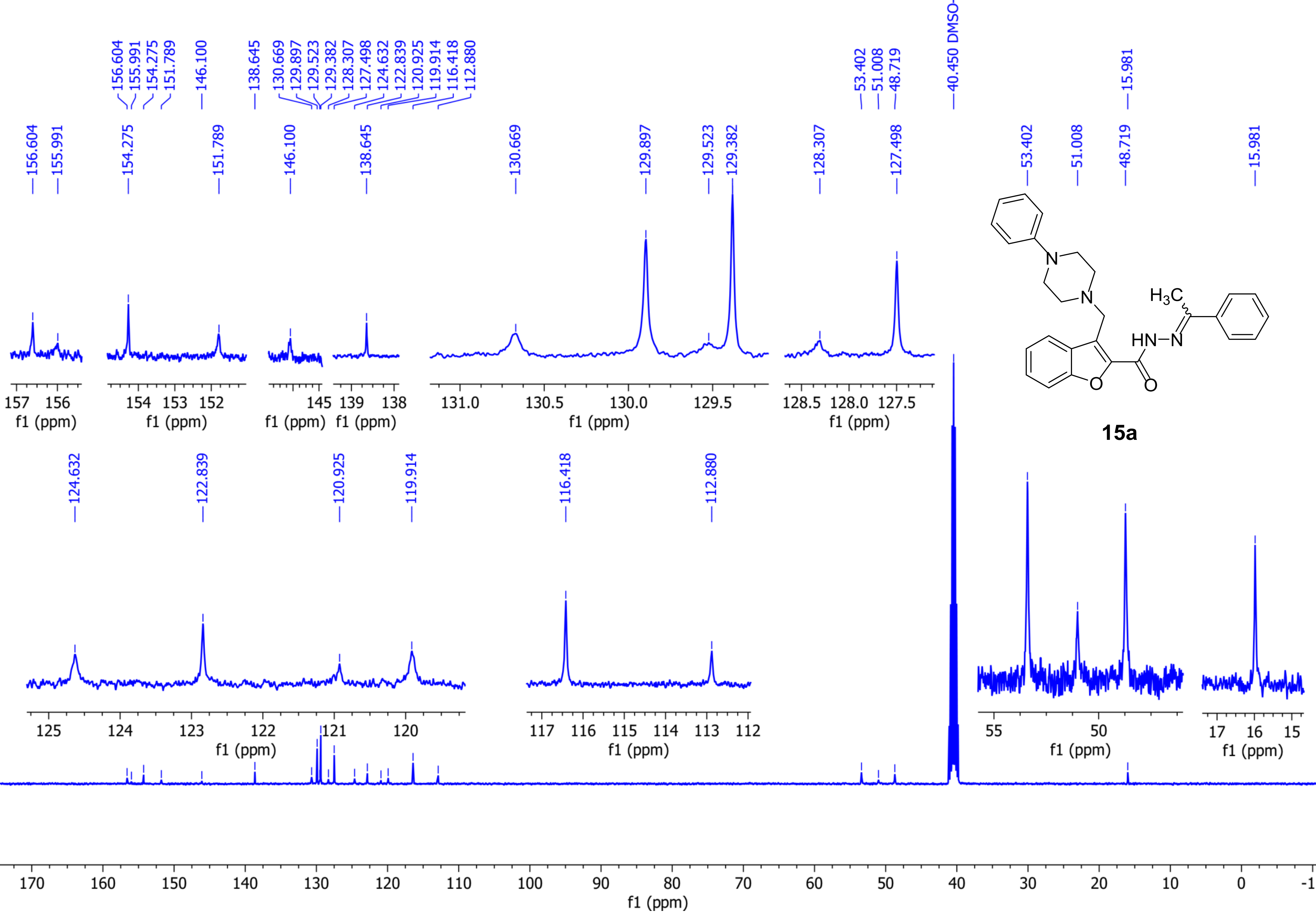

1H NMR spectrum of compound 15b in DMSO-d6 at 400 MHz

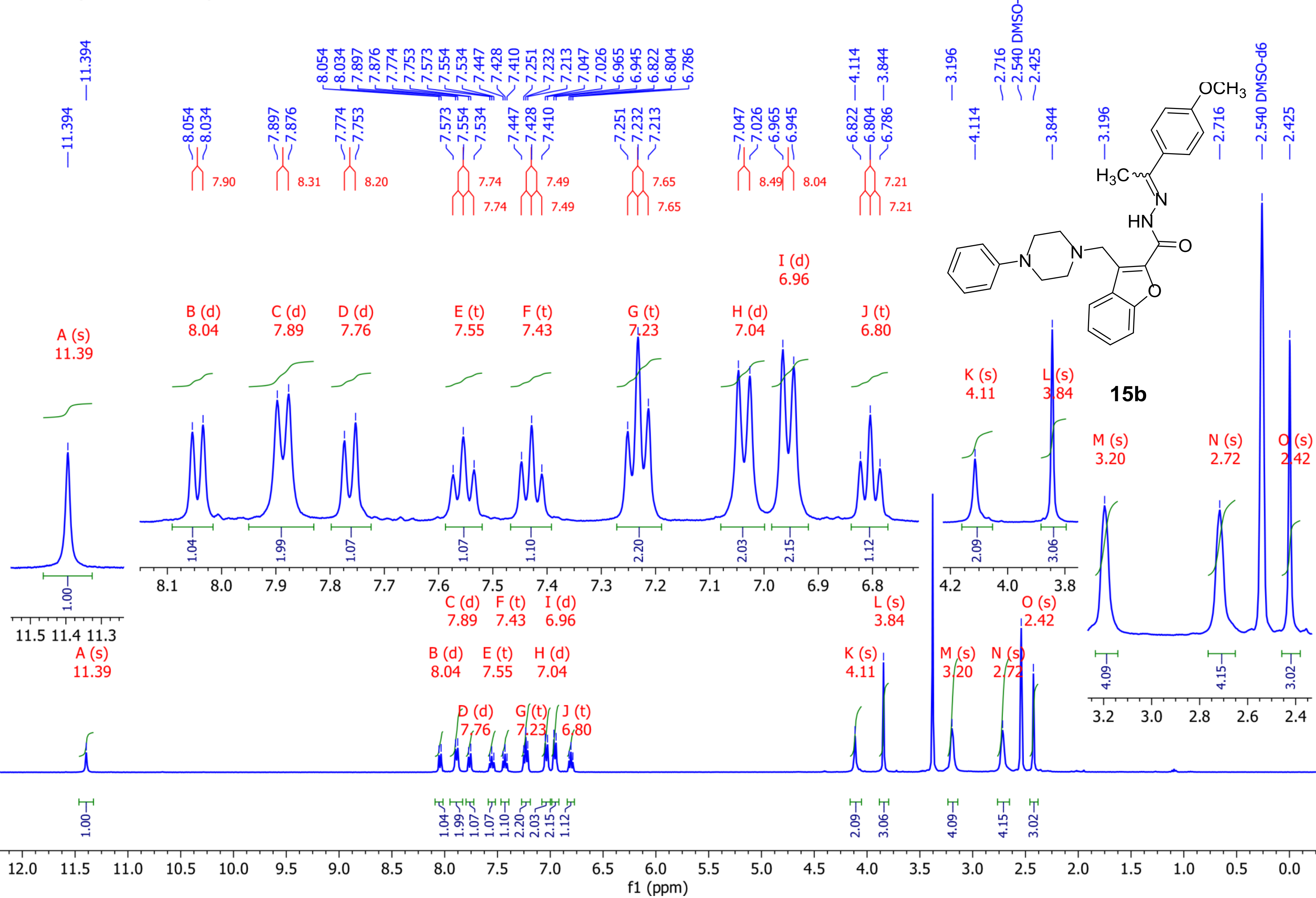

13C NMR spectrum of compound 15b in DMSO-d6 at 400 MHz

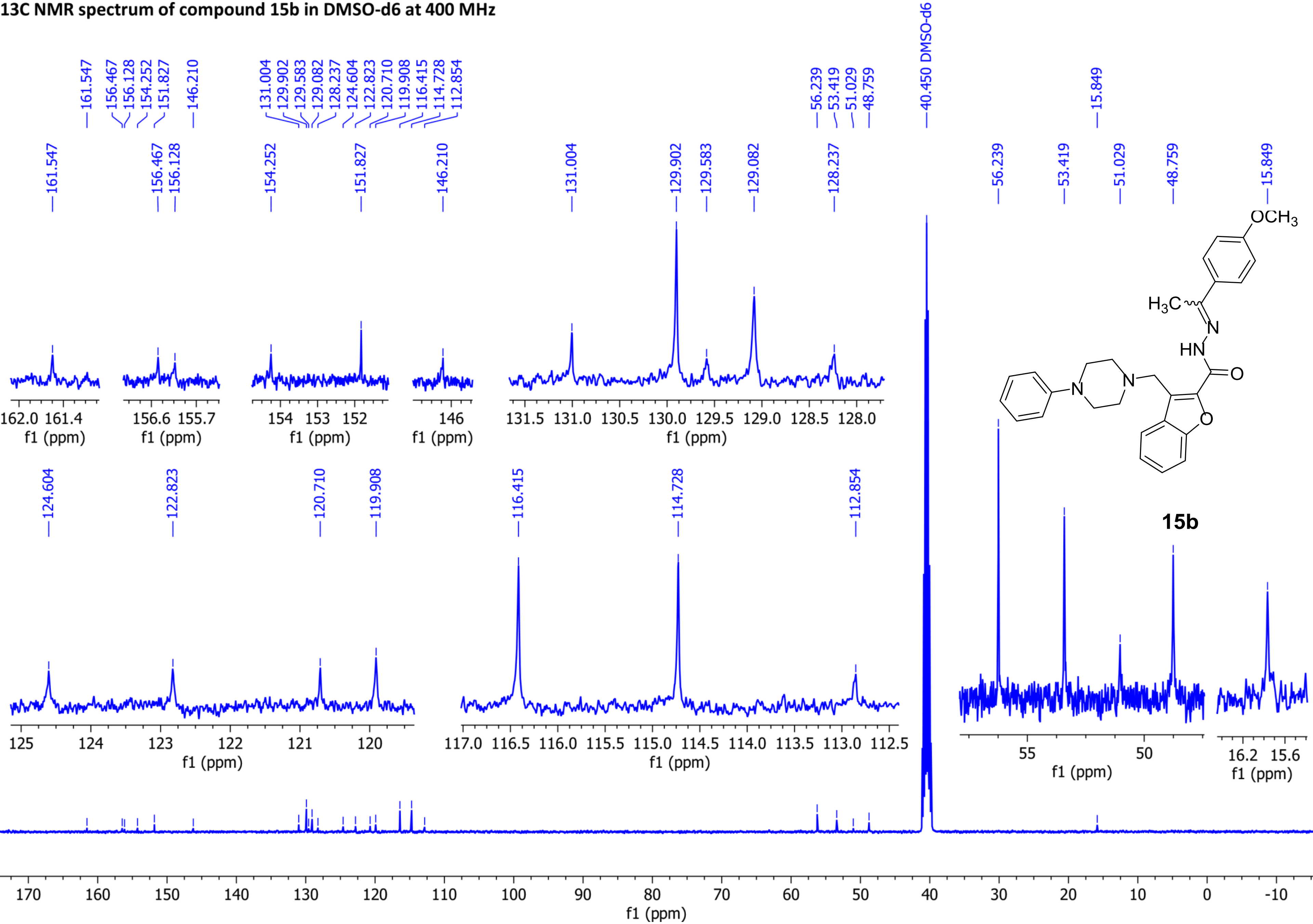

1H NMR spectrum of compound 17 in DMSO-d6 at 400 MHz

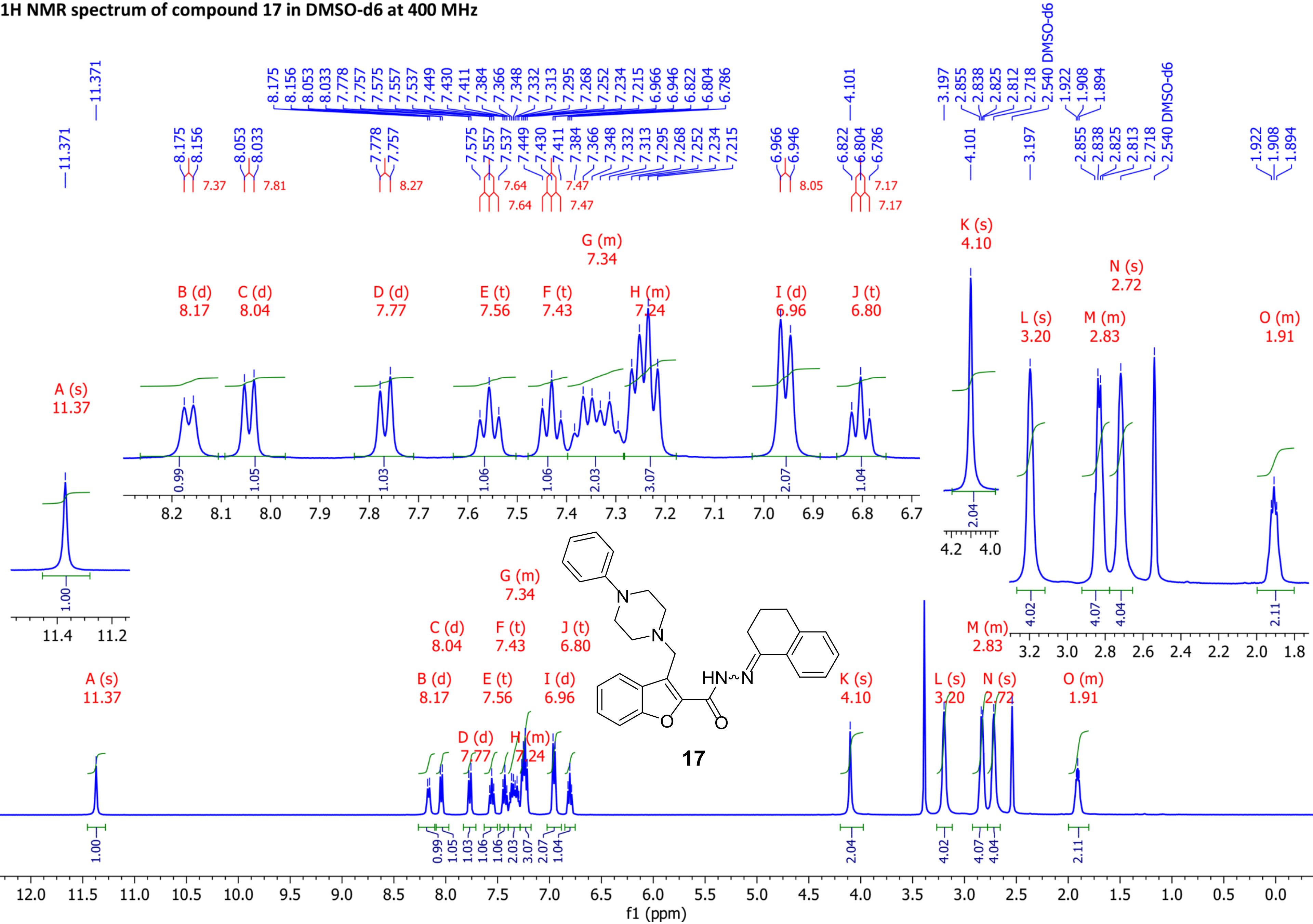

13C NMR spectrum of compound 17 in DMSO-d6 at 400 MHz

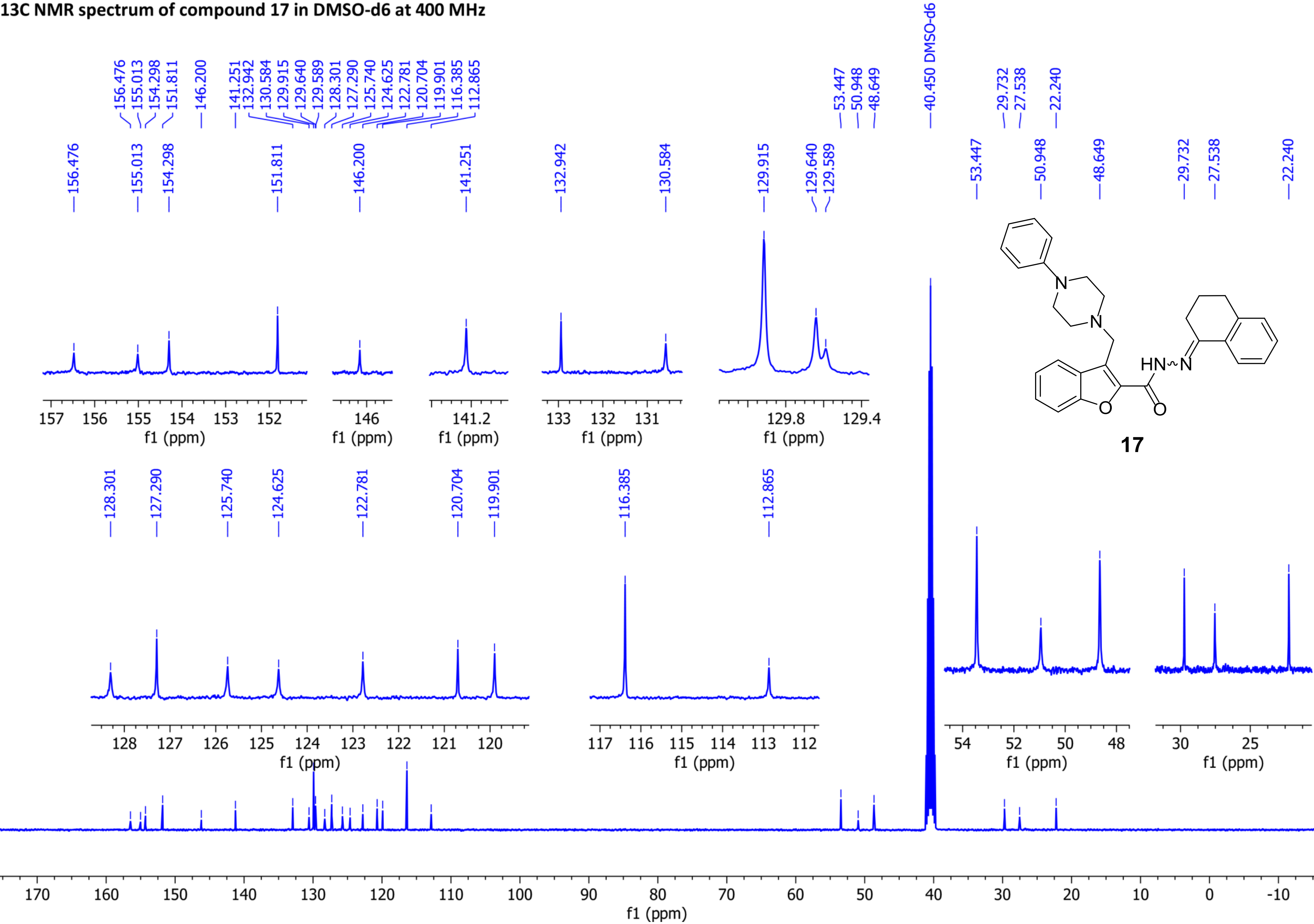

Supplement: Supplemental Material [file IENZ_A_2062337_SM7603.pdf]
